# Supplementary material for: Catalytic amidation of natural and synthetic polyol esters with sulfonamides
Source: Nat Commun. 2019 Aug 28;10:3881. doi: 10.1038/s41467-019-11864-9 (PMC6713792; doi:10.1038/s41467-019-11864-9)
Supplement: Supplementary file 1 — Supplementary Information [file 41467_2019_11864_MOESM1_ESM.pdf]

Supplementary Information

Catalytic Amidation of Natural and Synthetic Polyol Esters with Sulfonamides

Liu *et al.*

Supplementary Table 1. Conditions Optimization for Substitution of Ethylene Glycol Diacetate with TsNHMe[a]

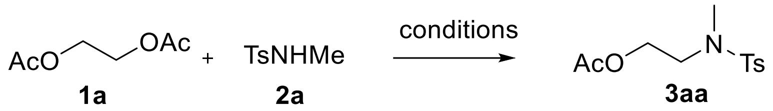

Entry	Catalyst	mol %	Solvent	Temp /°C	Time /h	Yield /%[b]
1	$Hf(OTf)_4$	2	DCE	120	24	80
2	$Fe(OTf)_3$	2	DCE	120	24	78
3	$Al(OTf)_3$	2	DCE	120	24	66
4	$Sc(OTf)_3$	2	DCE	120	24	56
5	$Cu(OTf)_2$	2	DCE	120	24	25
6	$Yb(OTf)_3$	2	DCE	120	24	25
7	$Bi(OTf)_3$	2	DCE	120	24	60
8	$Hf(OTf)_4$	2	PhMe	120	24	78
9	$Hf(OTf)_4$	2	PhCl	120	24	79
10	$Hf(OTf)_4$	2	EtOAc	120	24	58
11	$Hf(OTf)_4$	2	MeCN	120	24	0
**12**	**$Hf(OTf)_4$**	**2**	**neat**	**120**	**24**	**95**
13	$Hf(OTf)_4$	2	neat	120	6	90
14	$Hf(OTf)_4$	1	neat	120	24	87
15	$Hf(OTf)_4$	2	neat	120	24	91[c]
16	$Hf(OTf)_4$	2	neat	120	24	87[d]

[a] **1a** (1.0 mmol), **2a** (0.5 mmol), catalyst and solvent (0.5 mL if used) were stirred at 120 °C for specified time unless otherwise noted; [b] NMR yield; [c] 0.75 mmol **1a** was used; [d] 0.6 mmol **1a** was used.

Supplementary Table 2. Conditions Optimization for Substitution of Acetamidoethyl Acetate with Saccharin[a]

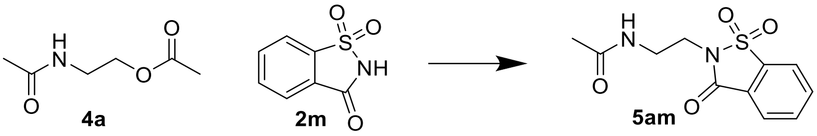

Entry	Catalyst	mol %	Solvent	Temp /°C	Time /h	Yield /%[b]
1	$Hf(OTf)_4$	2	PhCl	120	12	48
2	$Fe(OTf)_3$	2	PhCl	120	12	45
3	$Al(OTf)_3$	2	PhCl	120	12	45
4	$Sc(OTf)_3$	2	PhCl	120	12	42
5	$La(OTf)_3$	2	PhCl	120	12	28
6	$Yb(OTf)_3$	2	PhCl	120	12	38
7	$Bi(OTf)_3$	2	PhCl	120	12	36
8	$Hf(OTf)_4$	2	PhMe	120	12	45
9	$Hf(OTf)_4$	2	DCE	120	12	40
10	$Hf(OTf)_4$	2	EtOAc	120	12	35
11	$Hf(OTf)_4$	2	neat	120	12	68
12	$Hf(OTf)_4$	2	neat	150	12	88
13	$Hf(OTf)_4$	2	neat	150	24	95
14	$Hf(OTf)_4$	1	neat	150	24	95
15	$Hf(OTf)_4$	0.5	neat	150	24	84
**16**	**$Hf(OTf)_4$**	**1**	**neat**	**150**	**24**	**94**[c]

[a] **4a** (1.0 mmol), **2m** (0.5 mmol), catalyst and solvent (0.5 mL if used) were stirred at specified temperature for specified time unless otherwise noted; [b] NMR yield; [c] 0.6 mmol **4a** was used.

Supplementary Figure 1.

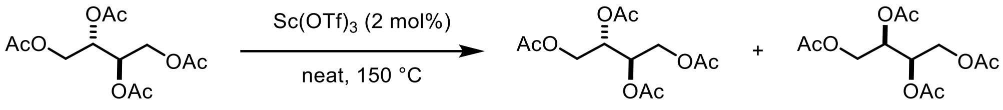

Erythritol acetate, 1p **Erythritol acetate, 1p** **(±)-Threitol acetate**

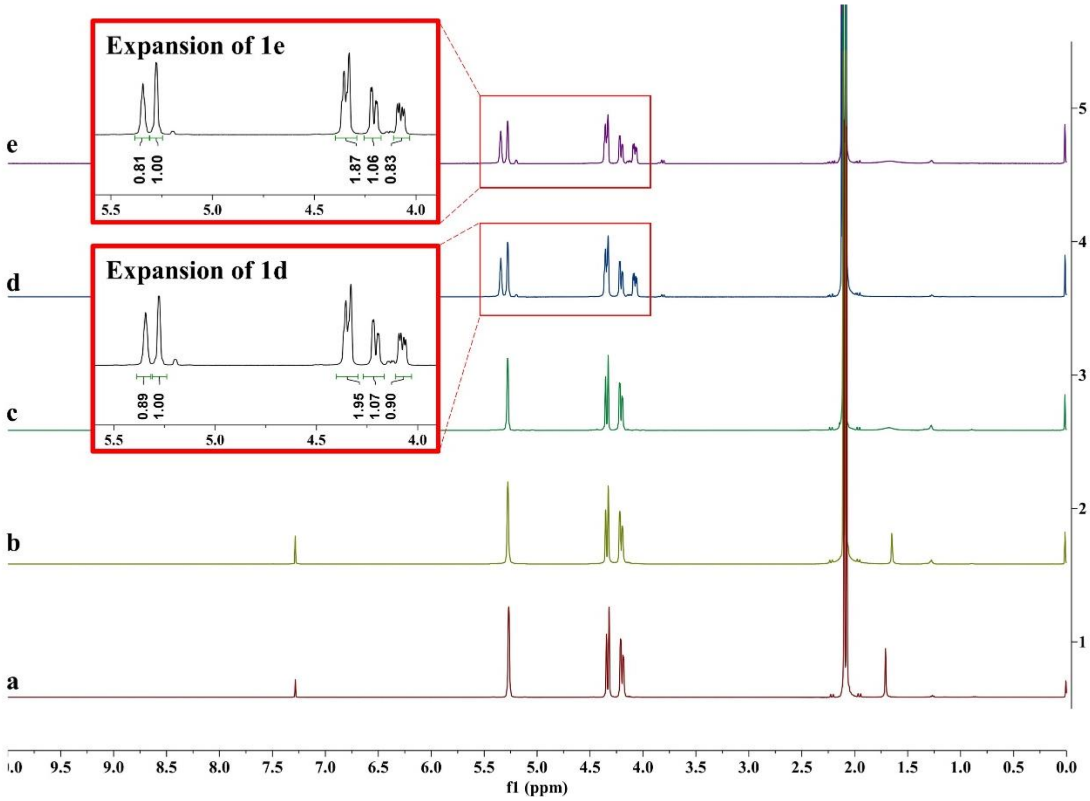

Stereochemistry Scrambling of Acetylated Erythritol without Nucleophile. Reaction conditions: erythritol acetate (**1p**, 1.0 mmol) and $Sc(OTf)_3$ (2 mol %) were stirred at 150 °C and monitored by ^{1}H-NMR of reaction aliquot. (a) Pristine **1p** before reaction starts. (b) Heating **1p** alone for 1 h, no change observed. (c) Heating the mixture for 5 min after addition of Lewis acid: no isomerization yet; (d) Heating the mixture for 1 h: significant isomerization to threitol acetate[1] showing a d.r. of 0.89:1; (e) Heating the mixture for 5 h: no significant progress since 1 h, probably reaching the equilibrium.

Supplementary Figure 2.

a

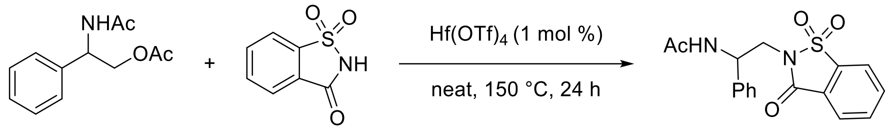

(*R*)-**4d** or (±)-**4d** **2m** (*R*)-**5dm** or (±)-**5dm**

b HPLC analysis of **4d**:

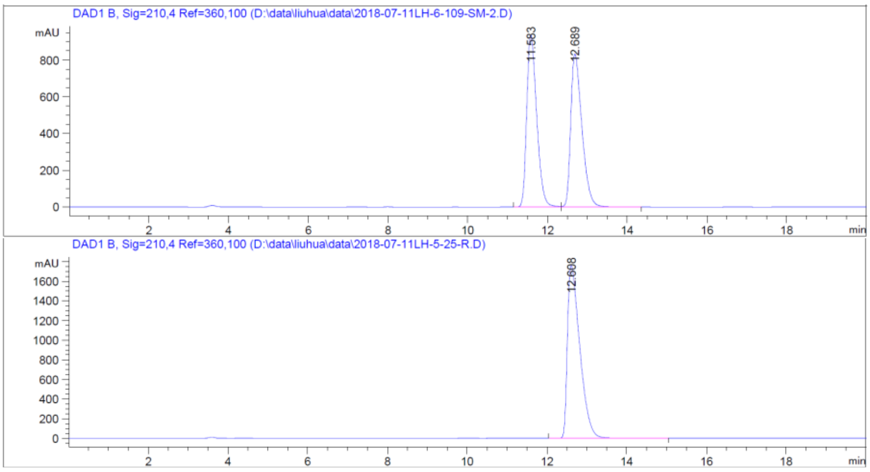

Peak #	RetTime [min]	Type	Width [min]	Area [mAU*s]	Height [mAU]	Area %
1	12.608	BB	0.3275	3.78890e4	1757.64233	100.0000

c HPLC analysis of **5dm**:

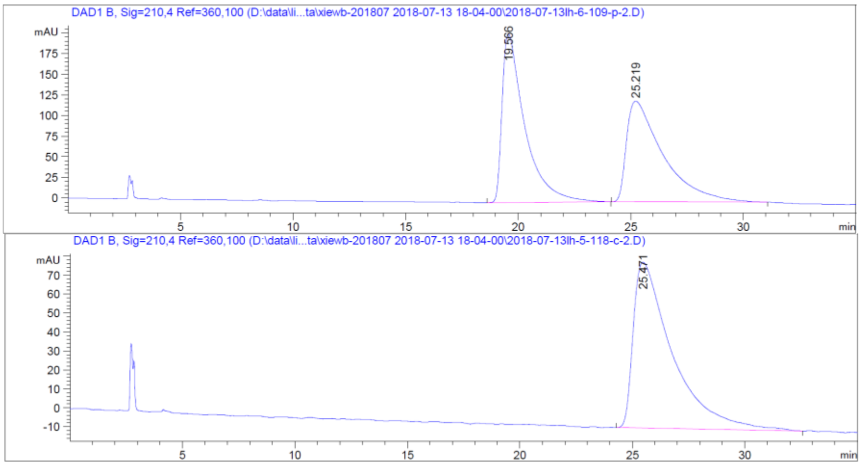

Peak #	RetTime [min]	Type	Width [min]	Area [mAU*s]	Height [mAU]	Area %
1	25.471	BB	1.6687	1.05401e4	87.41312	100.0000

Retention of Configuration of the Amide α-Carbon. **a** (*R*)-**4d** was chosen as a model to investigate whether the stereochemistry was changed. **b** HPLC traces of (*rac*)-**4d** and (*R*)-**4d**. **c** HPLC traces of (*rac*)-**5dm** and (*R*)-**5dm**.

Supplementary Figure 3:

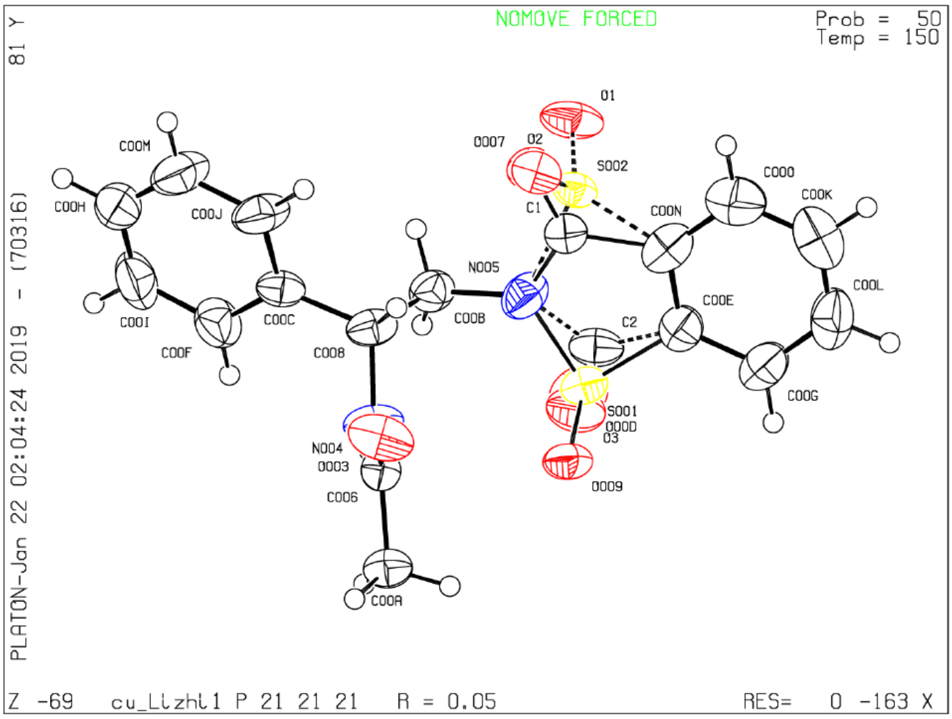

Single-crystal XRD for (*R*)-5dm. Deposited to CCDC No.: CCDC 1892474.

Supplementary Table 3. Crystal data and structure refinement for (*R*)-5dm.

Identification code	cu_Lizhi1	
Empirical formula	C3.24 H3.05 N0.38 O0.76 S0.19	
Formula weight	65.60	
Temperature	150(2) K	
Wavelength	1.54178 Å	
Crystal system	Orthorhombic	
Space group	$P2_12_12_1$	
Unit cell dimensions	a = 4.9490(2) Å	α= 90°.
	b = 17.9127(6) Å	β= 90°.
	c = 18.6968(7) Å	γ = 90°.
Volume	1657.47(11) $Å^3$	
Z	21	
Density (calculated)	1.380 Mg/m^3	
Absorption coefficient	1.948 mm^{-1}	
F(000)	720	
Crystal size	0.500 x 0.100 x 0.100 mm^3	
Theta range for data collection	3.417 to 77.680°.	
Index ranges	-6<=h<=6, -22<=k<=20, -23<=l<=22	
Reflections collected	17644	
Independent reflections	3495 [R(int) = 0.0676]	
Completeness to theta = 67.679°	99.4 %	
Absorption correction	Semi-empirical from equivalents	
Max. and min. transmission	0.7541 and 0.4882	
Refinement method	Full-matrix least-squares on F^2	
Data / restraints / parameters	3495 / 2 / 264	
Goodness-of-fit on F^2	1.042	
Final R indices [I>2sigma(I)]	R1 = 0.0474, wR2 = 0.1134	
R indices (all data)	R1 = 0.0602, wR2 = 0.1252	
Absolute structure parameter	0.033(14)	
Extinction coefficient	n/a	
Largest diff. peak and hole	0.177 and -0.240 e.$Å^{-3}$	

Supplementary Methods.

Commercially available substrates were used as received unless otherwise noted. Unavailable substrates were synthesized by acylation of corresponding alcohols or aminol alcohols with acid anhydride or acyl chlorides. Alcohols or aminol alcohols were obtained from commercial vendors and used as received unless otherwise noted.

All manipulations of reagents were carried out under air atmosphere. Reactions were carried out in capped cylindrical vials equipped with a magnetic stir bar and heated in an oil bath or a heating block. Reactions were monitored with thin-layer chromatography (TLC) plates precoated with 250 μm of fluorescence indicator-doped silica. Preparative TLC plates were precoated with 500 μm of fluorescence indicator-doped silica. Visualization of these plates was done by 254 nm UV lamp and/or $KMnO_4$ stain. Flash column chromatography was done on Biotage Isolera One using mixtures of petroleum ether (PE) and ethyl acetate (EA) as eluents.

NMR spectra were recorded on a Bruker Avance III HD spectrometer (FT, 500 MHz for ^{1}H, 126 MHz for ^{13}C). Chemical shifts (δ) for ^{1}H, and ^{13}C are referenced to internal solvent. Signal patterns are indicated as s, singlet; d, doublet; t, triplet, q, quartet; and m, multiplet. High-resolution mass spectra (HRMS) were obtained from ThermoFisher Q-Exactive Focus in electrospray ionization (ESI^+ or ESI^-) mode. HPLC data were obtained on Agilent Technologies 1260 Infinity equipped with DAD detector. X-Ray structure analyses was performed by Bruker D8 Venture X-ray single crystal diffractometer. Elemental analyses were performed by CHNS/O Element Analyser PerkinElmer 2400 Series II.

Procedure for the preparation of polyol esters and amide esters

Triethylamine (1.5 equivalent per hydroxyl and amino unit) and *N*,*N*-dimethylaminopyridine (1 mol % per hydroxyl and amino unit) were added to a solution of polyol or amino alcohol (20 mmol) in dichloromethane (50 mL). Acid anhydride or acyl chloride (1.2 equivalent per hydroxyl and amino unit) was then added dropwise to the stirred solution in an ice-water bath. The resulting solution was warmed to room temperature and stirred until TLC analysis showed no remaining starting material. The reaction mixture was transferred to a separatory funnel and washed sequentially with water (50 mL), 1 M HCl aqueous solution (50 mL) and saturated aqueous NaCl solution (50 mL). The organic phase was dried with sodium sulfate (Na_2SO_4) and concentrated in vacuo. The crude product was purified by flash column chromatography to afford the corresponding polyol ester (PE : EA = 10 : 1) or amide ester (PE : EA = 1 : 1).

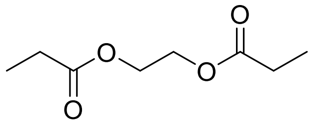

Ethane-1,2-diyl dipropionate[2] (1b): Ethylene glycol and propionyl chloride were used. Colorless liquid, 75% yield. ^{1}H NMR (500 MHz, $CDCl_3$) δ 4.29 (s, 4H), 2.37 (q, *J* = 7.6 Hz, 4H), 1.16 (t, *J* = 7.6 Hz, 6H).

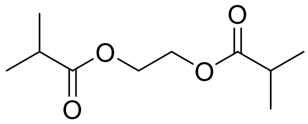

Ethane-1,2-diyl bis(2-methylpropanoate)[3] (1c): Ethylene glycol and isobutyryl chloride were used. Colorless liquid, 68% yield. ^{1}H NMR (500 MHz, $CDCl_3$) δ 4.28 (s, 4H), 2.61 – 2.52 (m, 2H), 1.17 (d, *J* = 7.0 Hz, 12H).

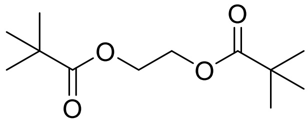

Ethane-1,2-diyl bis(2,2-dimethylpropanoate) (1d): Ethylene glycol and pivalyl chloride were used. Colorless liquid, 74% yield. ^{1}H NMR (500 MHz, $CDCl_3$) δ 4.27 (s, 4H), 1.20 (s, 18H). ^{13}C NMR (126 MHz, $CDCl_3$) δ 178.2, 62.1, 38.7, 27.1. HR-MS (ESI-TOF) calcd for $C_{12}H_{26}NO_4^+$[M+NH$_4$]$^+$: 248.1856, found 248.1852.

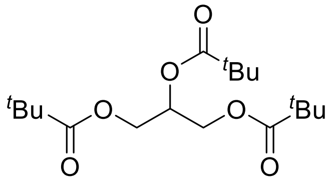

Propane-1,2,3-triyl tris(2,2-dimethylpropanoate) (1g): Glycerol and pivalyl chloride were used. Colorless liquid, 66% yield. ^{1}H NMR (500 MHz, $CDCl_3$) δ 5.32 – 5.25 (m, 1H), 4.33 (dd, *J* = 11.9, 4.0 Hz, 2H), 4.13 (dd, *J* = 11.9, 6.1 Hz, 2H), 1.20 (s, 27H). ^{13}C NMR (126 MHz, $CDCl_3$) δ 177.9, 177.4, 69.0, 62.3, 38.78, 38.77, 27.1, 27.0. HR-MS (ESI-TOF) calcd for $C_{18}H_{36}NO_6^+$[M+NH$_4$]$^+$: 362.2537, found 362.2527.

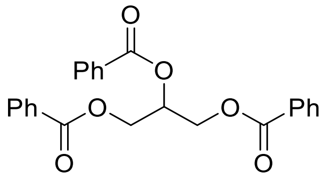

Propane-1,2,3-triyl tribenzoate[4] (1h): Glycerol and benzoyl chloride were used. Colorless liquid, 70% yield. ^{1}H NMR (500 MHz, $CDCl_3$) δ 8.08 – 8.00 (m, 6H), 7.56 (t, *J* = 7.4 Hz, 3H), 7.47 – 7.39 (m, 6H), 5.86 – 5.81 (m, 1H), 4.75 (dd, *J* = 12.0, 4.3 Hz,

2H), 4.69 (dd, J = 11.9, 5.8 Hz, 2H).

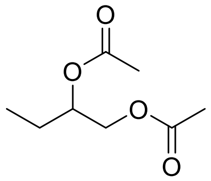

Butane-1,2-diyl diacetate[5] (1l): 1,2-Butaneiol and acetic anhydride were used. Colorless liquid, 86% yield. ^{1}H NMR (500 MHz, $CDCl_3$) δ 5.04 – 4.99 (m, 1H), 4.23 (dd, J = 11.9, 3.2 Hz, 1H), 4.05 (dd, J = 11.9, 6.6 Hz, 1H), 2.08 (s, 3H), 2.07 (s, 3H), 1.67 – 1.57 (m, 2H), 0.93 (t, J = 7.5 Hz, 3H).

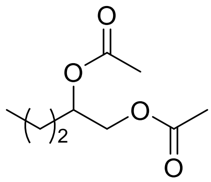

Pentane-1,2-diyl diacetate (1m): 1,2-Pentanediol and acetic anhydride were used. Colorless liquid, 76% yield. ^{1}H NMR (500 MHz, $CDCl_3$) δ 5.09 (s, 1H), 4.23 (d, J = 11.8 Hz, 1H), 4.03 (dd, J = 11.8, 6.5 Hz, 1H), 2.07 (s, 6H), 1.64 – 1.49 (m, 2H), 1.42 – 1.29 (m, 2H), 0.93 (t, J = 7.2 Hz, 3H). ^{13}C NMR (126 MHz, $CDCl_3$) δ 170.8, 170.6, 71.3, 65.1, 32.7, 21.0, 20.8, 18.4, 13.8. HR-MS (ESI-TOF) calcd for $C_9H_{20}NO_4^+[M+NH_4]^+$: 206.1387, found 206.1383.

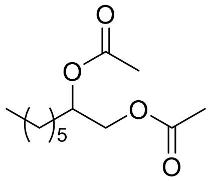

Octane-1,2-diyl diacetate[5] (1n): 1,2-Octanediol and acetic anhydride were used. Colorless liquid, 76% yield. ^{1}H NMR (500 MHz, $CDCl_3$) δ 5.10 – 5.04 (m, 1H), 4.23 (dd, J = 11.9, 3.3 Hz, 1H), 4.03 (dd, J = 11.9, 6.6 Hz, 1H), 2.07 (s, 3H), 2.06 (s, 3H), 1.62 – 1.52 (m, 2H), 1.37 – 1.22 (m, 8H), 0.88 (t, J = 6.9 Hz, 3H).

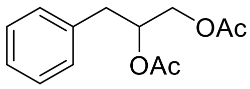

3-Phenylpropane-1,2-diyl diacetate[6] (1o): Synthesized by diacetoxylation of allylbenzene using $PhI(OAc)_2$ as oxidant.[6] Colorless liquid, 60% yield. ^{1}H NMR (500 MHz, $CDCl_3$) δ 7.32 (t, J = 7.4 Hz, 2H), 7.26 (d, J = 7.2 Hz, 1H), 7.23 (d, J = 7.3 Hz, 2H), 5.36 – 5.21 (m, J = 6.7, 3.3 Hz, 1H), 4.25 (dd, J = 12.0, 3.3 Hz, 1H), 4.04 (dd, J = 12.0, 6.2 Hz, 1H), 2.96 (dd, J = 13.8, 7.0 Hz, 1H), 2.90 (dd, J = 13.8, 6.9 Hz, 1H), 2.10 (s, 3H), 2.05 (s, 3H).

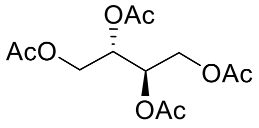

(2*R*,3*S*)-butane-1,2,3,4-tetrayl tetraacetate[7] (1p): Meso-erythritol and acetic anhydride were used. White solid, 76% yield. ^{1}H NMR (500 MHz, $CDCl_3$) δ 5.27 (s, 2H), 4.33 (d, *J* = 12.3 Hz, 2H), 4.20 (dd, *J* = 12.2, 2.1 Hz, 2H), 2.10 (s, 6H), 2.08 (s, 6H).

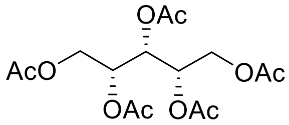

(2*R*,3*r*,4*S*)-Pentane-1,2,3,4,5-pentayl pentaacetate[8] (1q): Xylitol and acetic anhydride were used. White solid, 60% yield. ^{1}H NMR (500 MHz, $CDCl_3$) δ 5.41 (t, *J* = 5.3 Hz, 1H), 5.29 (dd, *J* = 10.3, 5.4 Hz, 2H), 4.35 (dd, *J* = 12.0, 4.3 Hz, 2H), 3.99 (dd, *J* = 12.0, 6.1 Hz, 2H), 2.12 (s, 3H), 2.11 (s, 6H), 2.07 (s, 6H).

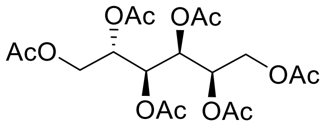

(2*R*,3*S*,4*S*,5*S*)-Hexane-1,2,3,4,5,6-hexayl hexaacetate[5] (1r): Sorbitol and acetic anhydride were used. White solid, 88% yield. ^{1}H NMR (500 MHz, $CDCl_3$) δ 5.47 – 5.39 (m, 2H), 5.25 (dd, *J* = 10.1, 5.9 Hz, 1H), 5.05 (dd, *J* = 9.7, 5.5 Hz, 1H), 4.38 (dd, *J* = 12.1, 4.0 Hz, 1H), 4.25 (dd, *J* = 12.4, 3.5 Hz, 1H), 4.13 (dd, *J* = 12.4, 5.3 Hz, 1H), 4.03 (dd, *J* = 12.1, 6.1 Hz, 1H), 2.14 (s, 3H), 2.10 (s, 3H), 2.09 (s, 3H), 2.08 (s, 3H), 2.07 (s, 3H), 2.06 (s, 3H).

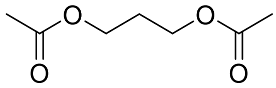

Propane-1,3-diyl diacetate[9] (1s): 1,3-Propanediol and acetic anhydride were used. Colorless liquid, 62% yield. ^{1}H NMR (500 MHz, $CDCl_3$) δ 4.15 (t, *J* = 6.3 Hz, 4H), 2.06 (s, 6H), 2.01 – 1.95 (m, *J* = 6.3 Hz, 2H).

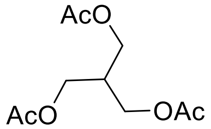

2-(Acetoxymethyl)propane-1,3-diyl diacetate (1t): 2-(Hydroxymethyl)propane-1,3-diol and acetic anhydride were used. Colorless liquid, 86% yield. ^{1}H NMR (500 MHz, $CDCl_3$) δ 4.14 (d, *J* = 6.0 Hz, 6H), 2.43 – 2.37 (m, 1H), 2.07 (s, 9H). ^{13}C NMR (126

MHz, $CDCl_3$) δ 170.9, 61.8, 37.2, 20.8. HR-MS (ESI-TOF) calcd for $C_{10}H_{20}NO_6^+[M+NH_4]^+$: 250.1285, found 250.1280.

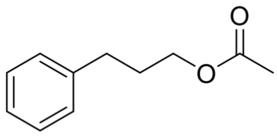

3-Phenylpropyl acetate[10] (1w): 3-Phenyl-1-propanol and acetic anhydride were used. Colorless liquid, 85% yield. ^{1}H NMR (500 MHz, $CDCl_3$) δ 7.32 (t, *J* = 7.6 Hz, 2H), 7.25 – 7.19 (m, 3H), 4.12 (t, *J* = 6.6 Hz, 2H), 2.72 (t, *J* = 7.6 Hz, 2H), 2.09 (s, 3H), 2.03 – 1.95 (m, 2H).

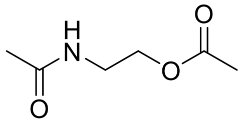

2-Acetamidoethyl acetate[11] (4a): Ethanolamine and acetic anhydride were used. Colorless liquid, 56% yield. ^{1}H NMR (500 MHz, $CDCl_3$) δ 6.11 (s, 1H), 4.16 (t, *J* = 5.4 Hz, 2H), 3.51 (dd, *J* = 10.9, 5.5 Hz, 2H), 2.09 (s, 3H), 2.00 (s, 3H).

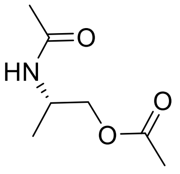

(*S*)-2-Acetamidopropyl acetate[12] (4b): L-alaninol and acetic anhydride were used. White solid, 72% yield. ^{1}H NMR (500 MHz, $CDCl_3$) δ 5.73 (s, 1H), 4.33 – 4.24 (m, 1H), 4.11 (dd, *J* = 11.2, 5.7 Hz, 1H), 4.01 (dd, *J* = 11.2, 4.3 Hz, 1H), 2.09 (s, 3H), 1.98 (s, 3H), 1.18 (d, *J* = 6.8 Hz, 3H).

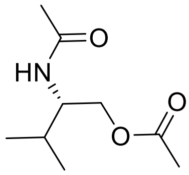

(*S*)-2-Acetamido-3-methylbutyl acetate (4c): L-valinol and acetic anhydride were used. White solid, 66% yield, mp 54-55 °C. ^{1}H NMR (500 MHz, $CDCl_3$) δ 5.72 (d, *J* = 8.0 Hz, 1H), 4.20 (dd, *J* = 11.2, 6.1 Hz, 1H), 4.08 – 3.99 (m, 2H), 2.07 (s, 3H), 2.01 (s, 3H), 1.86 – 1.78 (m, 1H), 0.95 (t, *J* = 6.4 Hz, 6H). ^{13}C NMR (126 MHz, $CDCl_3$) δ 171.1, 170.0, 64.5, 53.3, 29.4, 23.3, 20.8, 19.2, 18.5. HR-MS (ESI-TOF) calcd for $C_9H_{18}NO_3^+[M+H]^+$: 188.1281, found 188.1277. $[\alpha]_D^{20}$ = -68.9 (c = 1.0, $CHCl_3$).

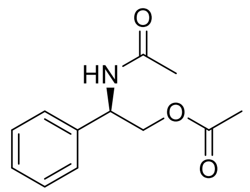

(*R*)-2-Acetamido-2-phenylethyl acetate[13] (4d): (*R*)-phenylglycinol and acetic anhydride were used. White solid, 82% yield. ^{1}H NMR (500 MHz, $CDCl_3$) δ 7.38 – 7.28 (m, 5H), 6.45 – 6.35 (m, 1H), 5.29 (dd, *J* = 12.5, 7.5 Hz, 1H), 4.44 – 4.37 (m, 1H), 4.26 (dd, *J* = 11.5, 4.8 Hz, 1H), 2.05 (s, 3H), 2.01 (s, 3H).

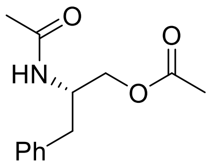

(*S*)-2-Acetamido-3-phenylpropyl acetate (4e): L-phenylalaninol and acetic anhydride were used. White solid, 72% yield, mp 126-127 °C. ^{1}H NMR (500 MHz, $CDCl_3$) δ 7.31 (t, *J* = 7.4 Hz, 2H), 7.24 (dd, *J* = 8.4, 6.3 Hz, 1H), 7.19 (d, *J* = 7.0 Hz, 2H), 5.65 (d, *J* = 8.0 Hz, 1H), 4.48 – 4.39 (m, 1H), 4.08 (dd, *J* = 11.4, 5.4 Hz, 1H), 4.03 (dd, *J* = 11.4, 4.3 Hz, 1H), 2.89 (dd, *J* = 13.8, 6.3 Hz, 1H), 2.81 (dd, *J* = 13.8, 7.8 Hz, 1H), 2.10 (s, 3H), 1.96 (s, 3H). ^{13}C NMR (126 MHz, $CDCl_3$) δ 171.0, 169.7, 136.9, 129.1, 128.6, 126.8, 64.7, 49.4, 37.4, 23.3, 20.8. HR-MS (ESI-TOF) calcd for $C_{13}H_{18}NO_3^+$[M+H]$^+$: 236.1281, found 236.1274. $[\alpha]_D^{20}$ = -15.5 (c = 1.0, $CHCl_3$).

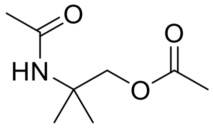

2-Acetamido-2-methylpropyl acetate (4f): 2-Amino-2-methyl-1-propanol and acetic anhydride were used. White solid, 72% yield, mp 53-55 °C. ^{1}H NMR (500 MHz, $CDCl_3$) δ 5.71 (s, 1H), 4.19 (s, 2H), 2.09 (s, 1H), 1.94 (s, 3H), 1.35 (s, 6H). ^{13}C NMR (126 MHz, $CDCl_3$) δ 171.0, 169.9, 68.9, 53.1, 24.1, 23.8, 20.8. HR-MS (ESI-TOF) calcd for $C_8H_{16}NO_3^+$[M+H]$^+$: 174.1125, found 174.1121.

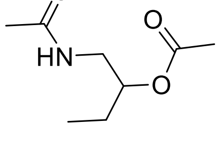

1-Acetamidobutan-2-yl acetate (4g): 2-Hydroxybutylamine was obtained by amination of ethyloxirane by simple extraction of crude product,[14] then reacted with acetic anhydride. Colorless liquid, 42% yield for two steps. ^{1}H NMR (500 MHz, $CDCl_3$) δ 6.00 (s, 1H), 4.90 – 4.81 (m, 1H), 3.51 – 3.42 (m, 1H), 3.42 – 3.34 (m, 1H), 2.09 (s,

3H), 1.98 (s, 3H), 1.64 – 1.57 (m, 2H), 0.93 (t, *J* = 7.5 Hz, 3H). ^{13}C NMR (126 MHz, $CDCl_3$) δ 171.3, 170.3, 74.6, 42.6, 24.8, 23.1, 21.1, 9.5. HR-MS (ESI-TOF) calcd for $C_8H_{16}NO_3^+$[M+H]$^+$: 174.1125, found 174.1120.

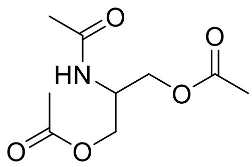

2-Acetamidopropane-1,3-diyl diacetate (4h): Serinol and acetic anhydride were used. White solid, 56% yield, mp 81-82 °C. ^{1}H NMR (500 MHz, $CDCl_3$) δ 5.95 (d, *J* = 7.8 Hz, 1H), 4.51 – 4.40 (m, 1H), 4.23 (dd, *J* = 11.4, 5.3 Hz, 2H), 4.09 (dd, *J* = 11.4, 5.3 Hz, 2H), 2.09 (s, 6H), 2.01 (s, 3H). ^{13}C NMR (126 MHz, $CDCl_3$) δ 170.8, 169.9, 62.8, 47.3, 23.2, 20.7. HR-MS (ESI-TOF) calcd for $C_9H_{16}NO_5^+$[M+H]$^+$: 218.1023, found 218.1016.

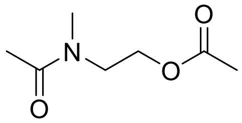

2-(*N*-methylacetamido)ethyl acetate (4i): 2-Methylaminoethanol and acetic anhydride were used. Colorless liquid, 65% yield. Mixture of stereoisomers about the amide bond in ratio of 3:2. Major: ^{1}H NMR (500 MHz, $CDCl_3$) δ 4.22 (t, *J* = 5.4 Hz, 2H), 3.62 (t, *J* = 5.6 Hz, 2H), 3.07 (s, 3H), 2.10 (s, 3H), 2.07 (s, 3H). ^{13}C NMR (126 MHz, $CDCl_3$) δ 170.9, 170.8, 62.2, 46.6, 37.3, 21.7, 20.8. Minor: ^{1}H NMR (500 MHz, $CDCl_3$) δ 4.21 (t, *J* = 5.7 Hz, 2H), 3.57 (t, *J* = 5.7 Hz, 3H), 2.96 (s, 3H), 2.13 (s, 3H), 2.08 (s, 3H). ^{13}C NMR (126 MHz, $CDCl_3$) δ 170.8, 170.6, 61.2, 49.1, 33.4, 21.2, 20.7. HR-MS (ESI-TOF) calcd for $C_7H_{14}NO_3^+$[M+H]$^+$: 160.0968, found 160.0964.

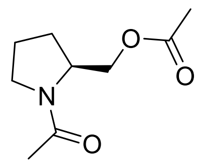

(*S*)-(1-Acetylpyrrolidin-2-yl)methyl acetate[15] (4j): L-prolinol and acetic anhydride were used. White solid, 68% yield. Mixture of stereoisomers about the amide bond in ratio of 2:1. ^{1}H NMR (500 MHz, $CDCl_3$) δ 4.48 – 4.40 (m, 0.6H), 4.31 – 4.15 (m, 2H), 4.03 – 3.95 (m, 0.3H), 3.70 – 3.45 (m, 2H), 2.28 – 1.90 (m, 10H).

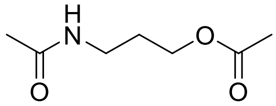

3-Acetamidopropyl acetate (4k): 3-Amino-1-propanol and acetic anhydride were used. Colorless liquid, 60% yield. ^{1}H NMR (500 MHz, $CDCl_3$) δ 5.88 (s, 1H), 4.14 (t,

J = 6.2 Hz, 2H), 3.31 (q, *J* = 6.4 Hz, 2H), 2.07 (s, 31H), 1.99 (s, 3H), 1.88 – 1.81 (m, 2H). ^{13}C NMR (126 MHz, $CDCl_3$) δ 171.3, 170.3, 61.8, 36.2, 28.6, 23.2, 20.9. HR-MS (ESI-TOF) calcd for $C_7H_{14}NO_3^+$[M+H]$^+$: 160.0968, found 160.0965.

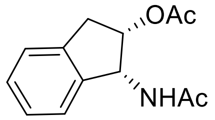

(1*R*,2*S*)-1-Acetamido-2,3-dihydro-1*H*-inden-2-yl acetate (4l): (1*R*,2*S*)-1-Amino-2-indanol and acetic anhydride were used. White solid, 73% yield, mp 176-177 °C. ^{1}H NMR (500 MHz, $CDCl_3$) δ 7.30 – 7.22 (m, 4H), 5.91 (d, *J* = 8.5 Hz, 1H), 5.69 (dd, *J* = 9.2, 5.4 Hz, 1H), 5.56 (t, *J* = 4.7 Hz, 1H), 3.22 (dd, *J* = 17.2, 5.1 Hz, 1H), 3.02 (d, *J* = 17.1 Hz, 1H), 2.10 (s, 3H), 2.03 (s, 3H). ^{13}C NMR (126 MHz, $CDCl_3$) δ 170.2, 170.0, 140.5, 139.3, 128.3, 127.2, 125.0, 123.7, 75.8, 55.3, 37.5, 23.2, 21.0. HR-MS (ESI-TOF) calcd for $C_{13}H_{16}NO_3^+$[M+H]$^+$: 234.1125, found 234.1118. $[\alpha]_D^{20}$ = 83.8 (c = 1.0, $CHCl_3$).

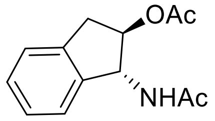

(1*R*,2*R*)-1-Acetamido-2,3-dihydro-1*H*-inden-2-yl acetate (4l'): (1*R*,2*R*)-1-Aminoindan-2-ol and acetic anhydride were used. White solid, 75% yield, mp 146-148 °C. ^{1}H NMR (500 MHz, $CDCl_3$) δ 7.30 – 7.22 (m, 3H), 7.20 (d, *J* = 6.7 Hz, 1H), 5.90 (d, *J* = 7.8 Hz, 1H), 5.55 – 5.49 (m, 1H), 5.30 (q, *J* = 6.9 Hz, 1H), 3.35 (dd, *J* = 16.1, 7.5 Hz, 1H), 2.92 (dd, *J* = 16.0, 6.9 Hz, 1H), 2.10 (s, 3H), 2.05 (s, 3H). ^{13}C NMR (126 MHz, $CDCl_3$) δ 171.2, 170.3, 139.8, 138.8, 128.6, 127.5, 124.8, 124.1, 79.9, 59.1, 36.2, 23.2, 21.1. HR-MS (ESI-TOF) calcd for $C_{13}H_{16}NO_3^+$[M+H]$^+$: 234.1125, found 234.1118. $[\alpha]_D^{20}$ = -103.2 (c = 1.0, $CHCl_3$).

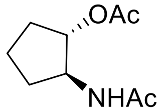

(1*S*,2*S*)-2-Acetamidocyclopentyl acetate[16] (4m): (1*S*,2*S*)-2-Aminocyclopentanol hydrochloride and acetic anhydride were used. White solid, 65% yield. ^{1}H NMR (500 MHz, $CDCl_3$) δ 5.87 (s, 1H), 4.97 (dd, *J* = 13.6, 6.6 Hz, 1H), 4.20 – 4.06 (m, 1H), 2.30 – 2.21 (m, 1H), 2.11 – 1.99 (m, 4H), 1.96 (s, 3H), 1.86 – 1.64 (m, 3H), 1.47 – 1.33 (m, 1H).

Standard procedure for the mono-amidation of polyol esters with sulfonamides

Polyol ester (1.0 mmol), sulfonamide (0.5 mmol) and $Hf(OTf)_4$ (0.01 mmol) were

added to a 5 mL sample vial equipped with a magnetic stir bar. The vial was sealed and stirred at 120 °C for 24 hours. After completion by TLC, the mixture was cooled down to room temperature and purified by column chromatography (PE : EA = 4 : 1) to afford the target product.

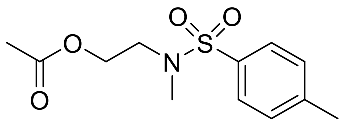

2-((*N*,4-Dimethylphenyl)sulfonamido)ethyl acetate (3aa): The standard procedure was followed. Colorless oil, 90% yield. ^{1}H NMR (500 MHz, $CDCl_3$) δ 7.67 (d, *J* = 8.1 Hz, 2H), 7.32 (d, *J* = 8.0 Hz, 2H), 4.21 (t, *J* = 5.7 Hz, 2H), 3.28 (t, *J* = 5.7 Hz, 2H), 2.81 (s, 3H), 2.43 (s, 3H), 2.06 (s, 3H). ^{13}C NMR (126 MHz, $CDCl_3$) δ 170.8, 143.5, 134.6, 129.7, 127.3, 61.8, 48.8, 35.7, 21.5, 20.8. HR-MS (ESI-TOF) calcd for $C_{12}H_{18}NO_4S^+[M+H]^+$: 272.0951, found 272.0949.

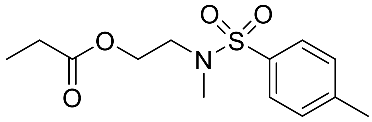

2-((*N*,4-Dimethylphenyl)sulfonamido)ethyl propionate (3ba): The standard procedure was followed. Colorless oil, 83% yield. ^{1}H NMR (500 MHz, $CDCl_3$) δ 7.67 (d, *J* = 8.2 Hz, 2H), 7.32 (d, *J* = 8.0 Hz, 2H), 4.22 (t, *J* = 5.7 Hz, 2H), 3.28 (t, *J* = 5.7 Hz, 2H), 2.81 (s, 3H), 2.43 (s, 3H), 2.33 (q, *J* = 7.6 Hz, 2H), 1.13 (t, *J* = 7.5 Hz, 3H). ^{13}C NMR (126 MHz, $CDCl_3$) δ 174.2, 143.5, 134.6, 129.7, 127.3, 61.7, 48.8, 35.7, 27.4, 21.5, 9.0. HR-MS (ESI-TOF) calcd for $C_{13}H_{20}NO_4S^+[M+H]^+$: 286.1108, found 286.1105.

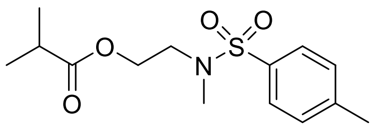

2-((*N*,4-Dimethylphenyl)sulfonamido)ethyl isobutyrate (3ca): The standard procedure was followed. Colorless oil, 84% yield. ^{1}H NMR (500 MHz, $CDCl_3$) δ 7.69 (d, *J* = 8.0 Hz, 2H), 7.34 (d, *J* = 7.9 Hz, 2H), 4.23 (t, *J* = 5.6 Hz, 2H), 3.29 (t, *J* = 5.5 Hz, 2H), 2.83 (s, 3H), 2.59 – 2.53 (m, 1H), 2.44 (s, 3H), 1.18 (d, *J* = 7.0 Hz, 6H). ^{13}C NMR (126 MHz, $CDCl_3$) δ 176.8, 143.5, 134.6, 129.7, 127.3, 61.8, 48.9, 35.8, 33.9, 21.5, 18.9. HR-MS (ESI-TOF) calcd for $C_{14}H_{22}NO_4S^+[M+H]^+$: 300.1264, found 300.1265.

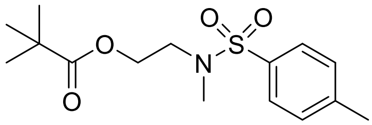

2-((*N*,4-Dimethylphenyl)sulfonamido)ethyl pivalate (3da): The standard procedure was followed. Colorless oil, 83% yield. ^{1}H NMR (500 MHz, $CDCl_3$) δ 7.67 (d, *J* = 8.2 Hz, 2H), 7.33 (d, *J* = 8.0 Hz, 2H), 4.22 (t, *J* = 5.6 Hz, 2H), 3.28 (t, *J* = 5.6 Hz, 2H), 2.82 (s, 3H), 2.43 (s, 3H), 1.20 (s, 9H). ^{13}C NMR (126 MHz, $CDCl_3$) δ 178.3, 143.5, 134.6, 129.8, 127.3, 62.1, 48.9, 38.7, 35.9, 27.1, 21.5. HR-MS (ESI-TOF) calcd for $C_{15}H_{24}NO_4S^+$[M+H]$^+$: 314.1421, found 314.1419.

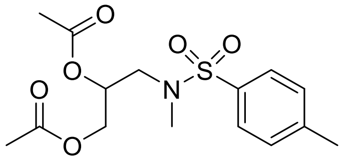

3-((*N*,4-Dimethylphenyl)sulfonamido)propane-1,2-diyl diacetate (3ea): 5 mol% of $Hf(OTf)_4$ was used. Colorless oil, 42% yield. ^{1}H NMR (500 MHz, $CDCl_3$) δ 7.67 (d, *J* = 8.2 Hz, 2H), 7.34 (d, *J* = 8.1 Hz, 2H), 5.27 – 5.17 (m, 1H), 4.39 (dd, *J* = 12.2, 3.4 Hz, 1H), 4.18 (dd, *J* = 12.2, 5.9 Hz, 1H), 3.20 (d, *J* = 6.1Hz, 2H), 2.80 (s, 3H), 2.44 (s, 3H), 2.09 (s, 3H), 2.08 (s, 3H). ^{13}C NMR (126 MHz, $CDCl_3$) δ 170.6, 170.2, 143.7, 134.1, 129.8, 127.4, 69.0, 62.9, 50.0, 36.2, 21.5, 20.9, 20.7. HR-MS (ESI-TOF) calcd for $C_{15}H_{22}NO_6S^+$[M+H]$^+$: 344.1162, found 344.1158.

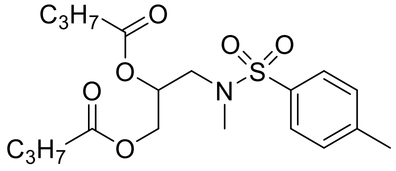

3-((*N*,4-Dimethylphenyl)sulfonamido)propane-1,2-diyl dibutyrate (3fa): 5 mol% of $Hf(OTf)_4$ was used. Colorless oil, 48% yield. ^{1}H NMR (500 MHz, $CDCl_3$) δ 7.66 (d, *J* = 7.5 Hz, 2H), 7.33 (d, *J* = 7.7 Hz, 2H), 5.24 (s, 1H), 4.40 (d, *J* = 12.1 Hz, 1H), 4.17 (dd, *J* = 12.1, 5.9 Hz, 1H), 3.22 (dd, *J* = 13.8, 6.5 Hz, 1H), 3.15 (dd, *J* = 13.9, 5.3 Hz, 1H), 2.79 (s, 3H), 2.44 (s, 3H), 2.30 (q, *J* = 7.6 Hz, 4H), 1.69 – 1.61 (m, 4H), 1.00 – 0.91 (m, 6H). ^{13}C NMR (126 MHz, $CDCl_3$) δ 173.1, 172.8, 143.6, 134.1, 129.7, 127.3, 68.7, 62.7, 50.1, 36.2, 36.0, 35.9, 21.5, 18.3, 18.2, 13.6, 13.5. HR-MS (ESI-TOF) calcd for $C_{19}H_{30}NO_6S^+$[M+H]$^+$: 400.1788, found 400.1789.

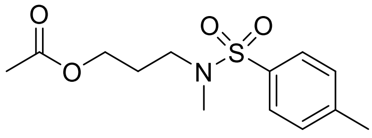

3-((*N*,4-Dimethylphenyl)sulfonamido)propyl acetate (3sa): The standard procedure

was followed. Colorless oil, 79% yield. ^{1}H NMR (500 MHz, $CDCl_3$) δ 7.67 (d, J = 8.2 Hz, 2H), 7.33 (d, J = 8.0 Hz, 2H), 4.13 (t, J = 6.3 Hz, 2H), 3.09 (t, J = 7.0 Hz, 2H), 2.73 (s, 3H), 2.44 (s, 3H), 2.06 (s, 3H), 1.92 – 1.84 (m, 2H). ^{13}C NMR (126 MHz, $CDCl_3$) δ 170.9, 1434, 134.2, 129.6, 127.3, 61.5, 47.0, 34.9, 26.9, 21.4, 20.8. HR-MS (ESI-TOF) calcd for $C_{13}H_{20}NO_4S^+$[M+H]$^+$: 286.1108, found 286.1107.

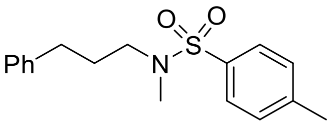

***N*,4-Dimethyl-*N*-(3-phenylpropyl)benzenesulfonamide (3wa):** The standard procedure was followed. Colorless oil, 18% yield. ^{1}H NMR (500 MHz, $CDCl_3$) δ 7.65 (d, J = 8.2 Hz, 2H), 7.33 – 7.26 (m, 4H), 7.21 – 7.16 (m, 3H), 3.03 (t, J = 7.1 Hz, 2H), 2.71 (s, 3H), 2.66 (t, J = 7.1 Hz, 2H), 2.42 (s, 3H), 1.88 – 1.81 (m, 2H). ^{13}C NMR (126 MHz, $CDCl_3$) δ 143.3, 141.4, 134.4, 129.7, 128.5, 128.4, 127.5, 126.0, 49.8, 34.7, 32.8, 29.4, 21.5. HR-MS (ESI-TOF) calcd for $C_{17}H_{22}NO_2S^+$[M+H]$^+$: 304.1366, found 304.1365.

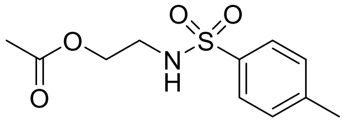

2-((4-Methylphenyl)sulfonamido)ethyl acetate (3ab): Reaction time: 14 hours. Colorless oil, 85% yield. ^{1}H NMR (500 MHz, $CDCl_3$) δ 7.76 (d, J = 8.3 Hz, 2H), 7.32 (d, J = 8.0 Hz, 2H), 4.92 (t, J = 6.1 Hz, 1H), 4.09 (t, J = 5.3 Hz, 2H), 3.22 (dd, J = 10.8, 6.0 Hz, 2H), 2.44 (s, 3H), 2.01 (s, 3H). ^{13}C NMR (126 MHz, $CDCl_3$) δ 170.8, 143.7, 136.8, 129.8, 127.0, 62.9, 42.1, 21.5, 20.7. HR-MS (ESI-TOF) calcd for $C_{11}H_{16}NO_4S^+$[M+H]$^+$: 258.0795, found 258.0792.

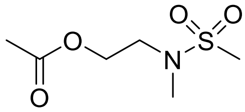

2-(*N*-Methylmethylsulfonamido)ethyl acetate (3af): The standard procedure was followed. Colorless oil, 92% yield. ^{1}H NMR (500 MHz, $CDCl_3$) δ 4.24 (t, J = 5.5 Hz, 2H), 3.44 (t, J = 5.5 Hz, 2H), 2.95 (s, 3H), 2.86 (s, 3H), 2.09 (s, 3H). ^{13}C NMR (126 MHz, $CDCl_3$) δ 170.7, 61.8, 48.6, 36.5, 35.2, 20.8. HR-MS (ESI-TOF) calcd for $C_6H_{14}NO_4S^+$[M+H]$^+$: 196.0638, found 196.0637.

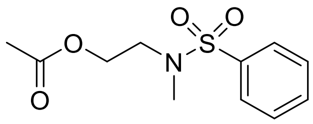

2-(*N*-methylphenylsulfonamido)ethyl acetate (3ag): The standard procedure was followed. White solid, 94% yield, mp 75-76 °C. ^{1}H NMR (500 MHz, $CDCl_3$) δ 7.81 (d, *J* = 7.4 Hz, 2H), 7.61 (t, *J* = 7.4 Hz, 1H), 7.55 (t, *J* = 7.5 Hz, 2H), 4.23 (t, *J* = 5.6 Hz, 2H), 3.32 (t, *J* = 5.6 Hz, 2H), 2.85 (s, 3H), 2.06 (s, 3H). ^{13}C NMR (126 MHz, $CDCl_3$) δ 170.7, 137.7, 132.7, 129.1, 127.2, 61.7, 48.8, 35.7, 20.8. HR-MS (ESI-TOF) calcd for $C_{11}H_{16}NO_4S^+[M+H]^+$: 258.0795, found 258.0794.

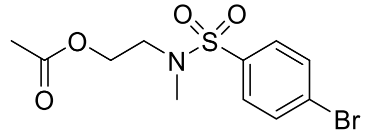

2-((4-Bromo-*N*-methylphenyl)sulfonamido)ethyl acetate (3ah): The standard procedure was followed. White solid, 95% yield, mp 86-87 °C. ^{1}H NMR (500 MHz, $CDCl_3$) δ 7.70 – 7.64 (m, 4H), 4.22 (t, *J* = 5.6 Hz, 2H), 3.30 (t, *J* = 5.6 Hz, 2H), 2.84 (s, 3H), 2.06 (s, 3H). ^{13}C NMR (126 MHz, $CDCl_3$) δ 170.7, 136.8, 132.4, 128.7, 127.7, 61.6, 48.7, 35.6, 20.8. HR-MS (ESI-TOF) calcd for $C_{11}H_{15}BrNO_4S^+[M+H]^+$: 335.9900, found 335.9900.

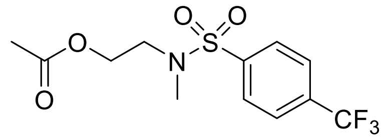

2-((*N*-Methyl-4-(trifluoromethyl)phenyl)sulfonamido)ethyl acetate (3ai): The standard procedure was followed. White solid, 98% yield, mp 57-58 °C. ^{1}H NMR (500 MHz, $CDCl_3$) δ 7.93 (d, *J* = 8.2 Hz, 2H), 7.81 (d, *J* = 8.3 Hz, 2H), 4.23 (t, *J* = 5.6 Hz, 2H), 3.35 (t, *J* = 5.6 Hz, 2H), 2.88 (s, 3H), 2.05 (s, 3H). ^{13}C NMR (126 MHz, $CDCl_3$) δ 170.7, 141.5, 134.4 (q, *J* = 33.1 Hz), 127.7, 126.3 (q, *J* = 3.7 Hz), 124.2, 122.1, 61.5, 48.8, 35.6, 20.7. HR-MS (ESI-TOF) calcd for $C_{12}H_{15}F_3NO_4S^+[M+H]^+$: 326.0668, found 326.0667.

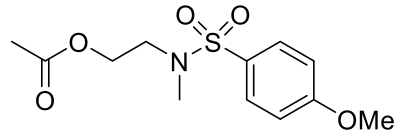

2-((4-Methoxy-*N*-methylphenyl)sulfonamido)ethyl acetate (3aj): The standard procedure was followed. White solid, 91% yield, mp 77-79 °C. ^{1}H NMR (500 MHz, $CDCl_3$) δ 7.73 (d, *J* = 8.9 Hz, 2H), 7.00 (d, *J* = 8.9 Hz, 2H), 4.21 (t, *J* = 5.7 Hz, 2H), 3.88 (s, 3H), 3.27 (t, *J* = 5.7 Hz, 2H), 2.81 (s, 3H), 2.06 (s, 3H). ^{13}C NMR (126 MHz, $CDCl_3$) δ 170.8, 162.9, 129.4, 129.2, 114.2, 61.8, 55.6, 48.7, 35.7, 20.8. HR-MS (ESI-TOF) calcd for $C_{12}H_{18}NO_5S^+[M+H]^+$: 288.0900, found 288.0896.

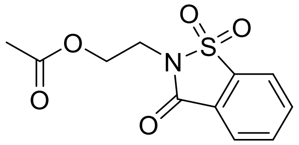

2-(1,1-Dioxido-3-oxobenzo[*d*]isothiazol-2(3*H*)-yl)ethyl acetate (3am): The standard procedure was followed. White solid, 98% yield, mp 86-88 °C. ^{1}H NMR (500 MHz, $CDCl_3$) δ 8.08 (dd, *J* = 7.8, 1.3 Hz, 1H), 7.94 (dd, *J* = 7.2, 1.3 Hz, 1H), 7.90 (td, *J* = 7.5, 1.3 Hz, 1H), 7.85 (td, *J* = 7.4, 1.3 Hz, 1H), 4.43 (t, *J* = 5.3 Hz, 2H), 4.03 (t, *J* = 5.3 Hz, 2H), 2.08 (s, 3H). ^{13}C NMR (126 MHz, $CDCl_3$) δ 170.9, 159.1, 137.6, 135.0, 134.5, 127.1, 125.3, 121.1, 60.8, 38.4, 20.9. HR-MS (ESI-TOF) calcd for $C_{11}H_{12}F_3NO_5S^+$[M+H]$^+$: 270.0431, found 270.0431.

Standard procedure for the mono-amidation of polyol ester with saccharin

Polyol ester (1.0 mmol), saccharin (0.5 mmol) and $Sc(OTf)_3$ (0.01 mmol) were added to a 5 ml sample vial equipped with a magnetic stir bar. The vial was sealed and stirred at 150 °C for 24 hours. After completion by TLC, the mixture was cooled down to room temperature and purified by column chromatography (PE : EA = 3 : 1) to afford the target product.

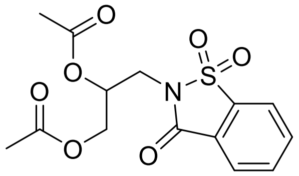

3-(1,1-Dioxido-3-oxobenzo[*d*]isothiazol-2(3*H*)-yl)propane-1,2-diyl diacetate (3em): The standard procedure was followed. White solid, 86% yield, mp 100-101 °C. ^{1}H NMR (500 MHz, $CDCl_3$) δ 8.07 (d, *J* = 7.5 Hz, 1H), 7.93 (d, *J* = 7.5 Hz, 1H), 7.89 (t, *J* = 7.4 Hz, 1H), 7.85 (t, *J* = 7.4 Hz, 1H), 5.49 – 5.41 (m, 1H), 4.35 (dd, *J* = 12.1, 4.1 Hz, 1H), 4.23 (dd, *J* = 12.1, 5.0 Hz, 1H), 4.04 (dd, *J* = 15.2, 3.7 Hz, 1H), 3.95 (dd, *J* = 15.2, 7.4 Hz, 1H), 2.12 (s, 3H), 2.09 (s, 3H). ^{13}C NMR (126 MHz, $CDCl_3$) δ 170.4, 170.3, 159.3, 137.5, 135.0, 134.4, 126.8, 125.3, 121.0, 68.2, 62.7, 39.7, 20.9, 20.7. HR-MS (ESI-TOF) calcd for $C_{14}H_{19}N_2O_7S^+$ [M+NH$_4$]$^+$: 359.0907, found359.0904.

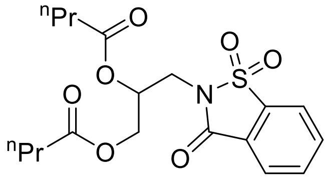

3-(1,1-Dioxido-3-oxobenzo[*d*]isothiazol-2(3*H*)-yl)propane-1,2-diyl dibutyrate (3fm): The standard procedure was followed. Colorless oil, 85% yield. ^{1}H NMR (500

MHz, $CDCl_3$) δ 8.07 (d, *J* = 7.4 Hz, 1H), 7.93 (d, *J* = 7.4 Hz, 1H), 7.89 (t, *J* = 7.2 Hz, 1H), 7.85 (t, *J* = 7.5 Hz, 1H), 5.50 – 5.45 (m, 1H), 4.38 (dd, *J* = 12.1, 4.3 Hz, 1H), 4.23 (dd, *J* = 12.1, 5.2 Hz, 1H), 4.04 (dd, *J* = 15.2, 4.1 Hz, 1H), 3.97 (dd, *J* = 15.2, 7.2 Hz, 1H), 2.41 – 2.28 (m, 4H), 1.73 – 1.57 (m, 4H), 0.97 (t, *J* = 7.4 Hz, 3H), 0.91 (t, *J* = 7.4 Hz, 3H). ^{13}C NMR (126 MHz, $CDCl_3$) δ 173.0, 172.8 159.3, 137.6, 135.0, 134.4, 126.8, 125.3, 121.0, 68.1, 62.5, 39.7, 35.9, 35.8, 18.3, 18.0, 13.6, 13.5. HR-MS (ESI-TOF) calcd for $C_{18}H_{27}N_2O_7S^+$ $[M+NH_4]^+$: 415.1533, found 415.1530.

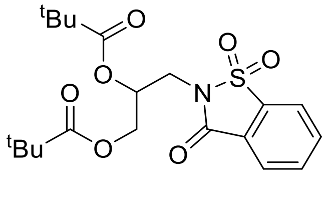

3-(1,1-Dioxido-3-oxobenzo[*d*]isothiazol-2(3*H*)-yl)propane-1,2-diyl bis(2,2-dimethyl-propanoate) (3gm): The standard procedure was followed. Colorless oil, 76% yield. ^1H NMR (500 MHz, $CDCl_3$) δ 8.07 (d, *J* = 7.5 Hz, 1H), 7.96 – 7.82 (m, 3H), 5.49 – 5.42 (m, 1H), 4.42 (dd, *J* = 12.1, 4.1 Hz, 1H), 4.17 (dd, *J* = 12.1, 4.9 Hz, 1H), 4.09 – 3.97 (m, 2H), 1.24 (s, 9H), 1.19 (s, 9H). ^{13}C NMR (126 MHz, $CDCl_3$) δ 177.8, 177.5, 159.2, 137.6, 135.0, 134.4, 126.9, 125.3, 121.1, 68.5, 62.4, 39.3, 38.81, 38.79, 27.1, 26.9. HR-MS (ESI-TOF) calcd for $C_{20}H_{31}N_2O_7S^+$$[M+NH_4]^+$: 443.1846, found 443.1843.

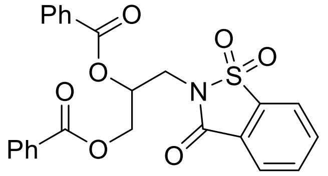

3-(1,1-Dioxido-3-oxobenzo[*d*]isothiazol-2(3*H*)-yl)propane-1,2-diyl dibenzoate (3hm): The standard procedure was followed. White solid, 75% yield, mp 116-117 °C. ^1H NMR (500 MHz, $CDCl_3$) δ 8.12 – 8.03 (m, 5H), 7.92 – 7.79 (m, 3H), 7.56 (dd, *J* = 7.4 Hz, 2H), 7.43 (dd, *J* = 13.5, 7.6 Hz, 4H), 5.90 – 5.84 (m, 1H), 4.73 (dd, *J* = 12.1, 4.4 Hz, 1H), 4.63 (dd, *J* = 12.1, 5.0 Hz, 1H), 4.30 (dd, *J* = 15.3, 7.0 Hz, 1H), 4.24 (dd, *J* = 15.3, 4.3 Hz, 1H). ^{13}C NMR (126 MHz, $CDCl_3$) δ 166.0, 165.7, 159.3, 137.6, 135.0, 134.4, 133.3, 133.2, 130.0, 129.7, 129.41, 129.36, 128.4, 128.3, 126.9, 125.4, 121.1, 69.2, 63.2, 39.8. HR-MS (ESI-TOF) calcd for $C_{24}H_{23}N_2O_7S^+$ $[M+NH_4]^+$: 483.1220, found 483.1221.

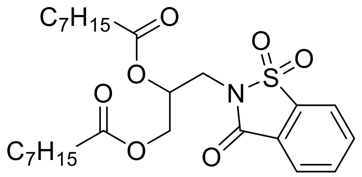

3-(1,1-Dioxido-3-oxobenzo[*d*]isothiazol-2(3*H*)-yl)propane-1,2-diyl dioctanoate (3im): 0.025 mmol of Sc(OTf)$_3$ was used, the mixture was stirred at 180 °C for 24 hours. White solid, 75% yield, mp 46-48 °C. ^{1}H NMR (500 MHz, CDCl$_3$) δ 8.07 (d, *J* = 7.3 Hz, 1H), 7.93 (d, *J* = 7.5 Hz, 1H), 7.89 (t, *J* = 7.4 Hz, 1H), 7.85 (t, *J* = 7.4 Hz, 1H), 4.36 (dd, *J* = 12.1, 4.2 Hz, 1H), 4.22 (dd, *J* = 12.1, 5.0 Hz, 1H), 4.05 – 3.93 (m, 2H), 2.40 – 2.31 (m, 4H), 1.70 – 1.54 (m, 4H), 1.37 – 1.17 (m, 16H), 0.91 – 0.83 (m, 6H). ^{13}C NMR (126 MHz, CDCl$_3$) δ 173.3, 173.0, 159.3, 137.6, 135, 134.4, 126.9, 125.3, 121.0, 68.1, 62.5, 39.8, 34.03, 33.99, 31.61, 31.59, 29.0, 28.93, 28.88, 28.87, 24.8, 24.5, 22.56, 22.54, 14.0. HR-MS (ESI-TOF) calcd for $C_{26}H_{43}N_2O_7S^+$ $[M+NH_4]^+$: 527.2785, found 527.2772.

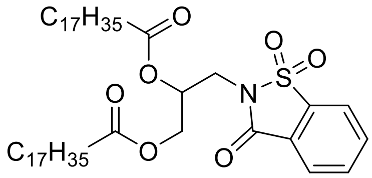

3-(1,1-Dioxido-3-oxobenzo[*d*]isothiazol-2(3*H*)-yl)propane-1,2-diyl distearate (3jm): Reaction time: 48 hours. White solid, 79% yield, mp 82-84 °C. ^{1}H NMR (500 MHz, CDCl$_3$) δ 8.07 (d, *J* = 7.4 Hz, 1H), 7.92 (d, *J* = 7.4 Hz, 1H), 7.88 (t, *J* = 7.3 Hz, 1H), 7.84 (t, *J* = 7.4 Hz, 1H), 5.50 – 5.44 (m, 1H), 4.36 (dd, *J* = 12.1, 4.3 Hz, 1H), 4.21 (dd, *J* = 12.1, 5.1 Hz, 1H), 4.02 (dd, *J* = 15.2, 4.1 Hz, 1H), 3.96 (dd, *J* = 15.2, 7.2 Hz, 1H), 2.40 – 2.30 (m, 4H), 1.68 – 1.54 (m, 4H), 1.35 – 1.18 (m, 56H), 0.88 (t, *J* = 6.8 Hz, 6H). ^{13}C NMR (126 MHz, CDCl$_3$) δ 173.3, 173.1, 159.3, 137.7, 135.0, 134.4, 126.9, 125.4, 121.1, 68.1, 62.5, 39.8, 34.1, 34.0, 31.9, 29.69, 29.66, 29.64, 29.63, 29.61, 29.48, 29.46, 29.35, 29.27, 29.1, 29.0, 24.8, 24.6, 22.7, 14.1. Anal. Calcd. for $C_{46}H_{79}NO_7S$: C, 69.92; H, 10.08; N, 1.77; O, 14.17; S, 4.06. Found: C, 69.65; H, 9.94; N, 2.53; O, 14.25; S, 5.02.

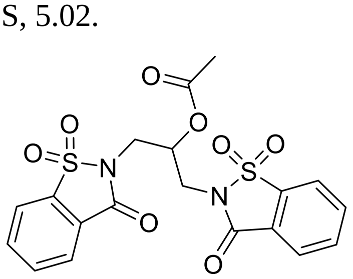

1,3-Bis(1,1-dioxido-3-oxobenzo[*d*]isothiazol-2(3*H*)-yl)propan-2-yl acetate (3emm): 0.025 mmol of Sc(OTf)$_3$ was used, **3em** was used as substrate. White solid, 88% yield, mp 189-190 °C. ^{1}H NMR (500 MHz, DMSO) δ 8.33 (d, *J* = 7.7 Hz, 2H), 8.14 (d, *J* = 7.6 Hz, 2H), 8.08 (t, *J* = 7.5 Hz, 2H), 8.02 (t, *J* = 7.5 Hz, 2H), 5.61 – 5.51 (m, 1H), 4.14 (dd, *J* = 15.4, 3.0 Hz, 2H), 4.07 (dd, *J* = 15.5, 8.6 Hz, 2H), 1.90 (s, 3H). ^{13}C NMR (126 MHz, DMSO) δ 170.6, 159.5, 137.2, 136.5, 135.8, 126.5, 125.7, 122.2, 68.0, 21.2. HR-

MS (ESI-TOF) calcd for $C_{19}H_{17}N_2O_8S_2^+$[M+H]$^+$: 465.0421, found 465.0425.

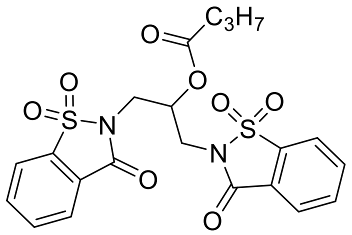

1,3-Bis(1,1-dioxido-3-oxobenzo[*d*]isothiazol-2(3*H*)-yl)propan-2-yl butyrate (3fmm): 0.025 mmol of $Sc(OTf)_3$ was used, **3fm** was used as substrate. White solid, 86% yield, mp 112-113 °C. ^{1}H NMR (500 MHz, $CDCl_3$) δ 8.08 (d, *J* = 7.5 Hz, 1H), 7.96 – 7.82 (m, 3H), 5.68 – 5.61 (m, 1H), 4.14 (dd, *J* = 15.4, 3.6 Hz, 2H), 4.03 (dd, *J* = 15.3, 7.0 Hz, 2H), 2.38 (t, *J* = 7.5 Hz, 2H), 1.65 – 1.56 (m, 2H), 0.87 (t, *J* = 7.4 Hz, 3H). ^{13}C NMR (126 MHz, $CDCl_3$) δ 173.1, 159.4, 137.6, 135.0, 134.4, 126.9, 125.4, 121.1, 67.9, 40.7, 35.8, 17.7, 13.5. HR-MS (ESI-TOF) calcd for $C_{21}H_{24}N_3O_8S_2^+$ [M+NH_4]$^+$: 510.0999, found 510.1000.

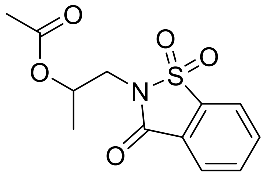

1-(1,1-Dioxido-3-oxobenzo[*d*]isothiazol-2(3*H*)-yl)propan-2-yl acetate (3km): The standard procedure was followed. White solid, 80% yield, mp 90-91 °C. ^{1}H NMR (500 MHz, $CDCl_3$) δ 8.07 (d, *J* = 7.5 Hz, 1H), 7.93 (d, *J* = 7.4 Hz, 1H), 7.88 (t, *J* = 7.0 Hz, 1H), 7.84 (t, *J* = 7.5 Hz, 1H), 5.38 – 5.31 (m, 1H), 3.89 (dd, *J* = 15.1, 3.7 Hz, 1H), 3.84 (dd, *J* = 15.1, 7.6 Hz, 1H), 2.06 (s, 3H), 1.36 (d, *J* = 6.5 Hz, 3H). ^{13}C NMR (126 MHz, $CDCl_3$) δ 170.7, 159.4, 137.7, 134.9, 134.4, 127.0, 125.3, 121.0, 67.5, 43.8, 21.2, 17.7. HR-MS (ESI-TOF) calcd for $C_{12}H_{13}NO_5SNa^+$[M+Na]$^+$: 306.0407, found 306.0403.

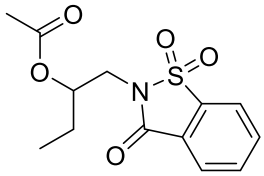

1-(1,1-Dioxido-3-oxobenzo[*d*]isothiazol-2(3*H*)-yl)butan-2-yl acetate (3lm): 2 mmol of substrate **1l** was used. Reaction time: 48 h. White solid, 70% yield, mp 104-105 °C. ^{1}H NMR (500 MHz, $CDCl_3$) δ 8.06 (d, *J* = 7.4 Hz, 1H), 7.92 (d, *J* = 7.5 Hz, 1H), 7.87 (t, *J* = 7.4 Hz, 1H), 7.83 (t, *J* = 7.4 Hz, 1H), 5.26 – 5.20 (m, 1H), 3.92 (dd, *J* = 15.1, 2.8 Hz, 1H), 3.82 (dd, *J* = 15.1, 7.9 Hz, 1H), 2.07 (s, 3H), 1.78 – 1.64 (m, 2H), 1.01 (t, *J* = 7.4 Hz, 3H). ^{13}C NMR (126 MHz, $CDCl_3$) δ 171.0, 159.4, 137.7, 134.9, 134.3, 127.0, 125.3, 121.0, 71.9, 42.7, 25.0, 21.1, 9.5. HR-MS (ESI-TOF) calcd for

$C_{13}H_{16}NO_5S^+$[M+H]$^+$: 298.0744, found 298.0738.

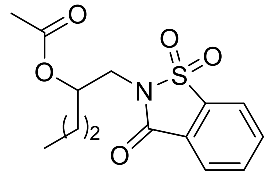

1-(1,1-Dioxido-3-oxobenzo[*d*]isothiazol-2(3*H*)-yl)pentan-2-yl acetate (3mm): 2 mmol of substrate **1m** was used. Reaction time: 48 h. White solid, 67% yield, mp 97-98 °C. ^{1}H NMR (500 MHz, $CDCl_3$) δ 8.06 (d, *J* = 7.4 Hz, 1H), 7.91 (d, *J* = 7.4 Hz, 1H), 7.87 (t, *J* = 7.2 Hz, 1H), 7.83 (t, *J* = 7.4 Hz, 1H), 5.33 – 5.27 (m, 1H), 3.92 (dd, *J* = 15.1, 2.8 Hz, 1H), 3.81 (dd, *J* = 15.1, 7.9 Hz, 1H), 2.06 (s, 3H), 1.70 – 1.62 (m, 2H), 1.50 – 1.37 (m, 2H), 0.96 (t, *J* = 7.3 Hz, 3H). ^{13}C NMR (126 MHz, $CDCl_3$) δ 171.0, 159.5, 137.7, 134.9, 134.3, 127.0, 125.3, 121.0, 70.5, 43.0, 33.9, 21.1, 18.5, 13.8. HR-MS (ESI-TOF) calcd for $C_{14}H_{18}NO_5S^+$[M+H]$^+$: 312.0900, found 312.0891.

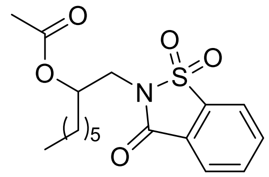

1-(1,1-Dioxido-3-oxobenzo[*d*]isothiazol-2(3*H*)-yl)octan-2-yl acetate (3nm): 2 mmol of substrate **1n** was used. Reaction time 48 h. White solid, 58% yield, mp 91-92 °C. ^{1}H NMR (500 MHz, $CDCl_3$) δ 8.06 (t, *J* = 5.9 Hz, 1H), 7.91 (d, *J* = 7.2 Hz, 1H), 7.89 – 7.85 (m, 1H), 7.85 – 7.81 (m, 1H), 5.33 – 5.25 (m, 1H), 3.92 (dd, *J* = 15.1, 2.9 Hz, 1H), 3.81 (dd, *J* = 15.1, 7.9 Hz, 1H), 2.06 (s, 3H), 1.70 – 1.63 (m, 2H), 1.46 – 1.22 (m, 8H), 0.88 (t, *J* = 6.8 Hz, 3H). ^{13}C NMR (126 MHz, $CDCl_3$) δ 171.0, 159.4, 137.7, 134.8, 134.3, 127.0, 125.2, 121.0, 70.7, 43.0, 31.9, 31.6, 28.9, 25.1, 22.5, 21.1, 14.0. HR-MS (ESI-TOF) calcd for $C_{17}H_{24}NO_5S^+$[M+H]$^+$: 354.1370, found 354.1360.

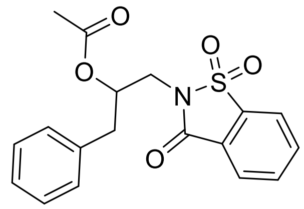

1-(1,1-Dioxido-3-oxobenzo[*d*]isothiazol-2(3*H*)-yl)-3-phenylpropan-2-yl acetate (3om): Reaction time 48 h. White solid, 78% yield, mp 112-113 °C. ^{1}H NMR (500 MHz, $CDCl_3$) δ 8.05 (d, *J* = 7.3 Hz, 1H), 7.91 (d, *J* = 7.4 Hz, 1H), 7.86 (t, *J* = 7.0 Hz, 1H), 7.82 (t, *J* = 7.5 Hz, 1H), 7.34 – 7.29 (m, 2H), 7.29 – 7.22 (m, 3H), 5.57 – 5.49 (m, 1H), 3.96 (dd, *J* = 15.1, 2.9 Hz, 1H), 3.83 (dd, *J* = 15.1, 8.1 Hz, 1H), 2.99 (d, *J* = 6.3 Hz, 2H), 1.99 (s, 3H). ^{13}C NMR (126 MHz, $CDCl_3$) δ 170.6, 159.4, 137.6, 136.1, 134.9,

134.4, 129.3, 128.5, 126.94, 126.89, 125.3, 121.0, 71.0, 42.6, 38.3, 21.0. HR-MS (ESI-TOF) calcd for $C_{18}H_{18}NO_5S^+$[M+H]$^+$: 360.0900, found 360.0890.

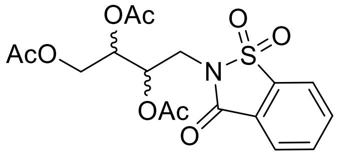

4-(1,1-dioxido-3-oxobenzo[*d*]isothiazol-2(3*H*)-yl)butane-1,2,3-triyl triacetate (3pm): The standard procedure was followed, (2*R*,3*S*)-butane-1,2,3,4-tetrayl tetraacetate (**1p**) was used as substrate. Colorless oil, 70% yield. Mixture of diastereomers. ^{1}H NMR (500 MHz, $CDCl_3$) δ 8.06 (d, *J* = 7.3 Hz, 1H), 7.94 – 7.81 (m, 3H), 5.66 – 5.19 (m, 2H), 4.45 – 3.80 (m, 4H), 2.19 – 2.06 (m, 9H). One diastereomer was obtained by recrystallization from isopropanol. White solid, 22% yield, mp 46-47 °C. ^{1}H NMR (500 MHz, CDCl3) δ 8.06 (d, J = 7.4 Hz, 1H), 7.94 – 7.81 (m, 3H), 5.66 – 5.54 (m, 1H), 5.38 – 5.34 (m, 1H), 4.33 (dd, J = 11.8, 5.1 Hz, 1H), 4.15 (dd, J = 11.8, 6.2 Hz, 1H), 4.01 (dd, J = 15.1, 3.5 Hz, 1H), 3.86 (dd, J = 15.1, 8.7 Hz, 1H), 2.18 (s, 3H), 2.10 (s, 3H), 2.08 (s, 3H). ^{13}C NMR (126 MHz, $CDCl_3$) δ 170.4, 170.3, 170.0, 159.2, 137.5, 135.0, 134.4, 126.8, 125.3, 121.1, 69.4, 68.0, 61.5, 39.9, 20.74, 20.72, 20.6. HR-MS (ESI-TOF) calcd for $C_{17}H_{23}N_2O_9S^+$ [M+NH_4]$^+$: 431.1119, found 431.1110.

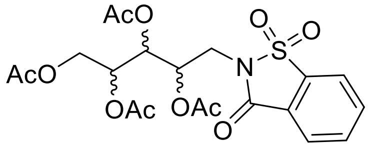

5-(1,1-Dioxido-3-oxobenzo[*d*]isothiazol-2(3*H*)-yl)pentane-1,2,3,4-tetrayl tetraacetate (3qm): The standard procedure was followed, (2*R*,3*r*,4*S*)-pentane-1,2,3,4,5-pentayl pentaacetate (**1q**) was used as substrate. Colorless oil, 48% yield. Mixture of diastereomers. ^{1}H NMR (500 MHz, $CDCl_3$) δ 8.10 – 8.02 (m, 1H), 7.94 – 7.79 (m, 3H), 5.73 – 5.13 (m, 3H), 4.42 – 3.66 (m, 4H), 2.25 – 2.01 (m, 12H). One diastereomer was obtained by recrystallization from isopropanol. White solid, 9% yield, mp 182-184 °C. ^{1}H NMR (500 MHz, $CDCl_3$) δ 8.07 (d, *J* = 7.4 Hz, 1H), 7.93 – 7.80 (m, 3H), 5.74 – 5.66 (m, 1H), 5.49 (dd, *J* = 9.2, 2.1 Hz, 1H), 5.22 – 5.13 (m, 1H), 4.25 (dd, *J* = 12.6, 2.4 Hz, 1H), 4.19 (dd, *J* = 12.6, 4.3 Hz, 1H), 3.95 (dd, *J* = 15.1, 2.6 Hz, 1H), 3.71 (dd, *J* = 15.1, 9.7 Hz, 1H), 2.21 (s, 3H), 2.09 (s, 3H), 2.05 (s, 3H), 2.04 (s, 3H). ^{13}C NMR (126 MHz, $CDCl_3$) δ 170.61, 170.58, 169.9, 169.8, 159.1, 137.4, 135.0, 134.4, 126.7, 125.4, 121.0, 68.8, 67.9, 67.0, 61.5, 40.7, 20.74, 20.70, 20.67. HR-MS (ESI-TOF) calcd for $C_{20}H_{27}N_2O_{11}S^+$ [M+NH_4]$^+$: 503.1330, found 503.1329.

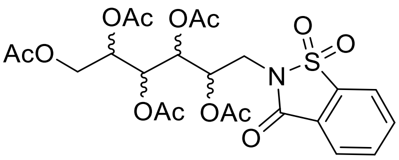

6-(1,1-Dioxido-3-oxobenzo[*d*]isothiazol-2(3*H*)-yl)hexane-1,2,3,4,5-pentayl pentaacetate (3rm): The standard procedure was followed, (2*R*,3*S*,4*S*,5*S*)-hexane-1,2,3,4,5,6-hexayl hexaacetate (**1r**) was used as substrate. Colorless oil, 43% yield. Mixture of diastereomers. ^{1}H NMR (500 MHz, $CDCl_3$) δ 8.08 – 8.03 (m, 1H), 7.95 – 7.79 (m, 3H), 5.71 – 5.05 (m, 4H), 4.43 – 3.57 (m, 4H), 2.31 – 1.96 (m, 15H). Two diastereoisomers were obtained by recrystallization twice from isopropanol in ratio of 1:0.82 judging from the NMR. White solid, 4% yield, mp 161-163 °C. ^{1}H NMR (500 MHz, $CDCl_3$) δ 8.07 – 8.03 (m), 7.93 – 7.80 (m), 5.64 – 5.60 (m), 5.51 (dd, *J* = 9.1, 2.4 Hz), 5.47 – 5.42 (m), 5.38 – 5.31 (m), 5.14 – 5.09 (m), 4.29 (dd, *J* = 11.7, 4.6 Hz), 4.22 (dd, *J* = 12.5, 2.8 Hz), 4.09 (dd, *J* = 12.5, 5.1 Hz), 3.93 – 3.82 (m), 3.63 (dd, *J* = 15.1, 10.0 Hz), 2.19 (s), 2.18 (s), 2.12 (s), 2.12 (s), 2.08 (s), 2.07 (s), 2.06 (s), 2.05 (s), 2.03 (s). ^{13}C NMR (126 MHz, $CDCl_3$) δ 171.0, 170.6, 170.5, 170.44, 170.38, 170.23, 170.18, 170.0, 169.9, 169.8, 159.3, 159.2, 137.7, 137.5, 135.03, 134.99, 134.45 134.43, 126.8, 125.40, 125.38, 121.1, 68.9, 68.3, 67.9, 67.6, 67.52, 67.50, 67.48, 66.5, 62.3, 61.8, 41.1, 40.8, 21.0, 20.88, 20.87, 20.77, 20.75, 20.71, 20.68, 20.6. HR-MS (ESI-TOF) calcd for $C_{23}H_{31}N_2O_{13}S^+$ $[M+NH_4]^+$: 575.1541, found 575.1537.

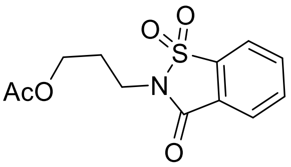

3-(1,1-Dioxido-3-oxobenzo[*d*]isothiazol-2(3*H*)-yl)propyl acetate (3sm): The standard procedure was followed. White solid, 73% yield, mp 123-124 °C. ^{1}H NMR (400 MHz, $CDCl_3$) δ 8.07 (d, *J* = 7.3 Hz, 1H), 7.97 – 7.81 (m, 3H), 4.19 (t, *J* = 5.9 Hz, 2H), 3.90 (t, *J* = 7.0 Hz, 2H), 2.27 – 2.15 (m, 2H), 2.08 (s, 3H). ^{13}C NMR (101 MHz, $CDCl_3$) δ 171.0, 158.9, 137.5, 134.8, 134.4, 127.2, 125.1, 120.9, 61.3, 36.1, 27.3, 20.8. HR-MS (ESI-TOF) calcd for $C_{12}H_{14}NO_5S^+$$[M+H]^+$: 284.0587, found 284.0579.

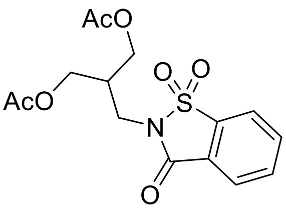

2-((1,1-Dioxido-3-oxobenzo[*d*]isothiazol-2(3*H*)-yl)methyl)propane-1,3-diyl

diacetate (3tm): 0.025 mmol of $Sc(OTf)_3$ was used. White solid, 46% yield, mp 127-128 °C. ^{1}H NMR (500 MHz, $CDCl_3$) δ 8.07 (d, *J* = 7.4 Hz, 1H), 7.94 (d, *J* = 7.4 Hz, 1H), 7.90 (t, *J* = 7.1 Hz, 1H), 7.86 (t, *J* = 7.5 Hz, 1H), 4.24 – 4.15 (m, 4H), 3.89 (d, *J* = 7.4 Hz, 2H), 2.79 – 2.70 (m, 1H), 2.08 (s, 6H). ^{13}C NMR (126 MHz, $CDCl_3$) δ 170.7, 159.2, 137.4, 134.9, 134.4, 126.9, 125.2, 121.0, 62.1, 37.7, 36.6, 20.7. HR-MS (ESI-TOF) calcd for $C_{15}H_{21}N_2O_7S^+$[M+NH$_4$]$^+$: 373.1064, found 373.1058.

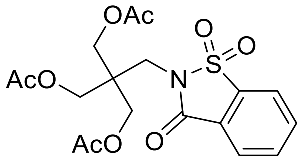

2-(Acetoxymethyl)-2-((1,1-dioxido-3-oxobenzo[*d*]isothiazol-2(3*H*)-yl)methyl)propane-1,3-diyl diacetate (3um): 0.025 mmol of $Hf(OTf)_4$ was used, and a drying tube filled with K_2CO_3 and Na_2SO_4 was equipped to assimilate AcOH during reaction. White solid, 30% yield, mp 138-140 °C. ^{1}H NMR (500 MHz, $CDCl_3$) δ 8.07 (d, *J* = 7.4 Hz, 1H), 7.97 – 7.83 (m, 3H), 4.18 (s, 6H), 3.96 (s, 2H), 2.11 (s, 9H). ^{13}C NMR (126 MHz, $CDCl_3$) δ 170.5, 160.7, 137.7, 135.2, 134.5, 126.7, 125.4, 121.2, 63.0, 42.2, 39.9, 20.7. HR-MS (ESI-TOF) calcd for $C_{18}H_{21}NO_9SNa^+$[M+Na]$^+$: 450.0829, found 450.0831.

Procedures for the preparation of a dioxolanium salt and the reaction of saccharin/saccharin sodium with the salt

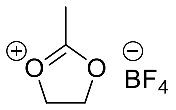

Procedures from reference[17] were modified and the whole reaction was performed in grove box. To a solution of $AgBF_4$ (2 mmol) in CH_3CN (1 ml), 2-bromoethyl acetate (2 mmol in 1 ml CH_3CN) was added. The mixture was left in the dark at room temperature for 12 hours. The precipitated AgBr was filtered and washed with CH_3CN. The combined CH_3CN solution was added dropwise into ether (20 ml) with stirring. The solid precipitated was filtered and dried, affording 2-Methyl-l,3-dioxolan-2-ylium fluoroborate as a colorless crystalline salt in 91% of yield. ^{1}H NMR (500 MHz, CD_3CN) δ 5.27 (s, 4H), 2.73 (s, 3H). Characterization data are consistent with literature value.[18]

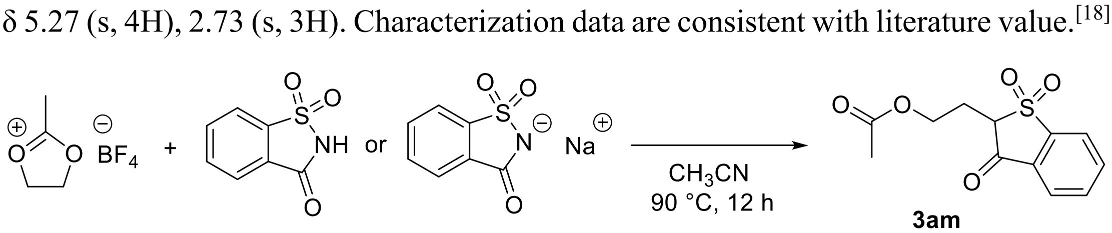

2-Methyl-l,3-dioxolan-2-ylium fluoroborate (0.5 mmol), saccharin or saccharin sodium

(0.5 mmol) and CH_3CN (2 ml) were added to a 5 mL sample vial equipped with a magnetic stir bar. The vial was sealed and stirred at 90 °C for 12 hours. After completion by TLC, the mixture was cooled down to room temperature and purified by column chromatography (PE : EA = 4 : 1) to afford the target product **3am** in yield of 49% and 16% for saccharin and saccharin sodium, respectively.

Standard procedure for the substitution of amide ester with saccharin

Amide ester (0.6 mmol), saccharin (0.5 mmol) and $Hf(OTf)_4$ (0.005 mmol) were added to a 5 ml sample vial equipped with a magnetic stir bar. The vial was sealed and stirred at 150 °C for 24 h. After completion by TLC, the mixture was cooled down to room temperature and purified by column chromatography (PE : EA = 1 : 1) to afford the target product.

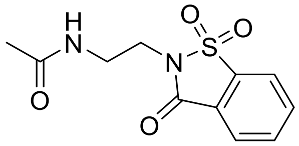

***N*-(2-(1,1-dioxido-3-oxobenzo[*d*]isothiazol-2(3*H*)-yl)ethyl)acetamide (5am):** The standard procedure was followed. White solid, 92% yield, mp 139-140 °C. ^{1}H NMR (500 MHz, $CDCl_3$) δ 8.08 (d, J = 7.4 Hz, 1H), 7.98 – 7.84 (m, 3H), 6.17 (s, 1H), 3.96 – 3.91 (m, 2H), 3.65 (dd, J = 10.9, 5.7 Hz, 2H), 1.98 (s, 3H). ^{13}C NMR (126 MHz, $CDCl_3$) δ 170.7, 159.3, 137.4, 135.1, 134.6, 127.1, 125.3, 121.1, 39.5, 38.1, 23.2. HR-MS (ESI-TOF) calcd for $C_{11}H_{13}N_2O_4S^+$[M+H]$^+$: 269.0591, found 269.0585.

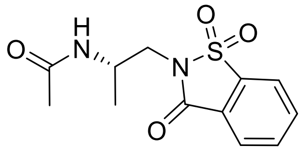

(*S*)-*N*-(1-(1,1-dioxido-3-oxobenzo[*d*]isothiazol-2(3*H*)-yl)propan-2-yl)acetamide (5bm): The standard procedure was followed. White solid, 90% yield, mp 164-165 °C. ^{1}H NMR (500 MHz, $CDCl_3$) δ 8.08 (d, J = 7.4 Hz, 1H), 7.98 – 7.84 (m, 3H), 6.04 (d, J = 7.7 Hz, 1H), 4.56 – 4.46 (m, 1H), 3.92 – 3.81 (m, 2H), 1.97 (s, 3H), 1.24 (d, J = 6.8 Hz, 3H). ^{13}C NMR (126 MHz, $CDCl_3$) δ 170.0, 159.7, 137.3, 135.0, 134.5, 127.0, 125.3, 121.1, 44.5, 44.1, 23.4, 17.8. HR-MS (ESI-TOF) calcd for $C_{12}H_{15}N_2O_4S^+$[M+H]$^+$: 283.0747, found 283.0743. $[\alpha]_D^{20}$ = -20.2 (c = 1.0, $CHCl_3$).

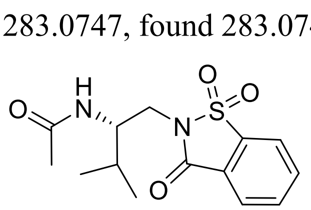

(*S*)-*N*-(1-(1,1-dioxido-3-oxobenzo[*d*]isothiazol-2(3*H*)-yl)-3-methylbutan-2-yl)

acetamide (5cm): The standard procedure was followed. White solid, 94% yield, mp 147-148 °C. ^{1}H NMR (500 MHz, $CDCl_3$) δ 8.05 (d, *J* = 7.4 Hz, 1H), 7.92 (d, *J* = 7.3 Hz, 1H), 7.88 (t, *J* = 6.9 Hz, 1H), 7.84 (t, *J* = 6.8 Hz, 1H), 5.90 (d, *J* = 9.5 Hz, 1H), 4.33 – 4.26 (m, 1H), 3.87 (dd, *J* = 14.9, 3.8 Hz, 1H), 3.81 (dd, *J* = 14.9, 8.6 Hz, 1H), 1.96 (s, 3H), 1.93 – 1.85 (m, 1H), 1.06 (d, *J* = 6.8 Hz, 3H), 1.00 (d, *J* = 6.8 Hz, 3H). ^{13}C NMR (126 MHz, $CDCl_3$) δ 170.5, 159.5, 137.2, 134.8, 134.4, 127.0, 125.2, 120.9, 52.8, 41.8, 30.0, 23.3, 19.2, 18.2. HR-MS (ESI-TOF) calcd for $C_{14}H_{19}N_2O_4S^+$[M+H]$^+$: 311.1060, found 311.1059. $[\alpha]_D^{20}$ = -7.3 (c = 1.0, $CHCl_3$).

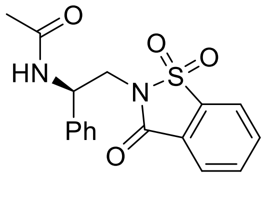

(*R*)-*N*-(2-(1,1-dioxido-3-oxobenzo[*d*]isothiazol-2(3*H*)-yl)-1-phenylethyl)acetamide (5dm): The standard procedure was followed. White solid, 90% yield, mp 164-166 °C. ^{1}H NMR (500 MHz, $CDCl_3$) δ 8.05 (d, *J* = 7.5 Hz, 1H), 7.93 (d, *J* = 7.5 Hz, 1H), 7.88 (t, *J* = 7.4 Hz, 1H), 7.84 (t, *J* = 7.4 Hz, 1H), 7.43 – 7.34 (m, 4H), 7.31 (t, *J* = 6.9 Hz, 1H), 6.52 (d, *J* = 7.2 Hz, 1H), 5.55 (dd, *J* = 14.3, 7.3 Hz, 1H), 4.03 (d, *J* = 6.7 Hz, 2H), 1.98 (s, 3H). ^{13}C NMR (126 MHz, $CDCl_3$) δ 170.0, 159.4, 138.3, 137.2, 135.0, 134.5, 128.9, 128.1, 126.9, 126.5, 125.4, 52.1, 44.2, 23.3. HR-MS (ESI-TOF) calcd for $C_{17}H_{17}N_2O_4S^+$[M+H]$^+$: 345.0904, found 345.0902. Recrystallization from isopropanol afforded white needle crystals. Single-crystal XRD data and parameters are shown in pp. 6-14 of This document. $[\alpha]_D^{20}$ = -49.5 (c = 1.0, $CHCl_3$).

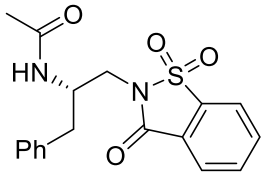

(*S*)-*N*-(1-(1,1-dioxido-3-oxobenzo[*d*]isothiazol-2(3*H*)-yl)-3-phenylpropan-2-yl) acetamide (5em): The standard procedure was followed. White solid, 95% yield, mp 127-128 °C. ^{1}H NMR (500 MHz, $CDCl_3$) δ 8.06 (d, *J* = 7.1 Hz, 1H), 7.92 (d, *J* = 7.2 Hz, 1H), 7.91 – 7.82 (m, 2H), 7.32 (t, *J* = 7.3 Hz, 2H), 7.27 – 7.22 (m, 3H), 5.89 (d, *J* = 8.6 Hz, 1H), 4.75 – 4.66 (m, 1H), 3.91 (dd, *J* = 15.0, 3.9 Hz, 1H), 3.86 (dd, *J* = 15.0, 7.6 Hz, 1H), 2.96 (dd, *J* = 14.2, 6.3 Hz, 1H), 2.90 (dd, *J* = 14.2, 7.5 Hz, 1H), 1.90 (s, 3H). ^{13}C NMR (126 MHz, $CDCl_3$) δ 170.3, 159.6, 137.3, 136.6, 135.0, 134.5, 129.1, 128.6, 127.0, 126.8, 125.3, 121.0, 48.9, 43.0, 38.0, 23.3. HR-MS (ESI-TOF) calcd for $C_{18}H_{19}N_2O_4S^+$[M+H]$^+$: 359.1060, found 359.1056. $[\alpha]_D^{20}$ = 9.9 (c = 1.0, $CHCl_3$).

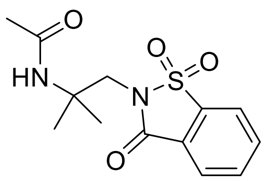

***N*-(1-(1,1-dioxido-3-oxobenzo[*d*]isothiazol-2(3*H*)-yl)-2-methylpropan-2-yl)acetamide (5fm):** The standard procedure was followed. White solid, 93% yield, mp 181-182 °C. ^{1}H NMR (500 MHz, $CDCl_3$) δ 8.07 (d, *J* = 7.5 Hz, 1H), 7.95 (d, *J* = 7.5 Hz, 1H), 7.91 (t, *J* = 7.3 Hz, 1H), 7.86 (t, *J* = 7.4 Hz, 1H), 5.95 (s, 1H), 4.08 (s, 2H), 1.98 (s, 3H), 1.47 (s, 6H). ^{13}C NMR (126 MHz, $CDCl_3$) δ 170.5, 160.8, 137.7, 135.1, 134.4, 126.8, 125.3, 121.1, 53.8, 47.8, 25.0, 24.4. HR-MS (ESI-TOF) calcd for $C_{13}H_{17}N_2O_4S^+[M+H]^+$: 297.0904, found 297.0900.

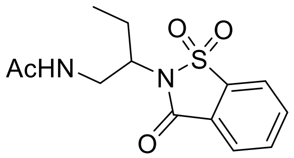

***N*-(2-(1,1-dioxido-3-oxobenzo[*d*]isothiazol-2(3*H*)-yl)butyl)acetamide (5gm):** The standard procedure was followed. White solid, 90% yield, mp 136-137 °C. ^{1}H NMR (500 MHz, $CDCl_3$) δ 8.06 (d, *J* = 7.5 Hz, 1H), 7.94 – 7.84 (m, 3H), 6.07 (s, 1H), 4.33 – 4.22 (m, 1H), 3.82 – 3.71 (m, 2H), 2.21 – 2.10 (m, 1H), 1.93 (s, 3H), 1.92 – 1.84 (m, 1H), 1.01 (t, *J* = 7.4 Hz, 3H). ^{13}C NMR (126 MHz, $CDCl_3$) δ 170.5, 159.0, 137.2, 134.9, 134.5, 127.0, 125.2, 120.8, 57.0, 40.6, 23.2, 10.9. HR-MS (ESI-TOF) calcd for $C_{13}H_{17}N_2O_4S^+[M+H]^+$: 297.0904, found 297.0903

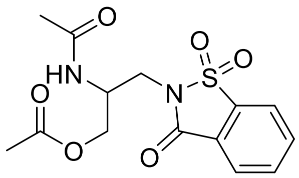

2-Acetamido-3-(1,1-dioxido-3-oxobenzo[*d*]isothiazol-2(3*H*)-yl)propyl acetate (5hm): 1 mmol of substrate **5h** was used. White solid, 80% yield, mp 111-112 °C. ^{1}H NMR (500 MHz, $CDCl_3$) δ 8.09 (t, *J* = 9.3 Hz, 1H), 7.97 – 7.84 (m, 3H), 6.22 (d, *J* = 8.6 Hz, 1H), 4.76 – 4.68 (m, 1H), 4.24 (dd, *J* = 11.5, 4.7 Hz, 1H), 4.16 (dd, *J* = 11.5, 5.4 Hz, 1H), 4.00 (dd, *J* = 15.1, 7.3 Hz, 1H), 3.91 (dd, *J* = 15.1, 4.0 Hz, 1H), 2.10 (s, 3H), 1.99 (s, 3H). ^{13}C NMR (126 MHz, $CDCl_3$) δ 170.7, 170.3, 159.6, 137.3, 135.1, 134.6, 126.8, 125.4, 121.1, 63.4, 46.9, 40.6, 23.2, 20.7. HR-MS (ESI-TOF) calcd for $C_{14}H_{17}N_2O_6S^+[M+H]^+$: 341.0802, found 341.0802.

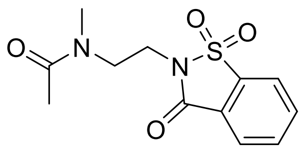

***N*-(2-(1,1-dioxido-3-oxobenzo[*d*]isothiazol-2(3*H*)-yl)ethyl)-*N*-methylacetamide (5im):** The standard procedure was followed. White solid, 94% yield, mp 148-150 °C. Mixture of stereoisomers about the amide bond in ratio of 2:1. ^{1}H NMR (500 MHz, $CDCl_3$) δ 8.10 – 8.05 (m, *J* = 6.7 Hz, 1H), 7.98 – 7.82 (m, 3H), 3.99 – 3.90 (m, 2H), 3.78 (t, *J* = 5.5 Hz, 1.4H), 3.72 (t, *J* = 7.0 Hz, 0.6H), 3.08 – 3.01 (m, 3H), 2.16 (s, 0.9H), 2.06 (s, 2.1H). Major: ^{13}C NMR (126 MHz, $CDCl_3$) δ 171.5, 159.1, 137.6, 134.8, 134.3, 127.0, 125.2, 120.9, 46.2, 37.28, 37.25, 21.8. Minor: ^{13}C NMR (126 MHz, $CDCl_3$) δ 170.7, 158.8, 137.4, 135.1, 134.6, 126.8, 125.3, 121.1, 48.5, 36.5, 33.4, 21.1. HR-MS (ESI-TOF) calcd for $C_{12}H_{14}N_2O_4SNa^+$[M+Na]$^+$: 305.0566, found 305.0558.

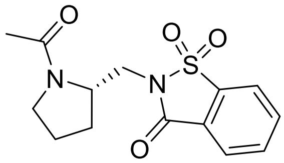

(*S*)-2-((1-acetylpyrrolidin-2-yl)methyl)benzo[*d*]isothiazol-3(2*H*)-one 1,1-dioxide (5jm): The standard procedure was followed. Colorless oil, 88% yield. Mixture of stereoisomers about the amide bond in ratio of 3:1 judging by NMR. ^{1}H NMR (500 MHz, $CDCl_3$) δ 8.03 – 7.97 (m, 1H), 7.92 – 7.73 (m, 3H), 4.58 – 4.50 (m, 0.75H), 4.34 – 4.27 (m, 0.25H), 3.97 (dd, *J* = 14.7, 5.8 Hz, 0.75H), 3.89 (dd, *J* = 14.7, 5.2 Hz, 0.75H), 3.74 – 3.58 (m, 0.5H), 3.55 – 3.45 (m, 1.25H), 3.35 (dd, *J* = 17.4, 7.7 Hz, 0.75H), 2.21 (s, 0.75H), 2.07 – 1.83 (m, 6.25H). Major: ^{13}C NMR (126 MHz, $CDCl_3$) δ 170.4, 159.9, 137.7, 134.8, 134.3, 127.1, 125.2, 120.9, 55.1, 47.7, 41.1, 27.8, 23.9, 22.8. Minor: ^{13}C NMR (126 MHz, $CDCl_3$) δ 169.7, 159.4, 137.3, 135.2, 134.6, 126.8, 125.3, 121.2, 56.4, 45.6, 41.1, 28.9, 22.1, 21.4. HR-MS (ESI-TOF) calcd for $C_{14}H_{17}N_2O_4S^+$[M+H]$^+$: 309.0904, found 309.0901. $[\alpha]_D^{20}$ = -16.1 (c = 1.0, $CHCl_3$).

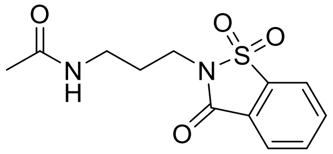

***N*-(3-(1,1-dioxido-3-oxobenzo[*d*]isothiazol-2(3*H*)-yl)propyl)acetamide (5km):** The standard procedure was followed. White solid, 88% yield, mp 73-75 °C. ^{1}H NMR (500 MHz, $CDCl_3$) δ 8.07 (d, *J* = 7.4 Hz, 1H), 7.95 (d, *J* = 7.4 Hz, 1H), 7.91 (t, *J* = 7.4 Hz, 1H), 7.87 (t, *J* = 7.3 Hz, 1H), 6.24 (s, 1H), 3.86 (t, *J* = 6.5 Hz, 2H), 3.33 (dd, *J* = 12.4, 6.2 Hz, 2H), 2.11 – 1.98 (m, 5H). ^{13}C NMR (126 MHz, $CDCl_3$) δ 170.4, 159.4, 137.4,

134.9, 134.5, 127.0, 125.2, 121.0, 36.4, 36.1, 27.9, 23.3. HR-MS (ESI-TOF) calcd for $C_{12}H_{15}N_2O_4S^+[M+H]^+$: 283.0747, found 283.0742.

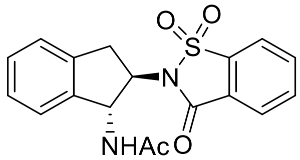

***N*-((1*R*,2*R*)-2-(1,1-dioxido-3-oxobenzo[*d*]isothiazol-2(3*H*)-yl)-2,3-dihydro-1*H*-inden-1-yl)acetamide (5lm):** The standard procedure was followed. White solid, 90% yield from *cis* substrate **4l**, 94% yield from *trans* substrate **4l'**, mp 212-214 °C. ^{1}H NMR (500 MHz, $CDCl_3$) δ 8.07 (d, *J* = 7.3 Hz, 1H), 7.91 – 7.80 (m, 3H), 7.30 – 7.21 (m, 4H), 6.30 (t, *J* = 9.1 Hz, 1H), 6.01 (d, *J* = 8.8 Hz, 1H), 4.48 (dd, *J* = 18.9, 9.3 Hz, 1H), 3.80 (dd, *J* = 14.9, 10.9 Hz, 1H), 3.22 (dd, *J* = 15.2, 8.4 Hz, 1H), 1.98 (s, 3H). ^{13}C NMR (126 MHz, $CDCl_3$) δ 171.1, 159.2, 139.8, 138.7, 137.1, 134.7, 134.4, 128.3, 127.4, 127.3, 125.3, 124.8, 123.5, 120.9, 59.8, 55.3, 32.3, 23.1. HR-MS (ESI-TOF) calcd for $C_{18}H_{17}N_2O_4S^+[M+H]^+$: 357.0904, found 357.0900. $[\alpha]_D^{20}$ = -34.5 (c = 1.0, $CHCl_3$)

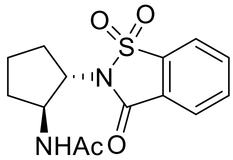

***N*-((1*S*,2*S*)-2-(1,1-dioxido-3-oxobenzo[*d*]isothiazol-2(3*H*)-yl)cyclopentyl) acetamide (5mm):** The standard procedure was followed. White solid, 90% yield, mp 159-161 °C. ^{1}H NMR (500 MHz, $CDCl_3$) δ 8.05 (dd, *J* = 6.6, 1.4 Hz, 1H), 7.90 – 7.80 (m, 3H), 5.85 (d, *J* = 7.2 Hz, 1H), 4.94 – 4.81 (m, 1H), 4.18 (dd, *J* = 18.8, 9.6 Hz, 1H), 2.52 – 2.42 (m, 1H), 2.35 – 2.26 (m, 1H), 2.18 – 2.10 (m, 1H), 2.04 – 1.94 (m, 1H), 1.89 (s, 3H), 1.88 – 1.79 (m, 1H), 1.57 – 1.48 (m, 1H). ^{13}C NMR (126 MHz, $CDCl_3$) δ 170.5, 158.9, 137.1, 134.6, 134.3, 127.4, 125.1, 120.8, 58.3, 52.4, 29.9, 25.8, 23.2, 20.7. HR-MS (ESI-TOF) calcd for $C_{14}H_{17}N_2O_4S^+[M+H]^+$: 309.0904, found 309.0900. $[\alpha]_D^{20}$ = 45.4 (c = 1.0, $CHCl_3$)

Procedure for the substitutive depolymerization of PET

Polyethylene terephthalate (PET) pieces (96 mg, 0.5 mmol), saccharin (92 mg, 0.5 mmol) and $Hf(OTf)_4$ (17.8 mg, 0.025 mmol) were added to a 5 mL sample vial equipped with a magnetic stir bar, followed by the addition of toluene (1 mL). The bottle was then sealed and stirred at 150 °C for 24 hours. After completion, the mixture was cooled down to room temperature and the solvent was removed under vacuum. The residue was treated with 0.5 M aqueous NaOH solution (15 mL). The remaining solid was filtered and washed with water (10 mL), then dried in vacuum to afford the target

degradation product **8** as a white solid in 86% yield.

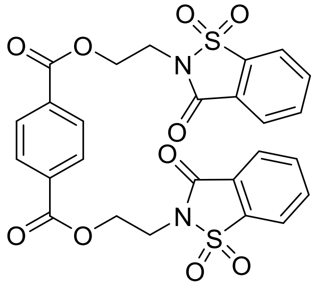

Bis(2-(1,1-dioxido-3-oxobenzo[*d*]isothiazol-2(3*H*)-yl)ethyl) terephthalate (8): White solid, 86% yield, mp 230-232 °C. ^{1}H NMR (500 MHz, DMSO) δ 8.29 (d, *J* = 7.7 Hz, 2H), 8.13 (d, *J* = 7.5 Hz, 2H), 8.09 – 8.03 (m, 6H), 8.00 (t, *J* = 7.5 Hz, 2H), 4.61 (t, *J* = 4.9 Hz, 4H), 4.16 (t, *J* = 4.8 Hz, 4H). ^{13}C NMR (126 MHz, DMSO) δ 165.3, 159.2, 137.2, 136.3, 135.7, 133.9, 129.9, 126.7, 125.6, 122.1, 62.6, 38.4. HR-MS (ESI-TOF) calcd for $C_{26}H_{21}N_2O_{10}S_2^+$[M+H]$^+$: 585.0632, found 585.0617.

One-pot depolymerization of PET to hydroxyl sulfonamide 9

PET pieces (192 mg, 1 mmol), saccharin (183 mg, 1 mmol) and $Hf(OTf)_4$ (35 mg, 0.05 mmol) were added to a 15 mL reaction tube equipped with a magnetic stir bar, followed by the addition of toluene (2 mL). The tube was then sealed and stirred at 150 °C for 24 hours. After completion, 6 M aqueous NaOH solution (4 mL) was added to the mixture after cooling down to room temperature. The resulting mixture was further stirred at 60 °C for 3 hours for hydrolysis. After completion, the mixture was transferred to a separatory funnel followed by the addition of water (20 mL) and ethyl acetate (20 mL). The aqueous phase was acidified to pH ~ 4 by 1 M aqueous HCl solution to precipitate the terephthalic acid out, which was then filtered, washed with water (20 mL), and dried under vacuum as a white solid in 97% yield. The resulting filtrate was extracted with ethyl acetate (20 mL*3). The organic phase was washed with saturated NaCl solution (50 mL), dried with Na_2SO_4 and evaporated under vacuum to give hydroxyl sulfonamide **9** as a white solid in 90% yield.

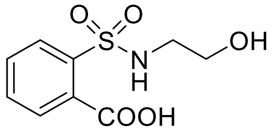

2-(*N*-(2-hydroxyethyl)sulfamoyl)benzoic acid (9): White solid, 90% yield, mp 134-135 °C. ^{1}H NMR (500 MHz, DMSO) δ 13.72 (s, 1H), 8.01 – 7.83 (m, 1H), 7.81 – 7.60 (m, 3H), 7.02 (s, 1H), 4.77 (s, 1H), 3.40 (t, *J* = 5.9 Hz, 2H), 2.89 (d, *J* = 3.7 Hz, 2H). ^{13}C NMR (126 MHz, DMSO) δ 169.4, 138.0, 133.4, 133.1, 131.3, 129.9, 129.0, 60.1, 45.7. HR-MS (ESI-TOF) calcd for $C_9H_{10}NO_5S^-$[M-H]$^-$: 244.0285, found 244.0281.

Gram scale depolymerization of PET

PET pieces (1.92 g, 10 mmol), saccharin (1.83 g, 10 mmol) and $Hf(OTf)_4$ (350 mg, 0.5 mmol) were added to a 50 mL reaction tube equipped with a magnetic stir bar, followed by the addition of toluene (10 mL). The tube was then sealed and stirred at 150 °C for 48 hours. After completion, NaOH (2.40 g, 60 mmol) and water (20 mL) were added to the mixture after cooling down to room temperature. The resulting mixture was further stirred at 60 °C for 12 hours for hydrolysis. After completion, the mixture was transferred to a separatory funnel followed by the addition of water (50 mL) and ethyl acetate (50 mL). The aqueous phase was acidified to pH ~ 4 by 12 M HCl solution to precipitate the terephthalic acid out, which was then filtered, washed with water (20 mL), and dried under vacuum. 1.57 g of product was obtained as a white solid in 95% yield. The resulting filtrate was extracted with ethyl acetate (100 mL*3). The organic phase was washed with saturated NaCl solution (100 mL), dried with Na_2SO_4 and evaporated under vacuum. 2.05 g of hydroxyl sulfonamide **9** was obtained as a white solid in 84% yield.

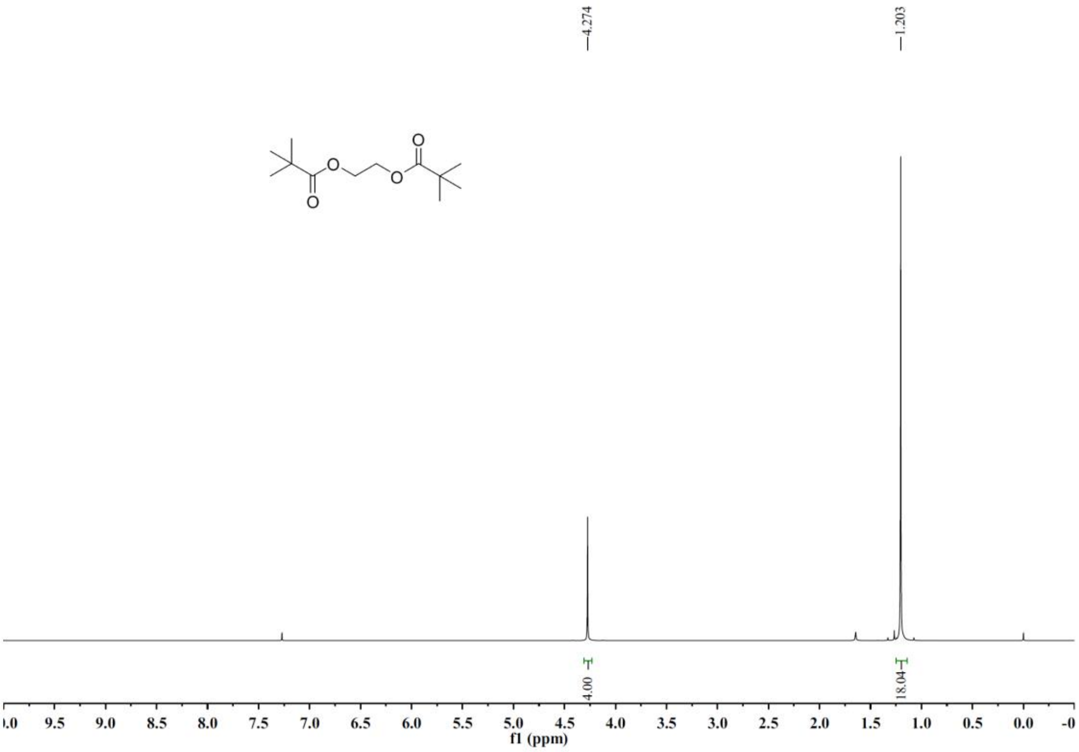

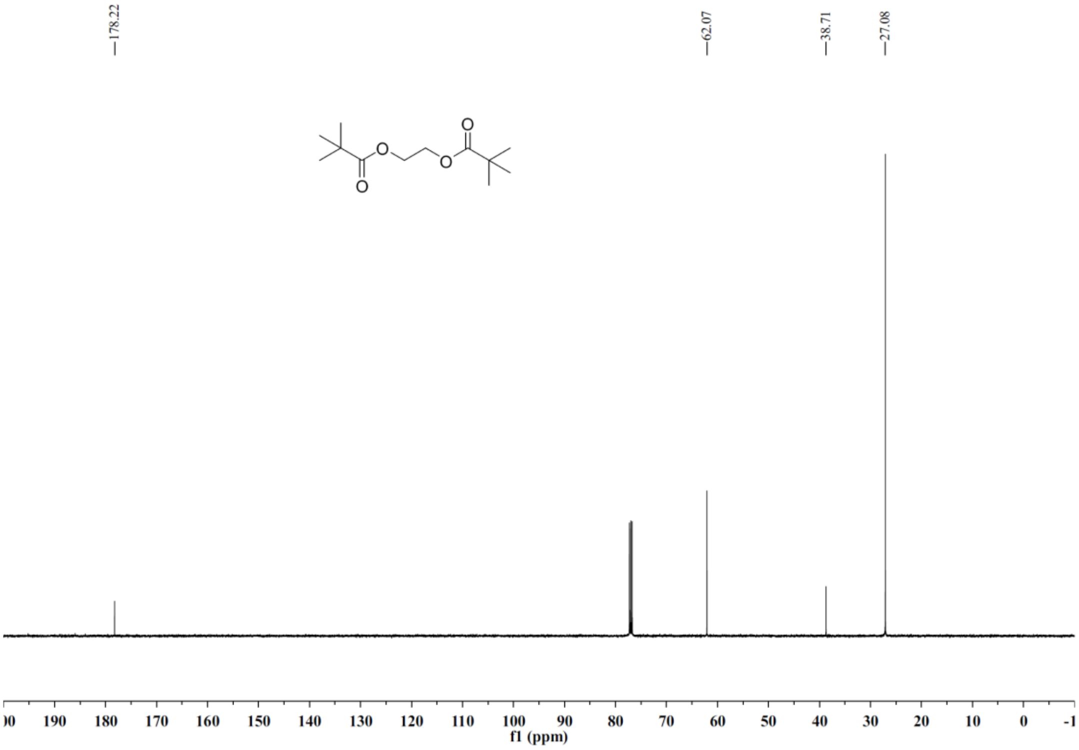

Supplementary Figure 4. NMR spectra of **1d**

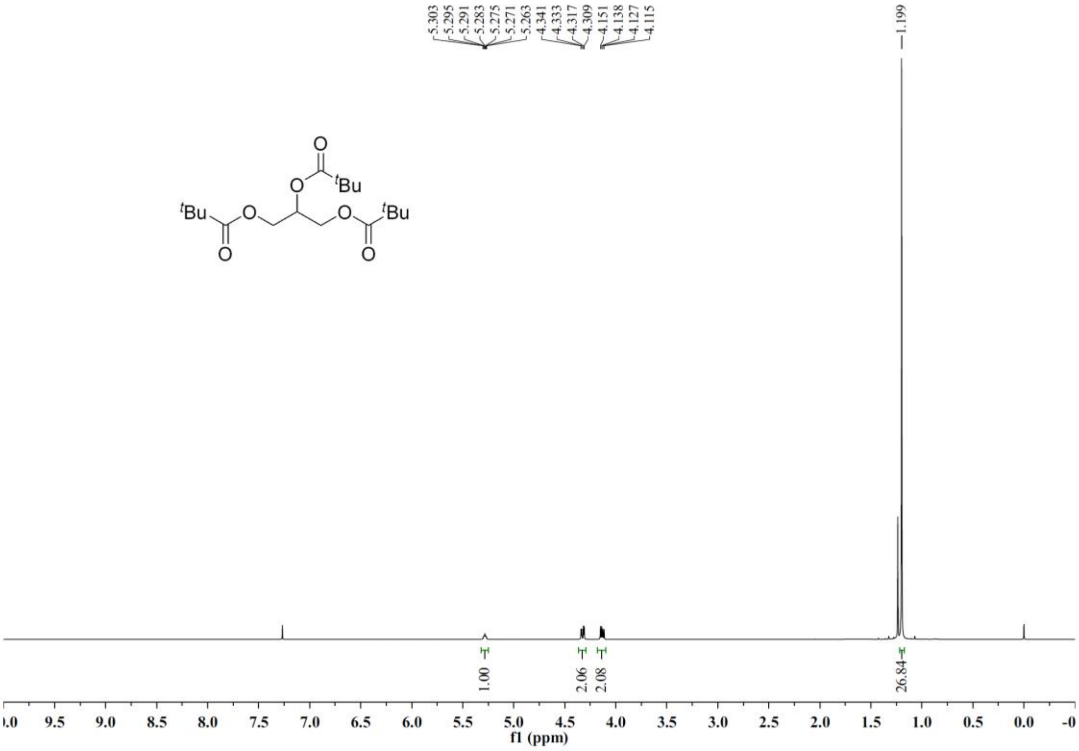

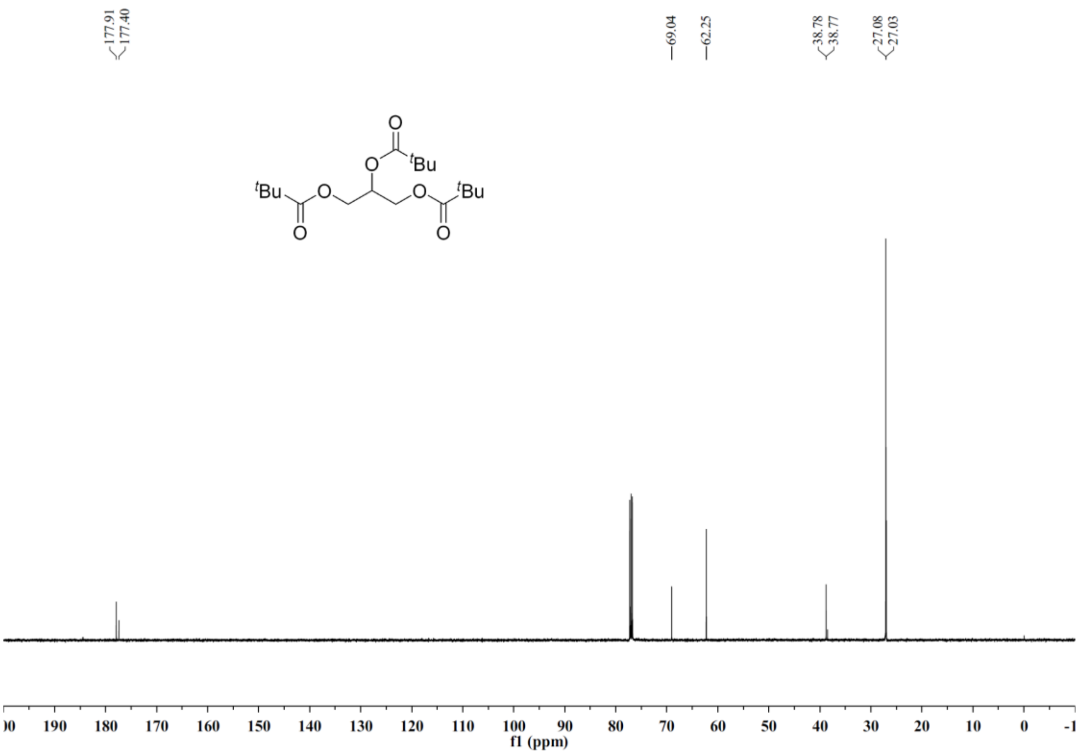

Supplementary Figure 5. NMR spectra of **1g**

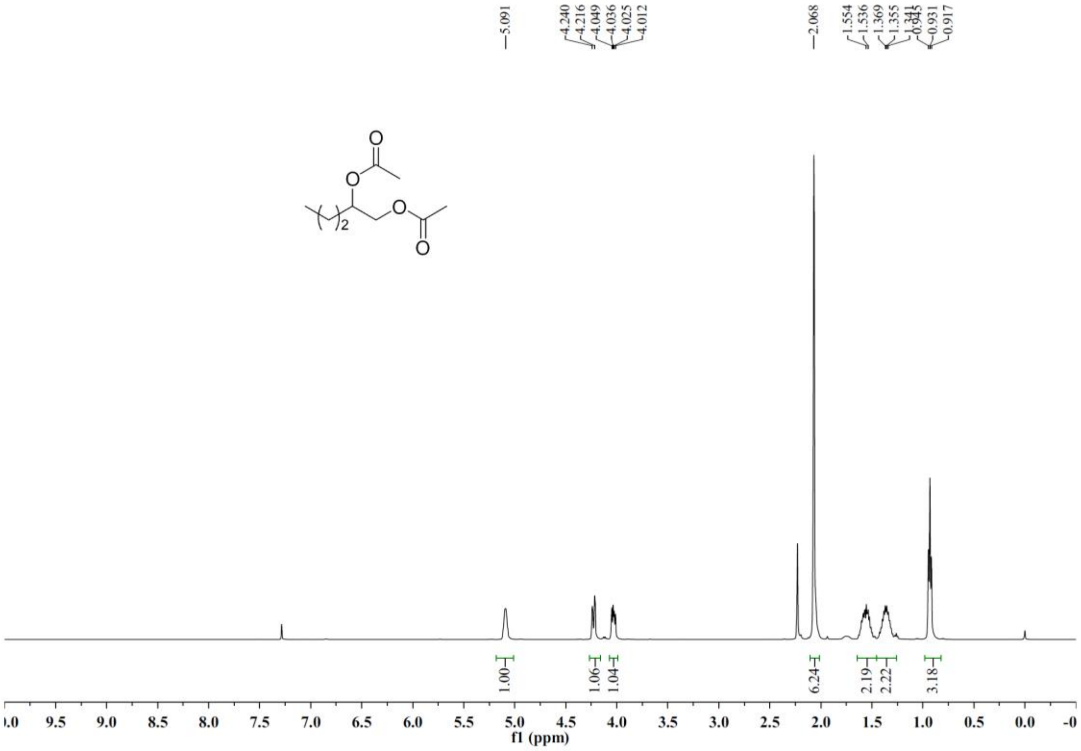

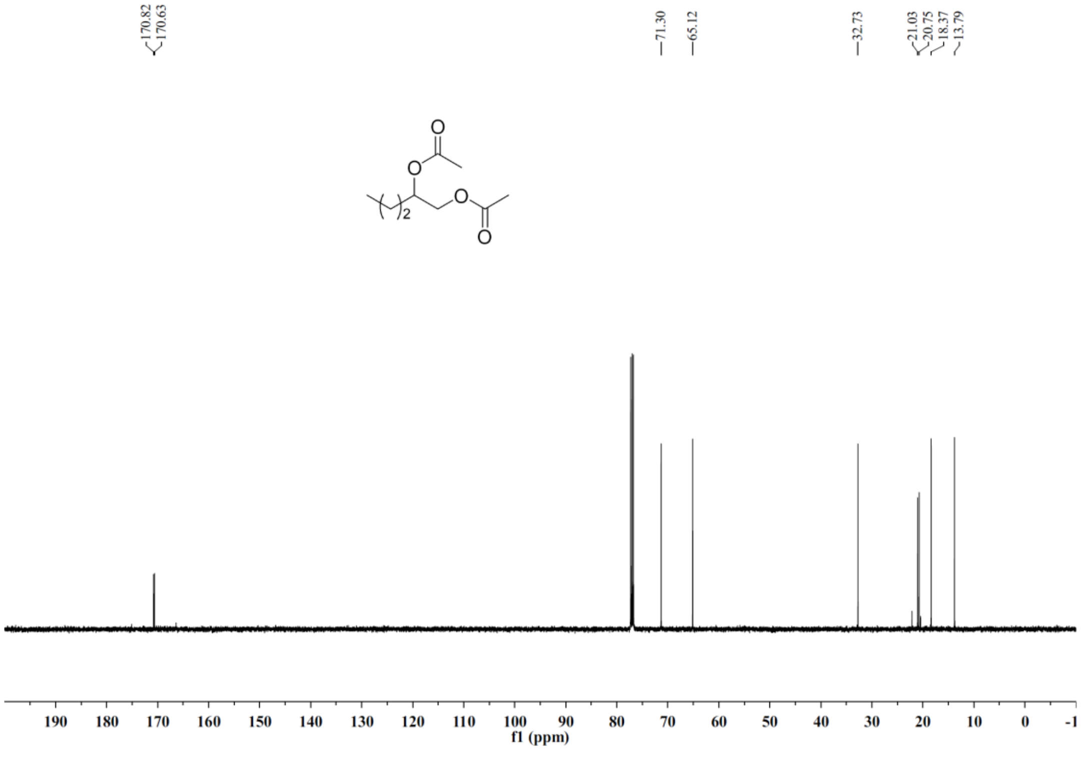

Supplementary Figure 6. NMR spectra of **1m**

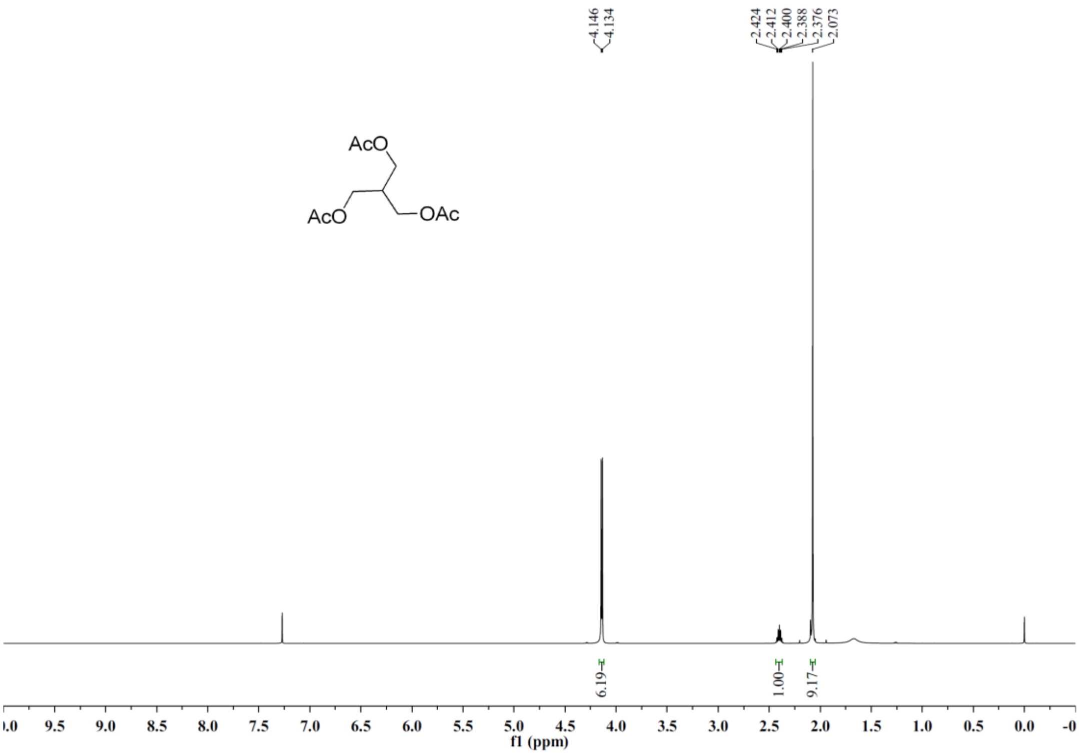

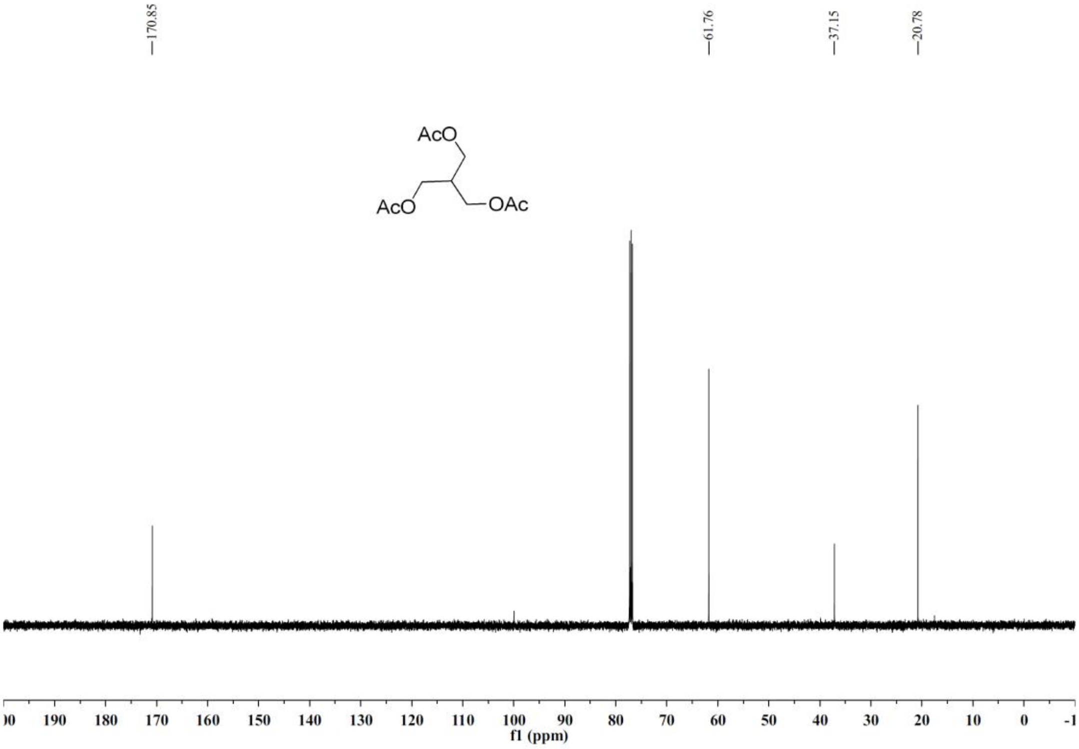

Supplementary Figure 7. NMR spectra of **1t**

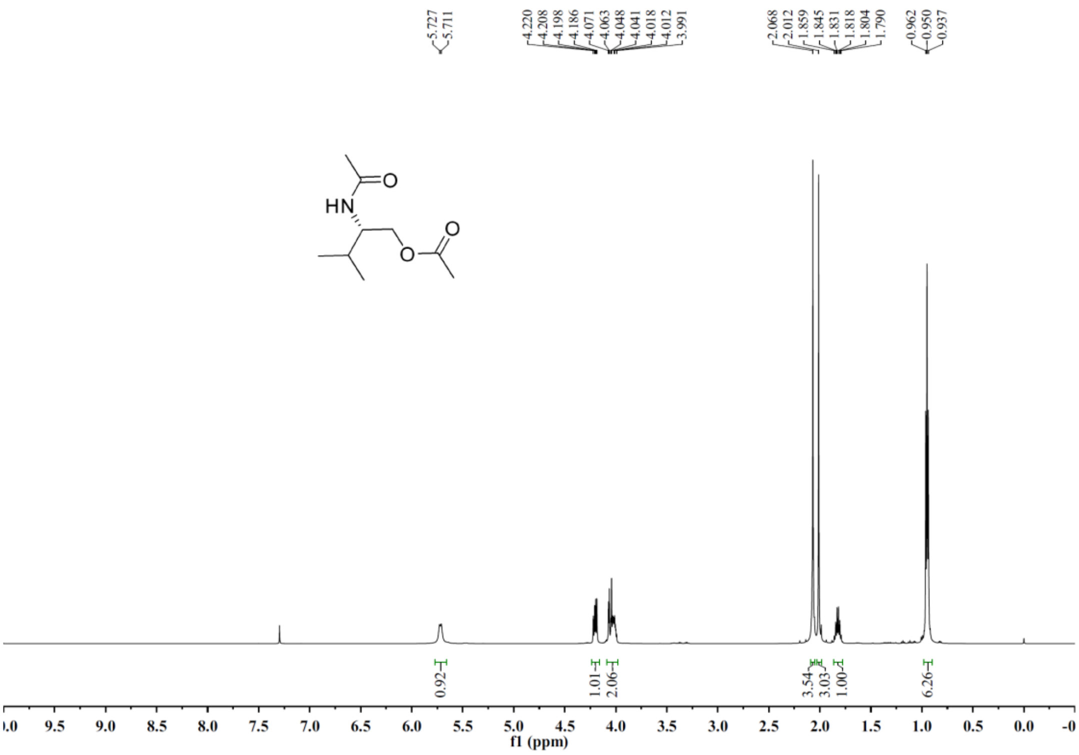

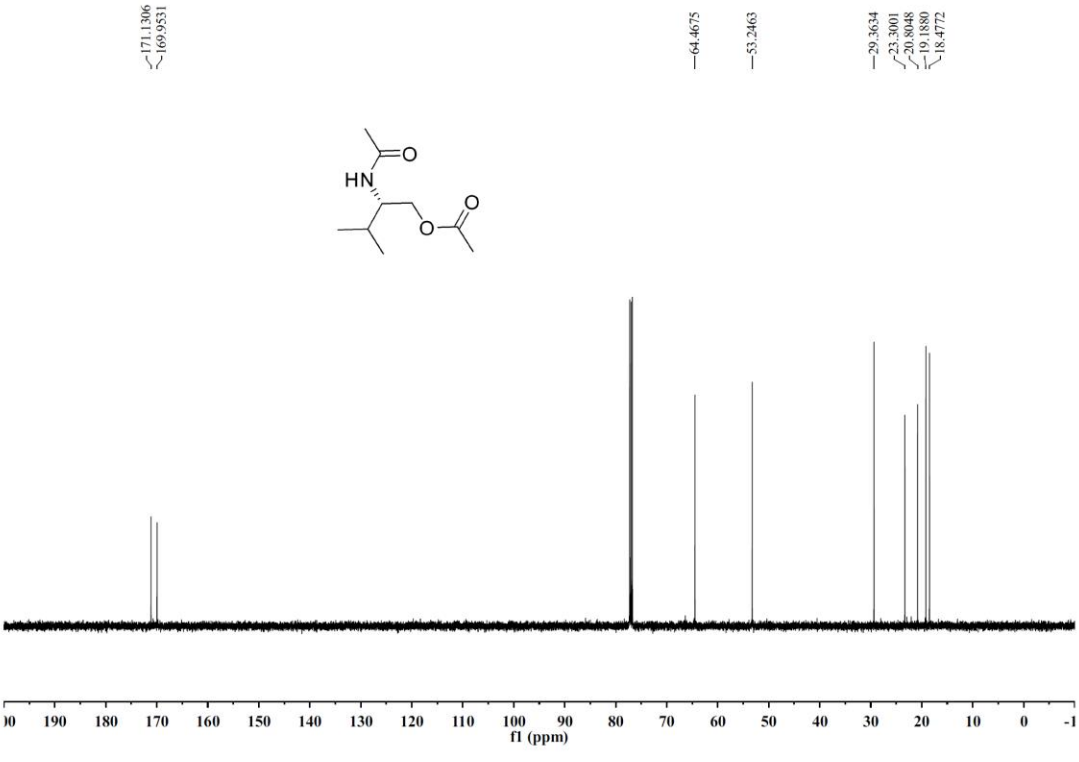

Supplementary Figure 8. NMR spectra of **4c**

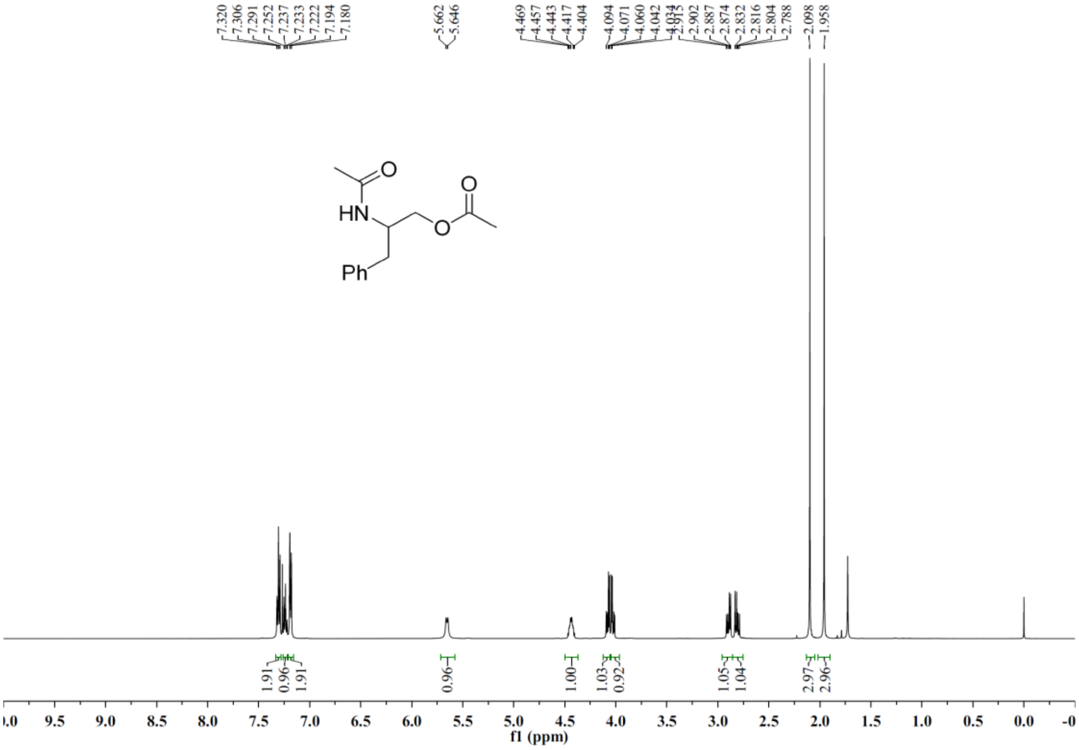

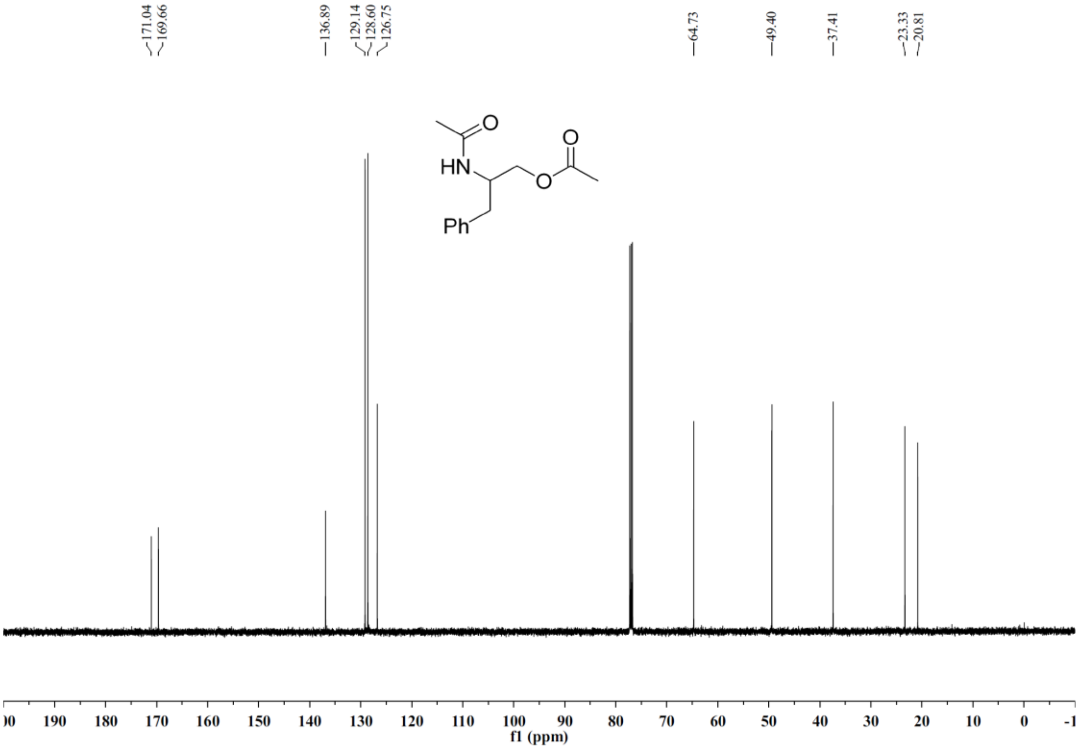

Supplementary Figure 9. NMR spectra of **4e**

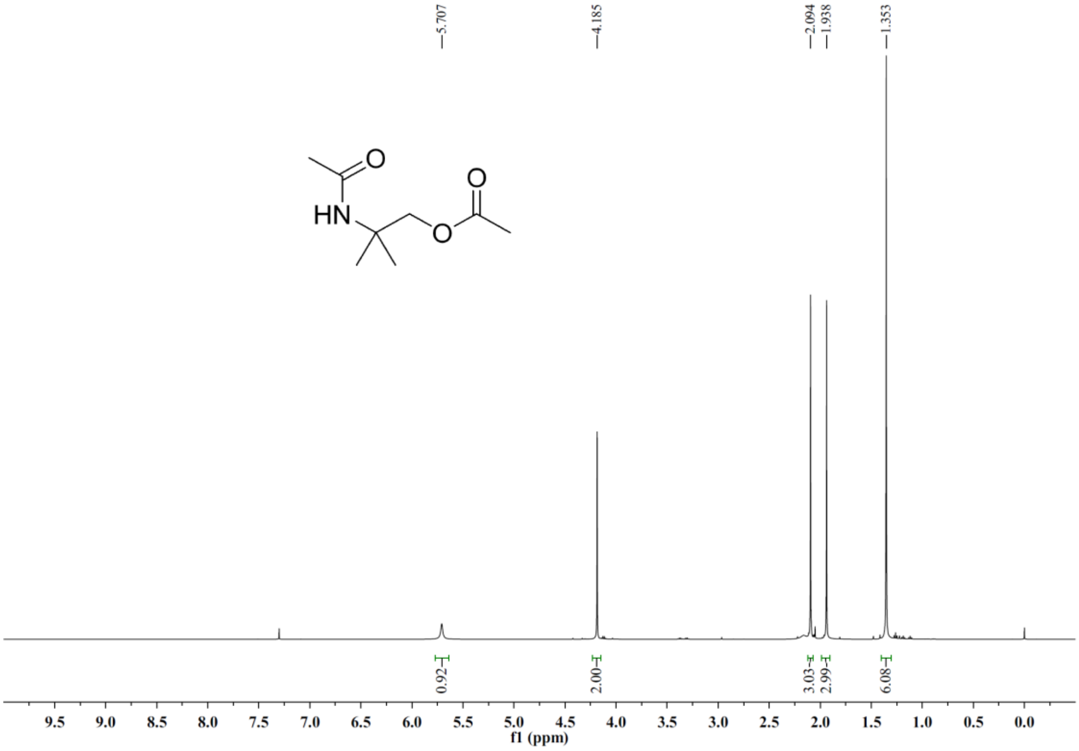

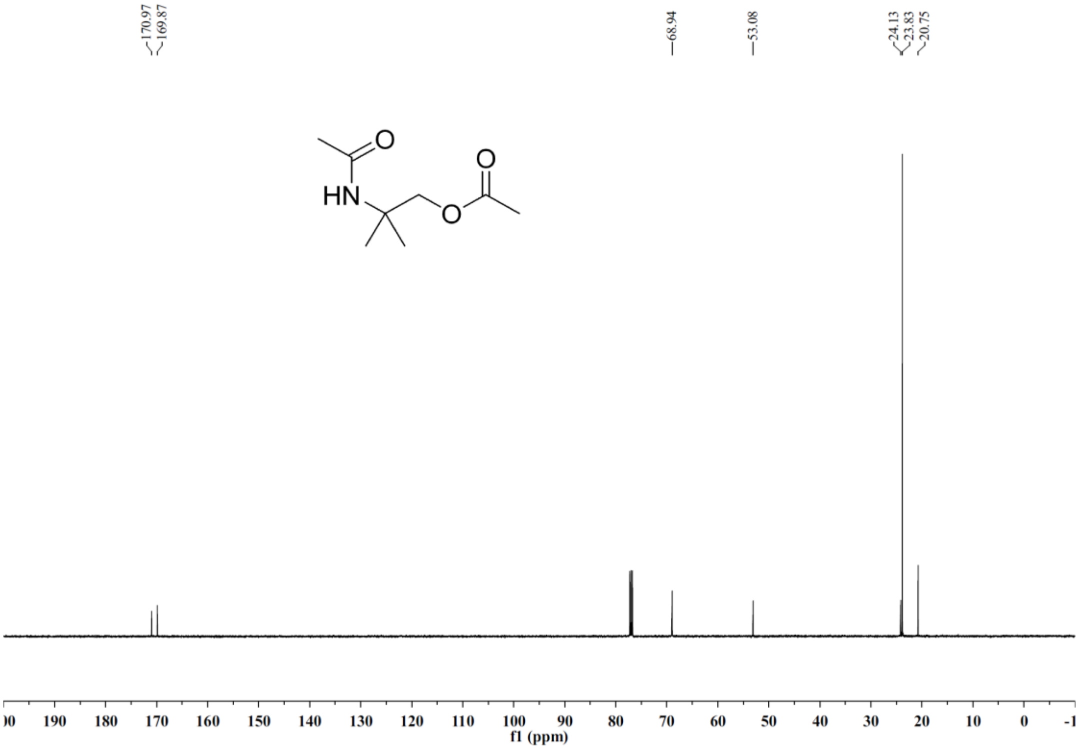

Supplementary Figure 10. NMR spectra of **4f**

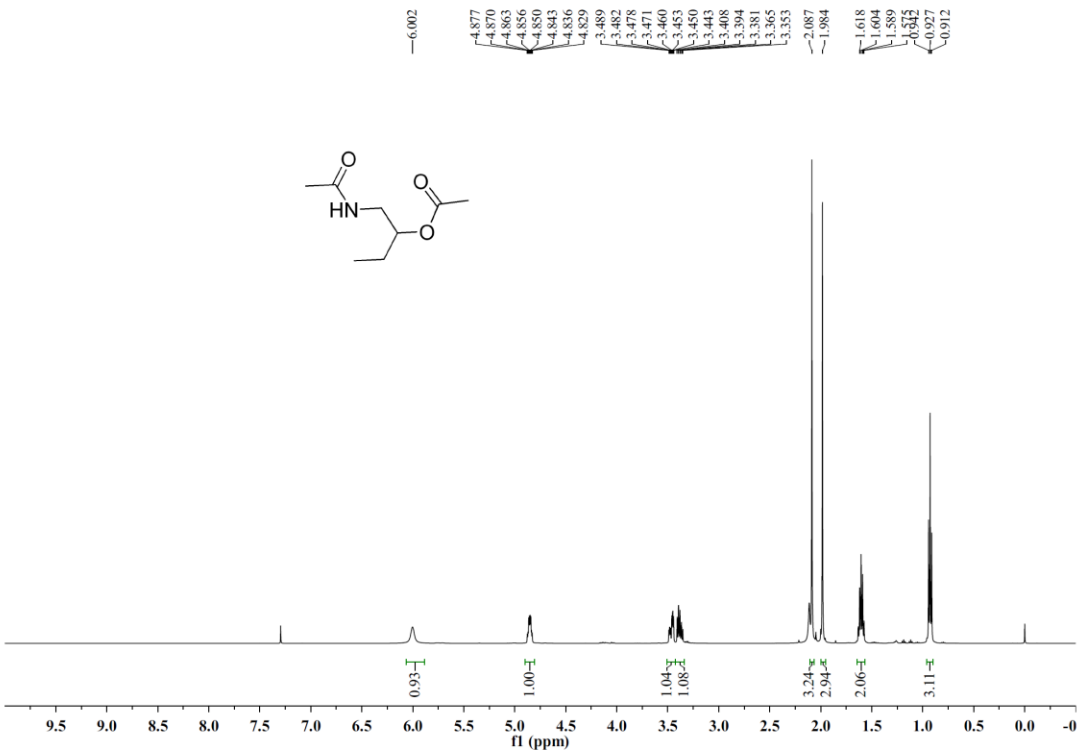

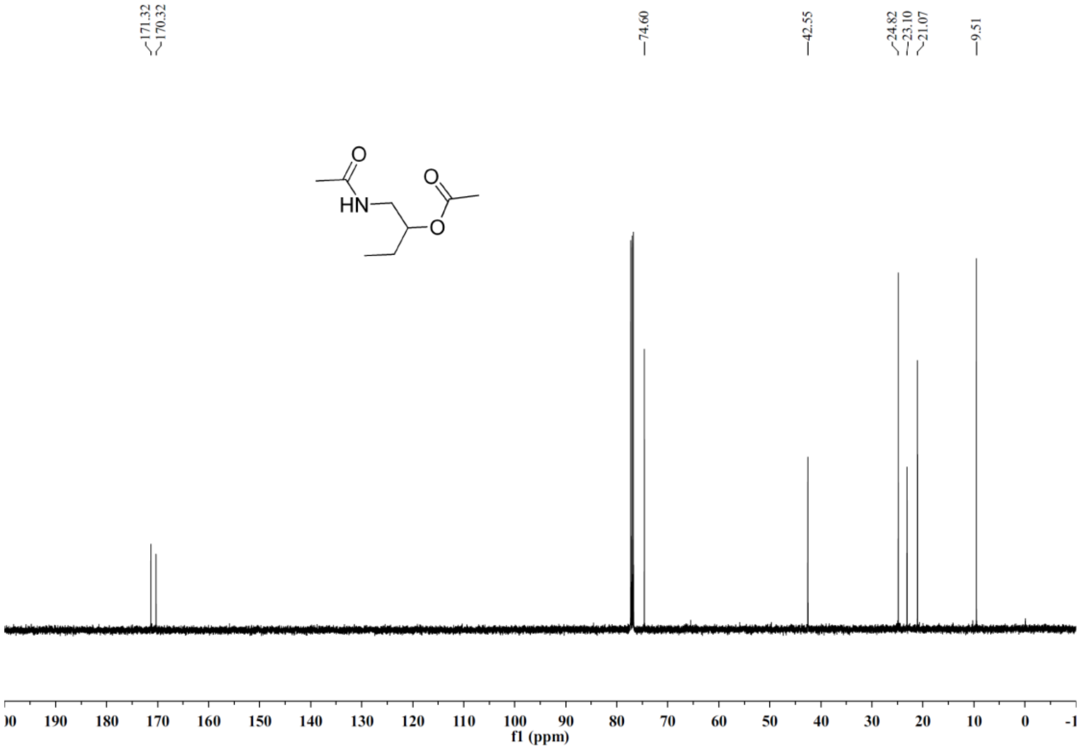

Supplementary Figure 11. NMR spectra of **4g**

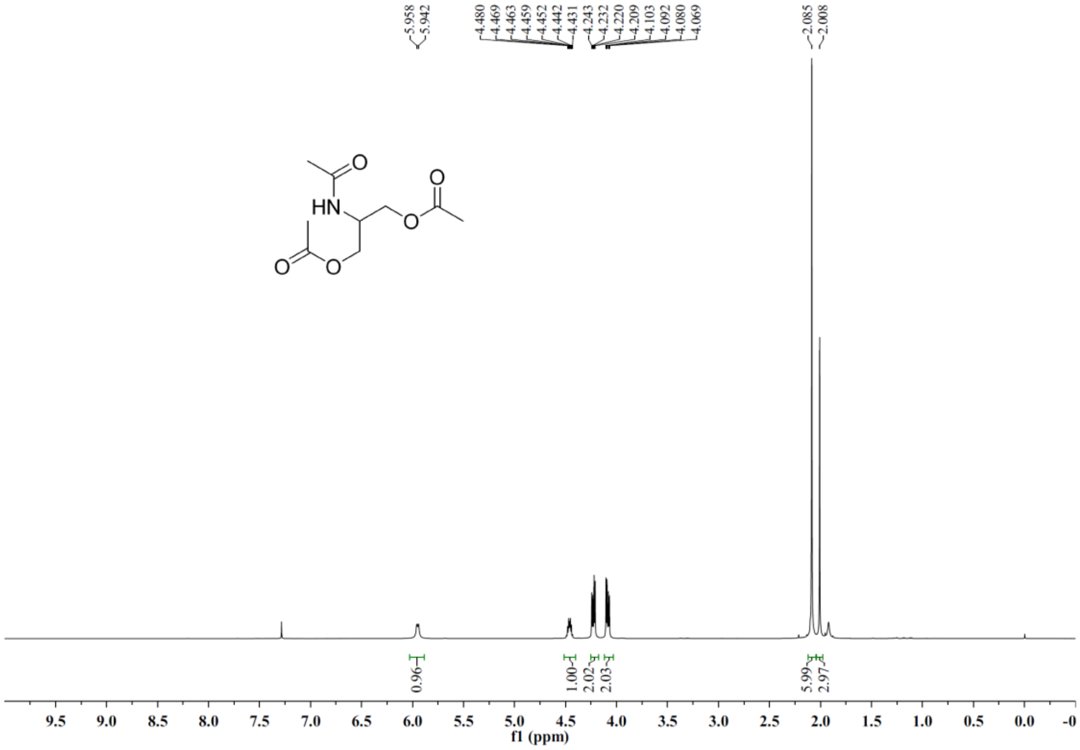

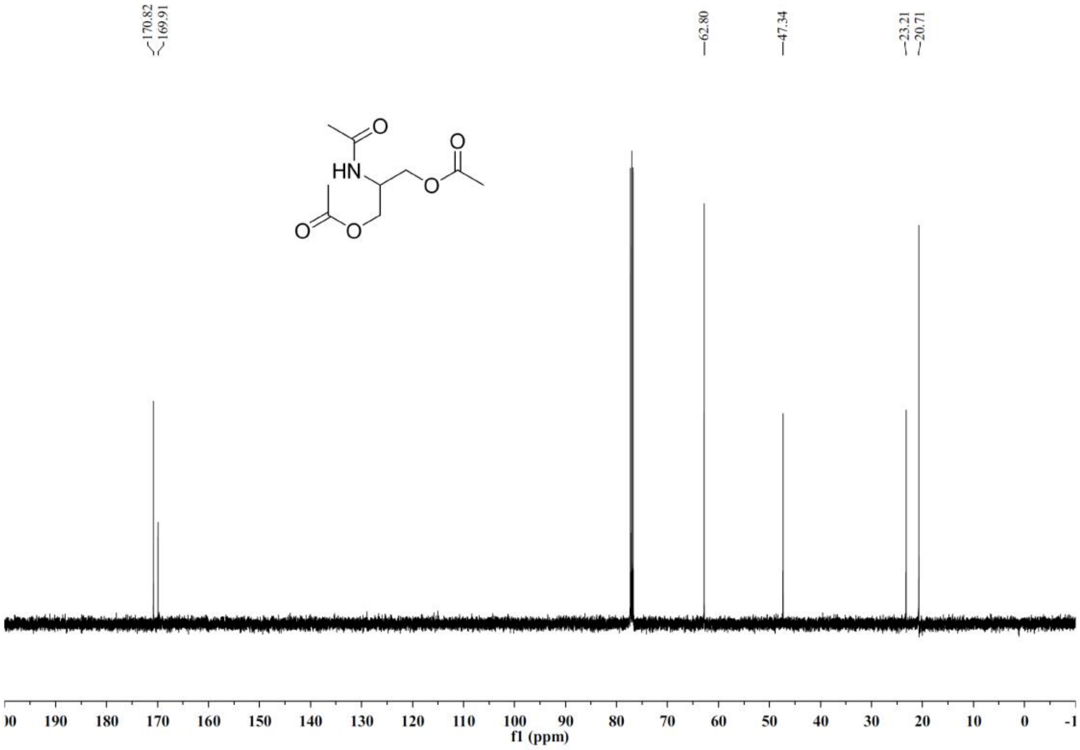

Supplementary Figure 12. NMR spectra of **4h**

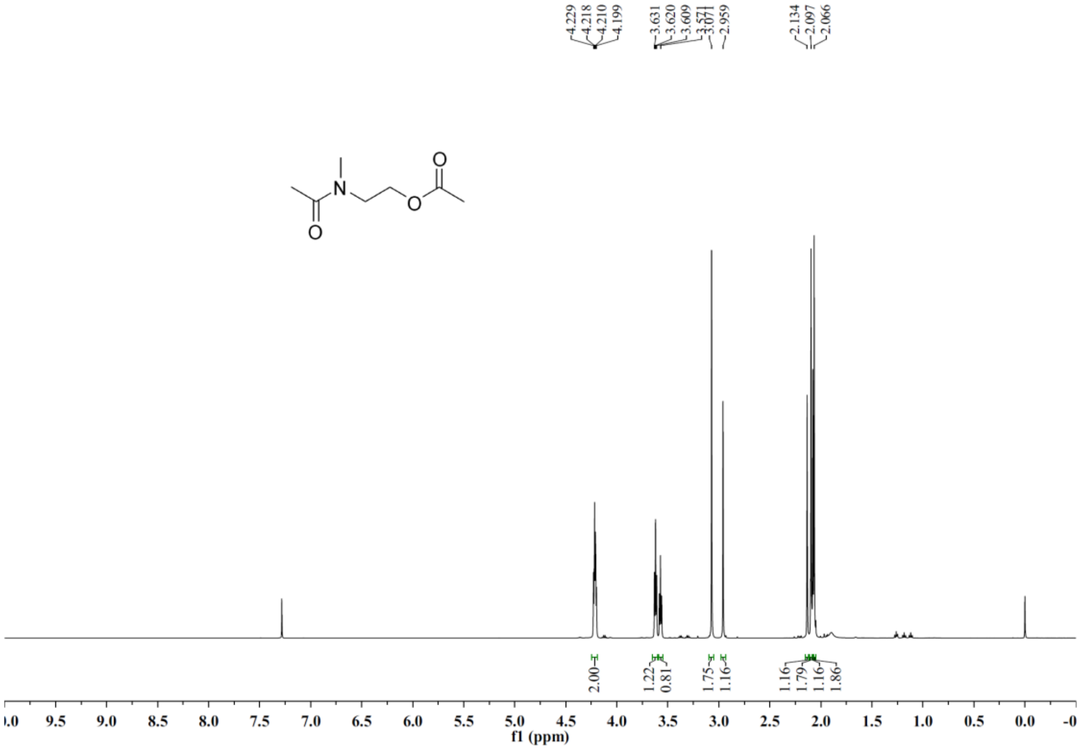

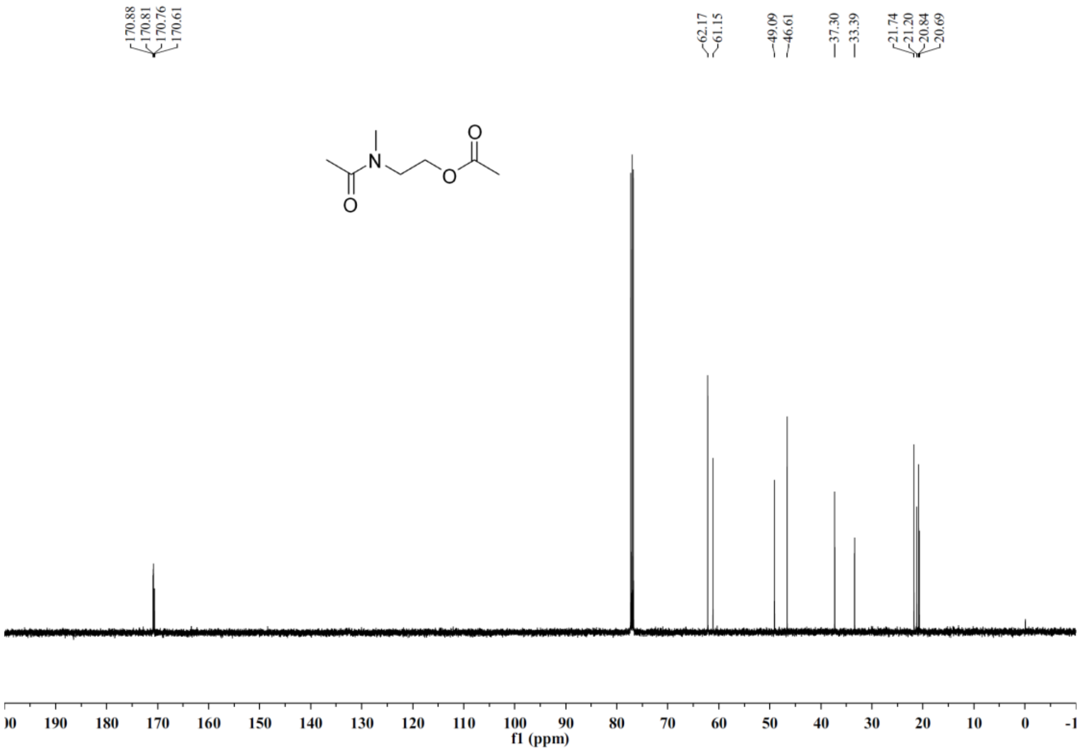

Supplementary Figure 13. NMR spectra of **4i**

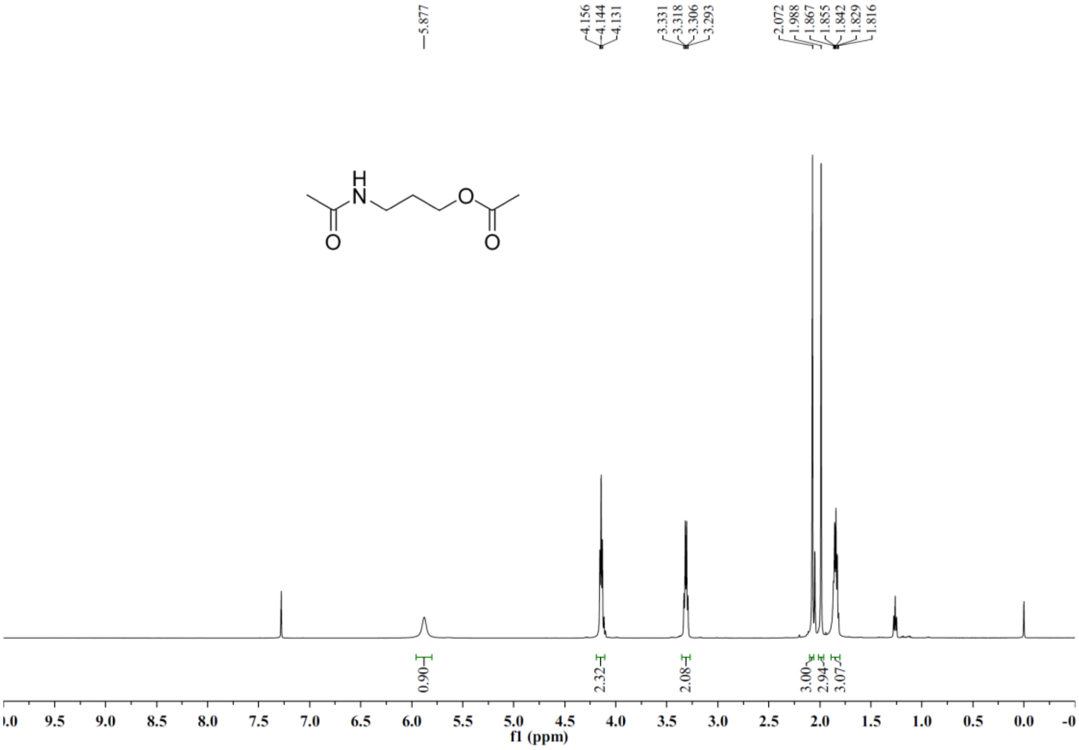

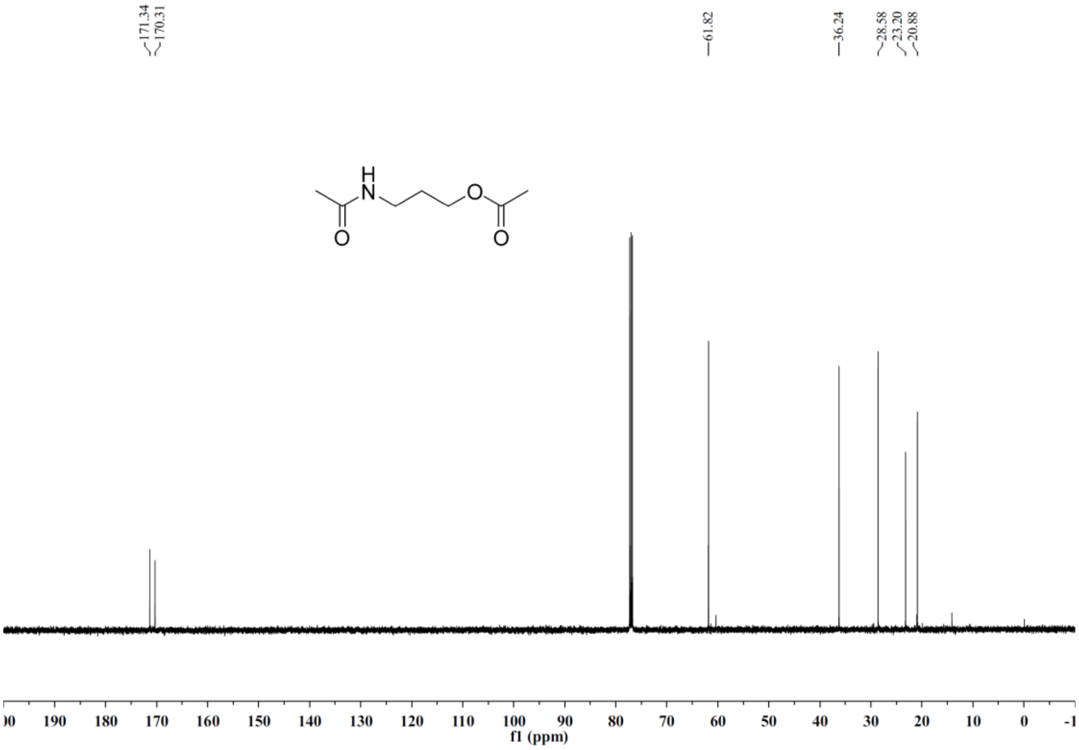

Supplementary Figure 14. NMR spectra of **4k**

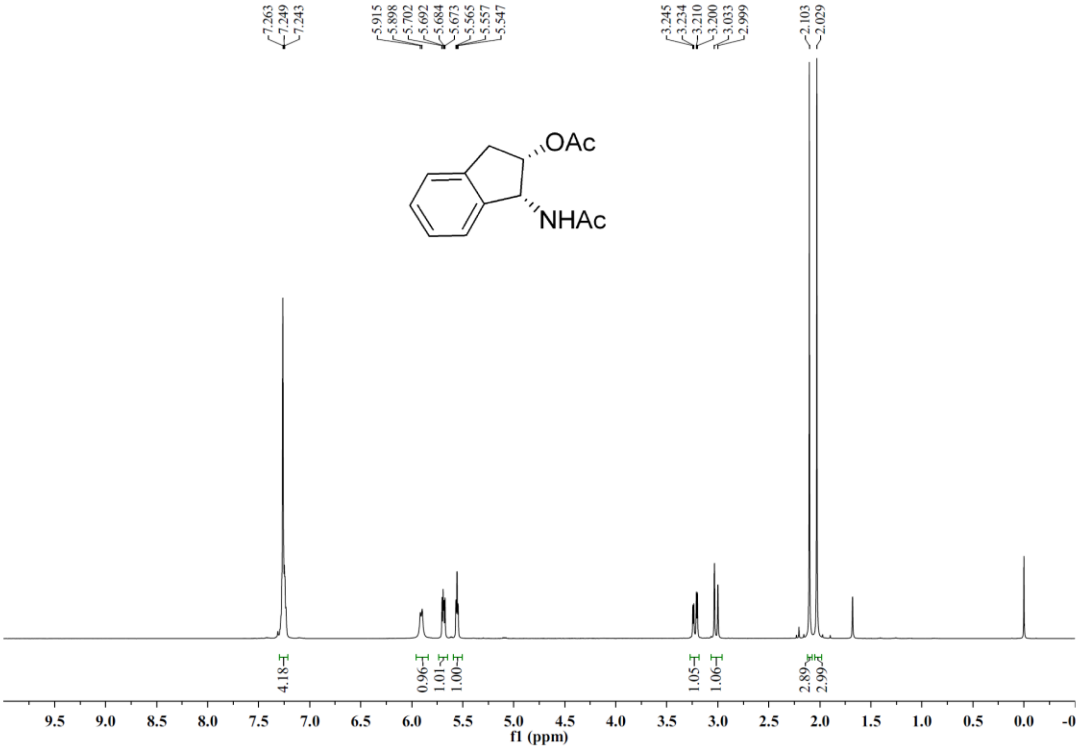

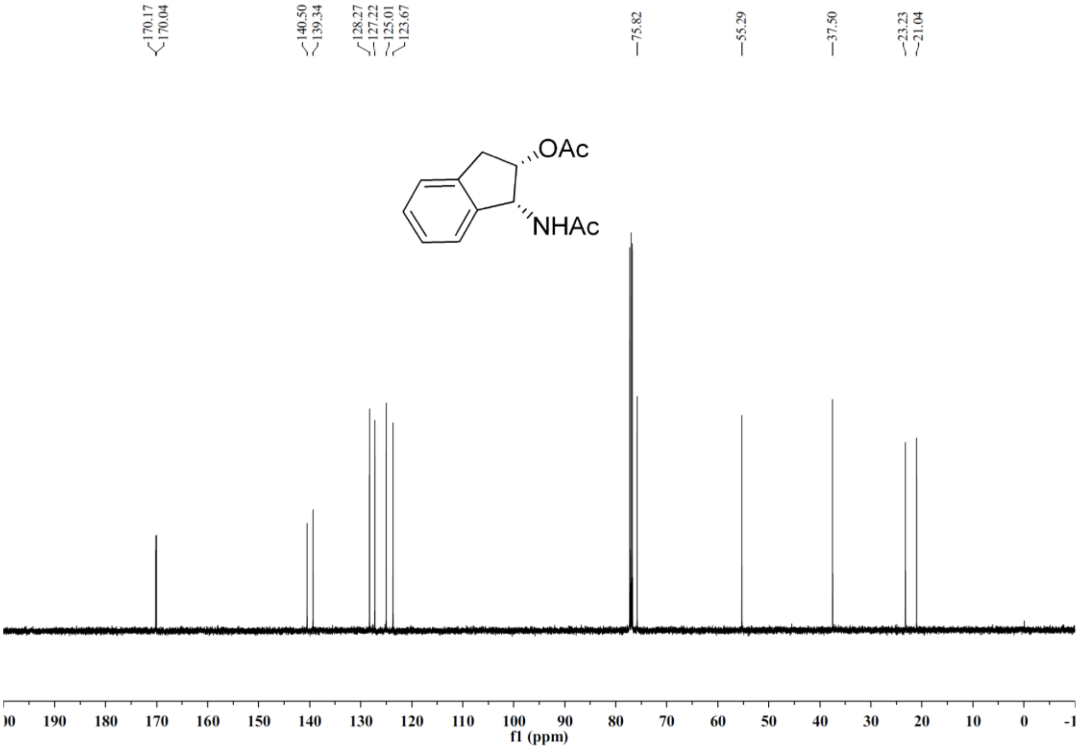

Supplementary Figure 15. NMR spectra of **4l**

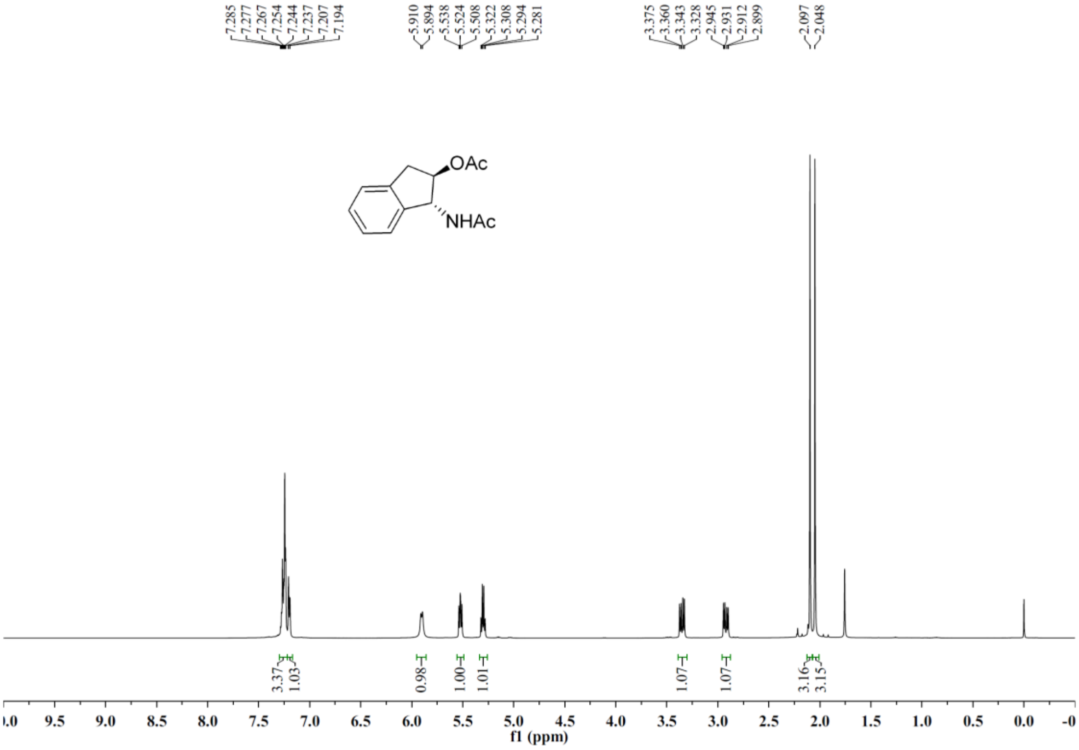

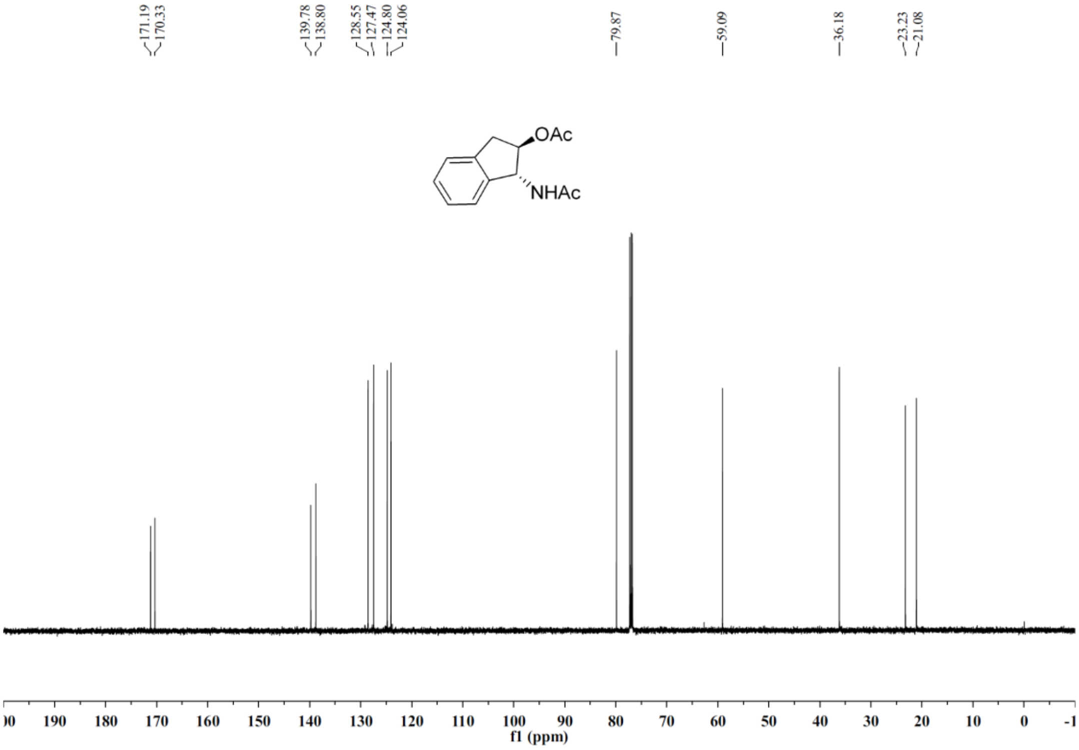

Supplementary Figure 16. NMR spectra of **4l'**

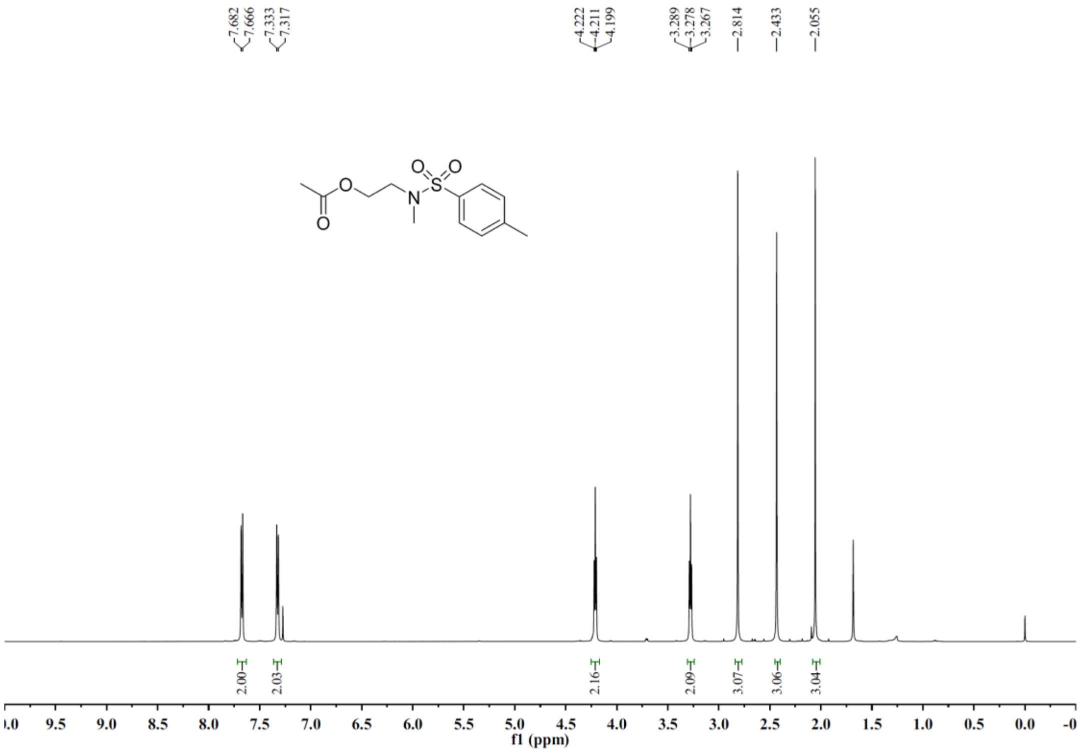

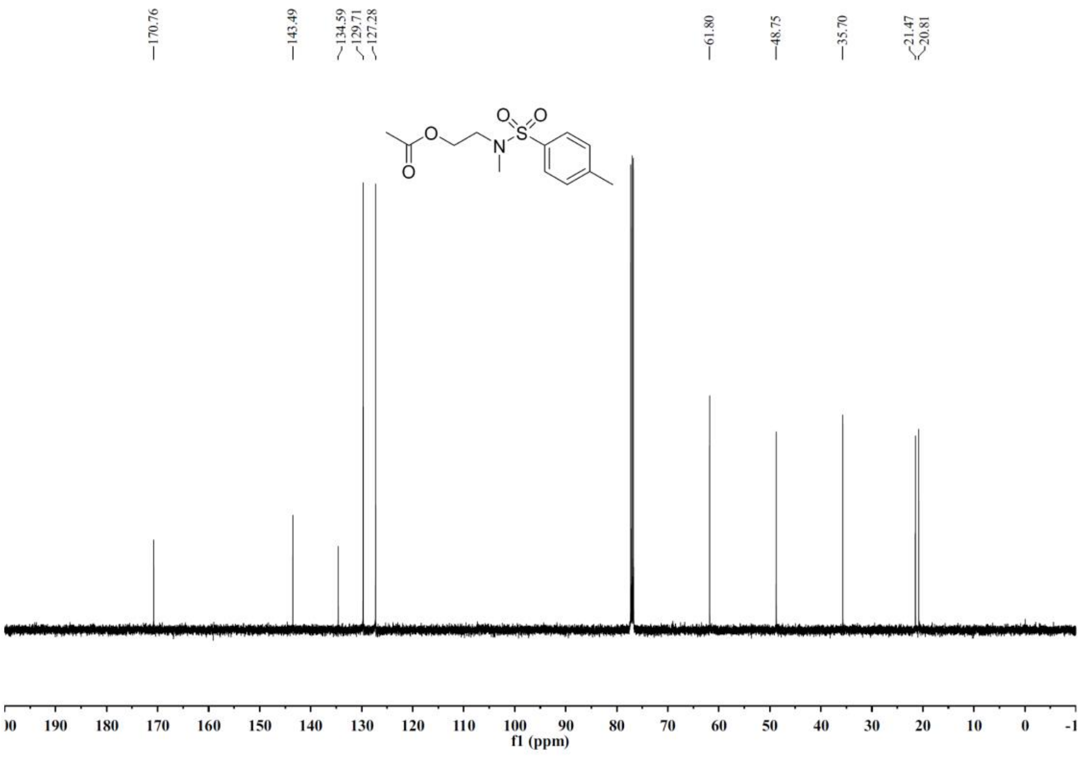

Supplementary Figure 17. NMR spectra of **3aa**

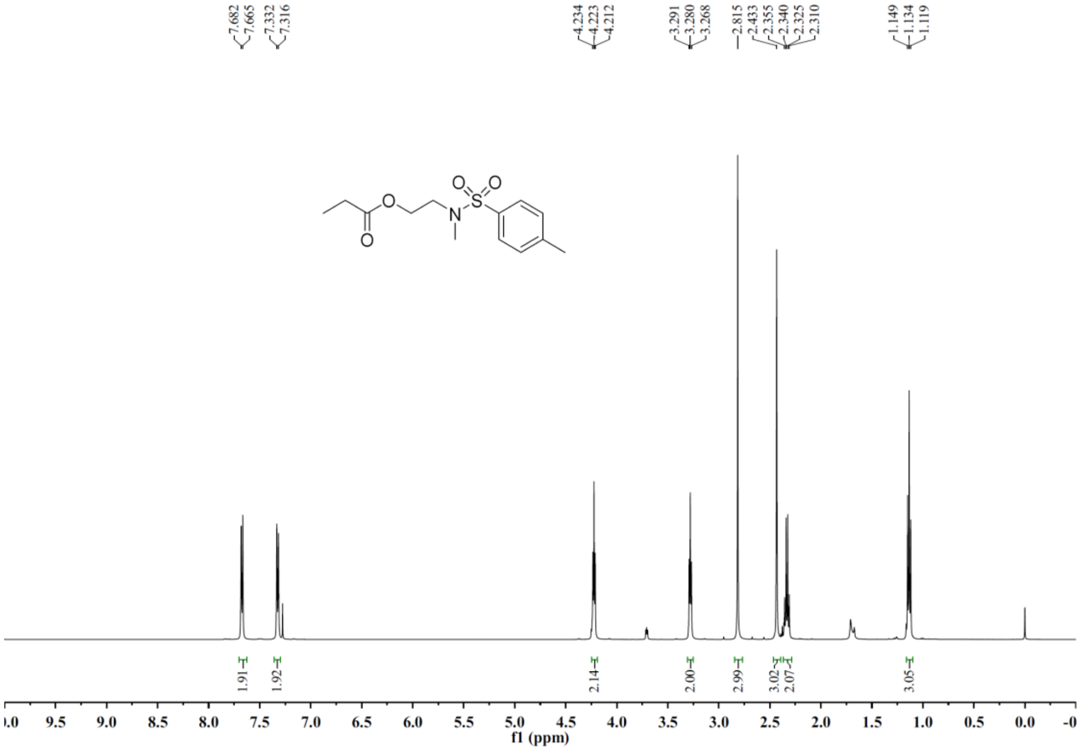

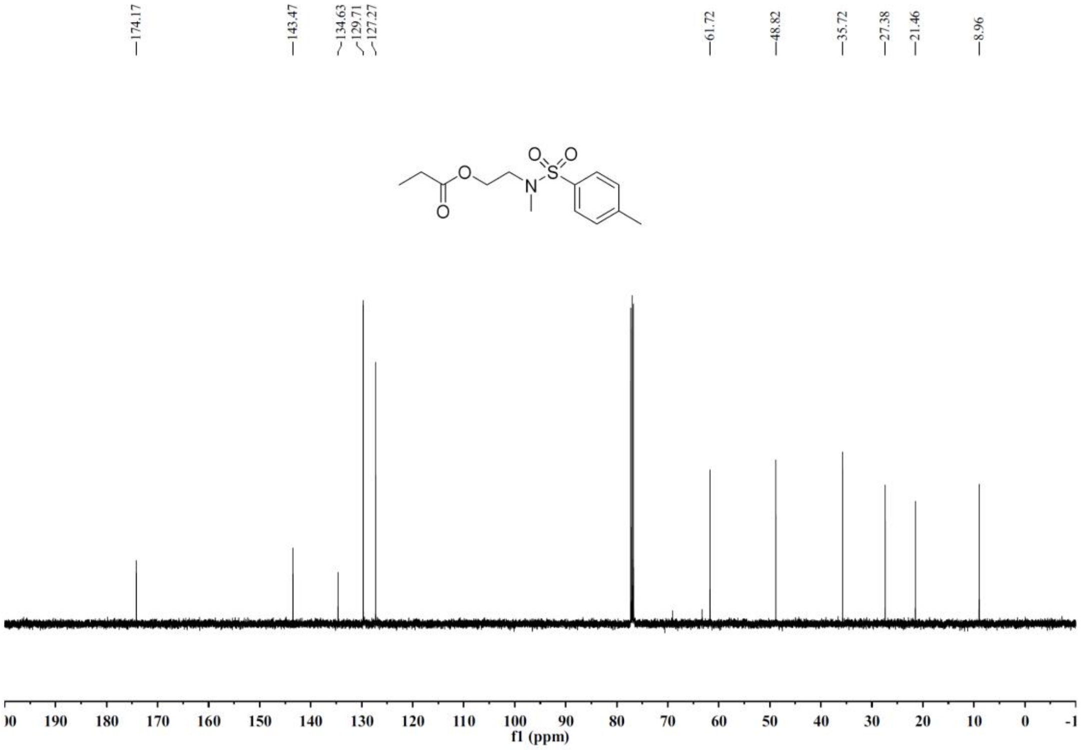

Supplementary Figure 18. NMR spectra of **3ba**

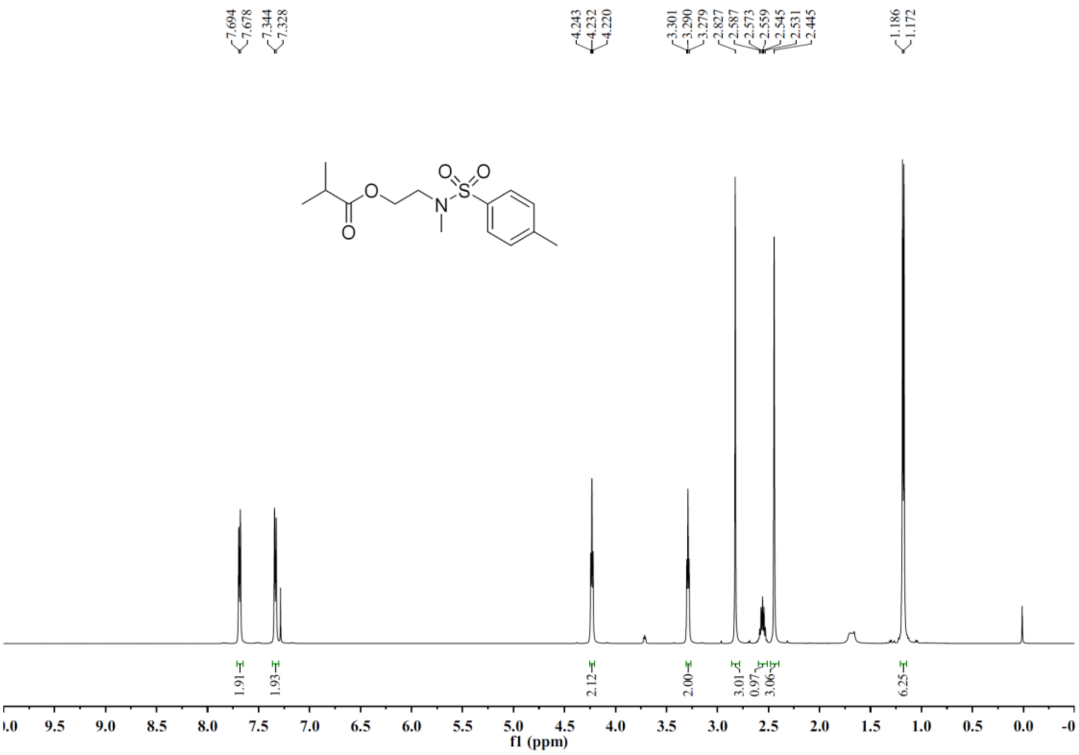

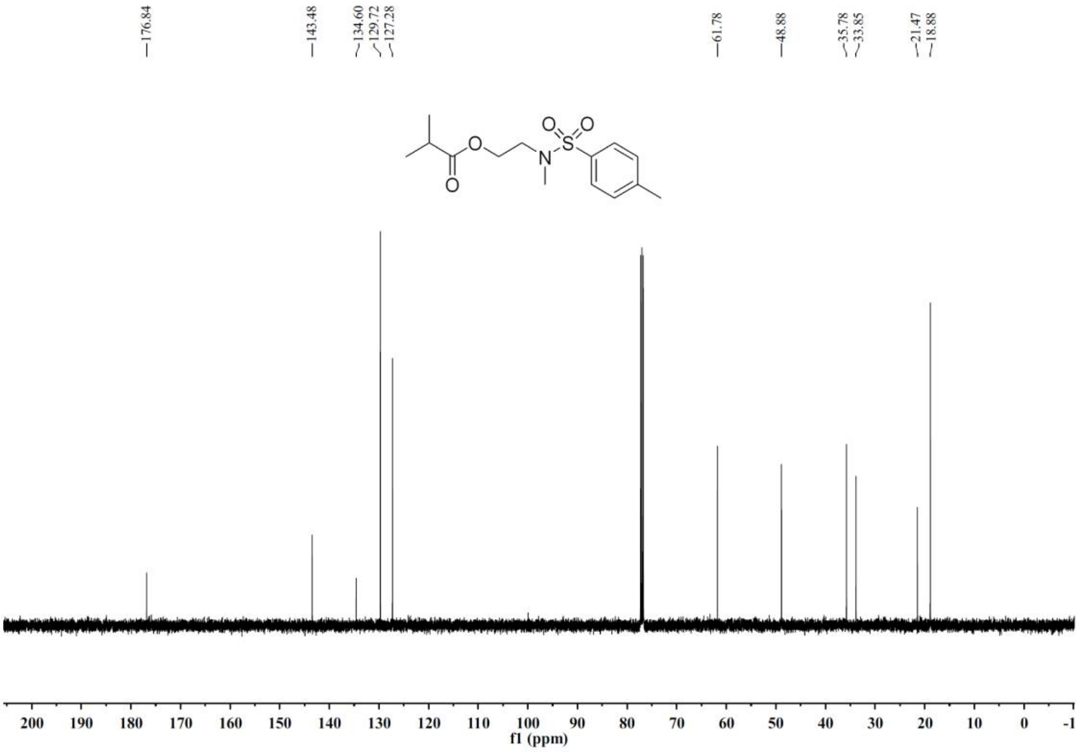

Supplementary Figure 19. NMR spectra of **3ca**

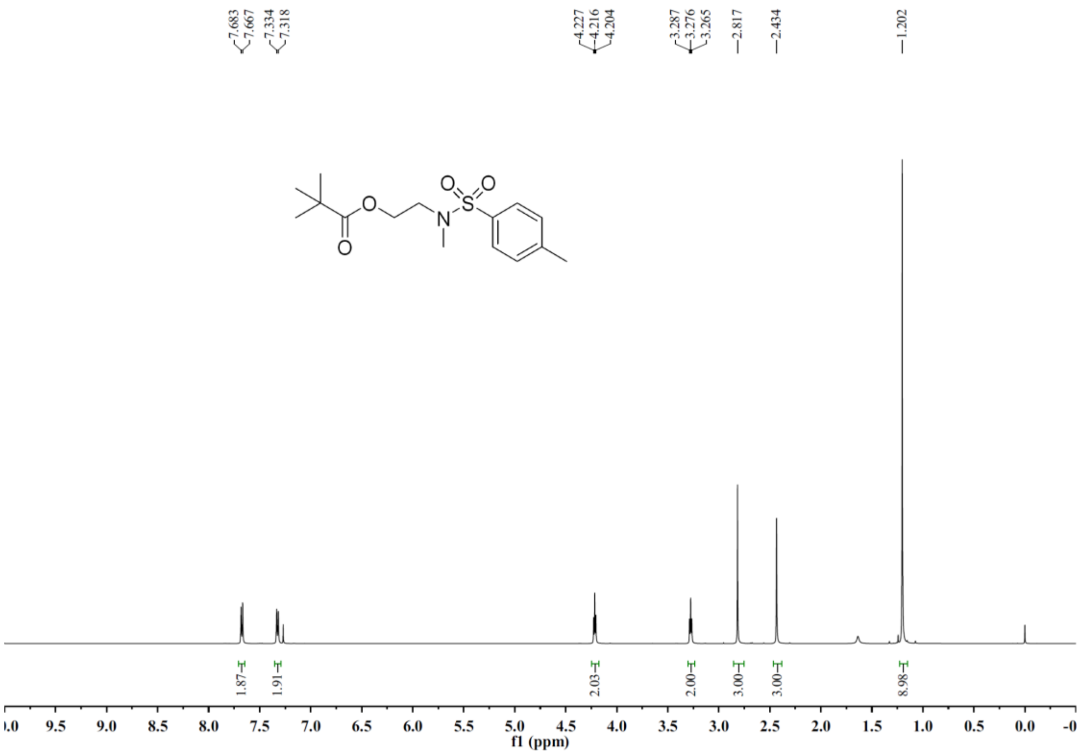

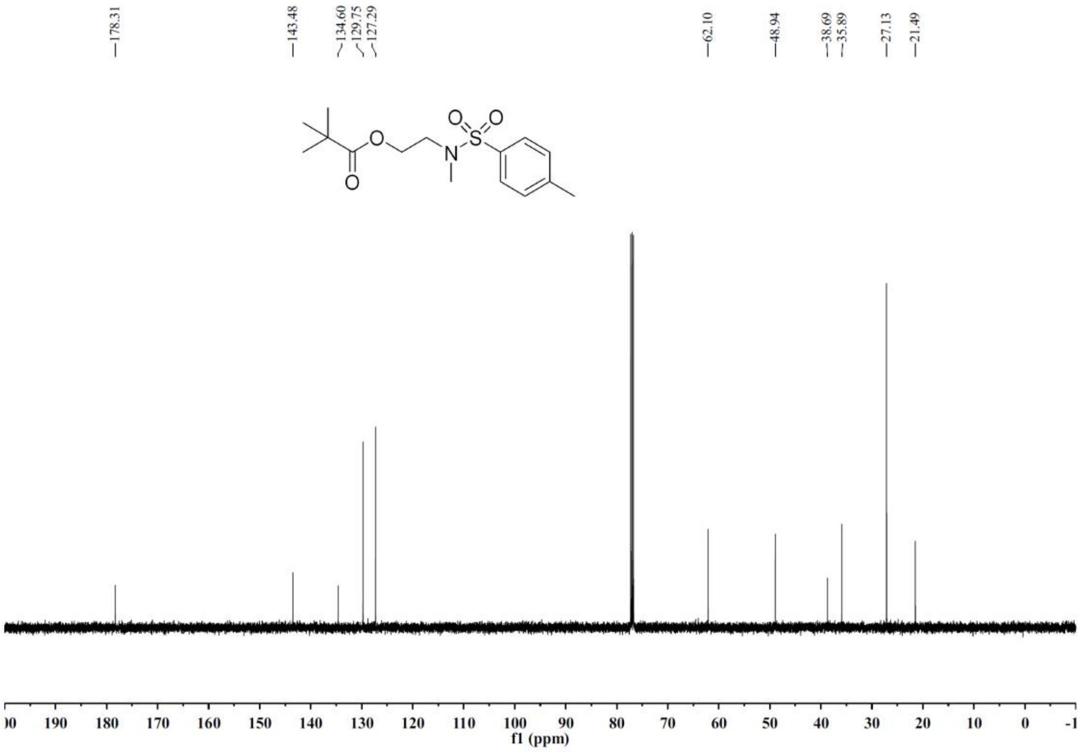

Supplementary Figure 20. NMR spectra of **3da**

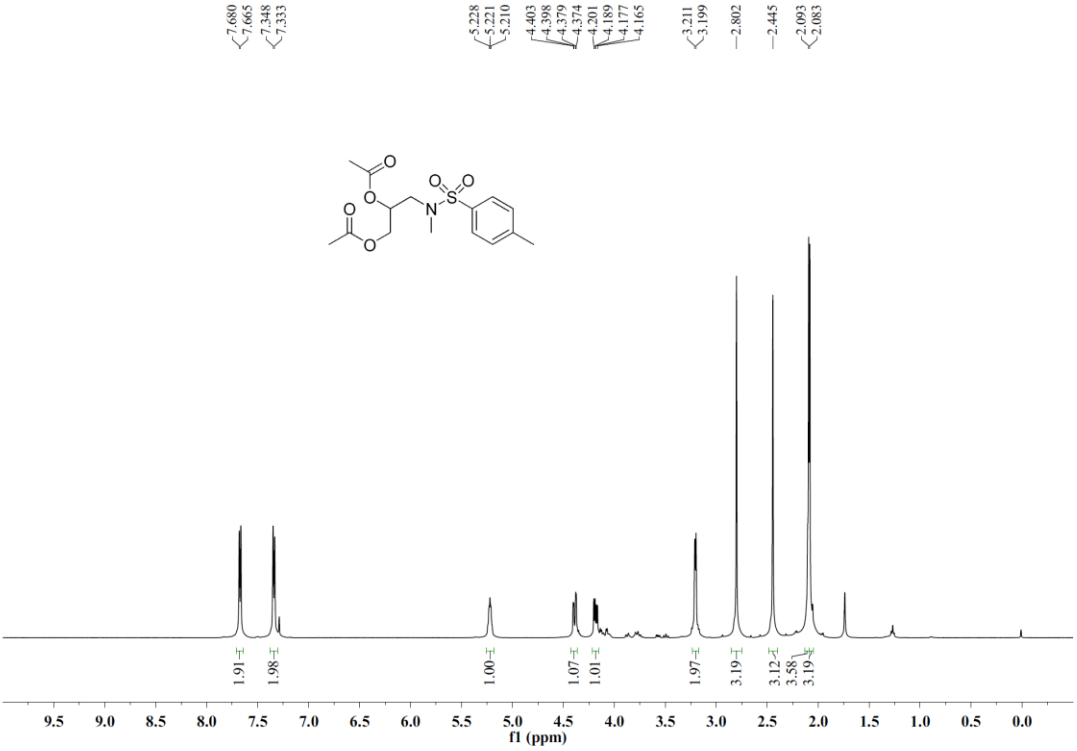

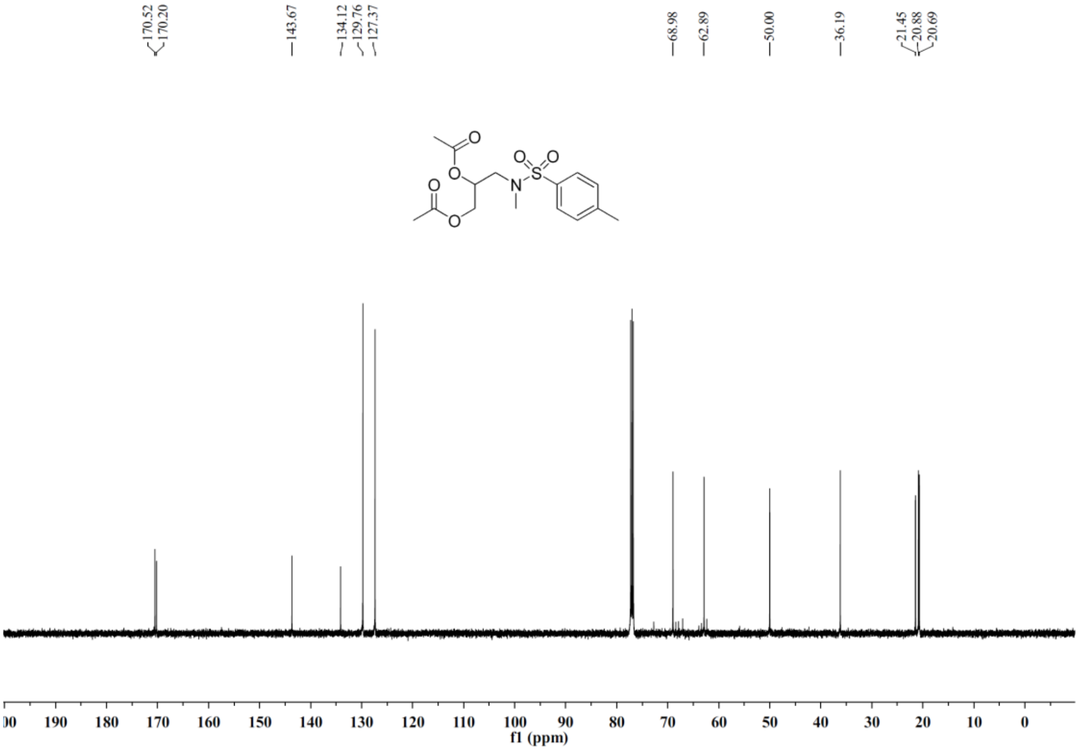

Supplementary Figure 21. NMR spectra of **3ea**

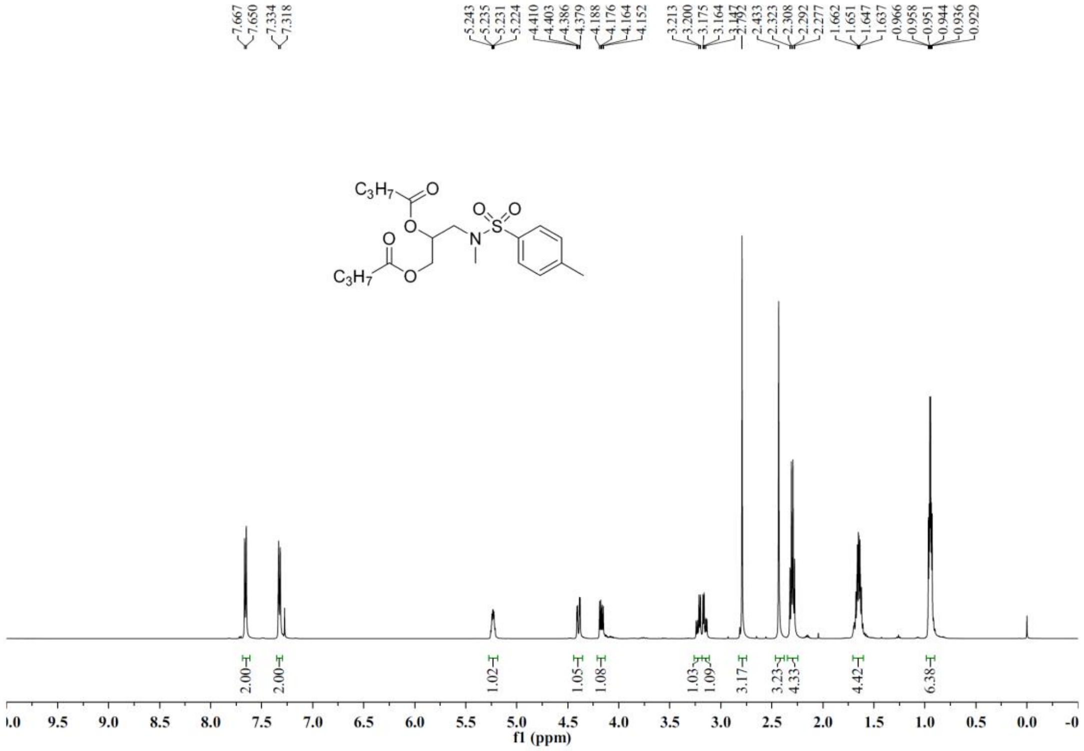

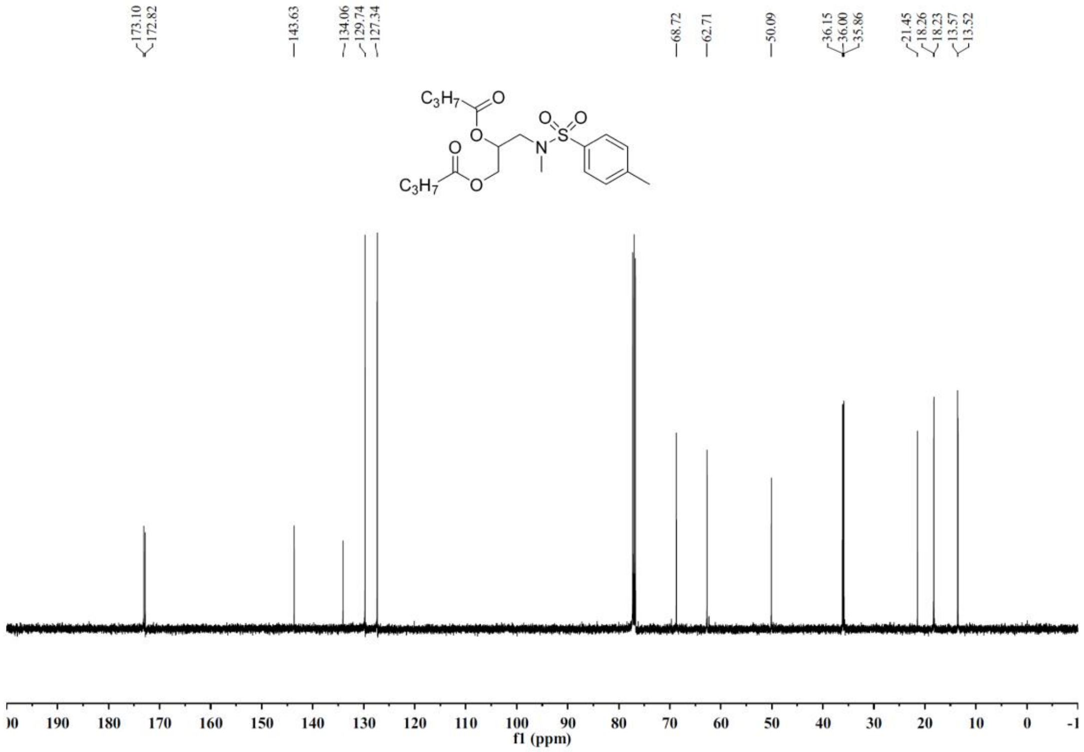

Supplementary Figure 22. NMR spectra of **3fa**

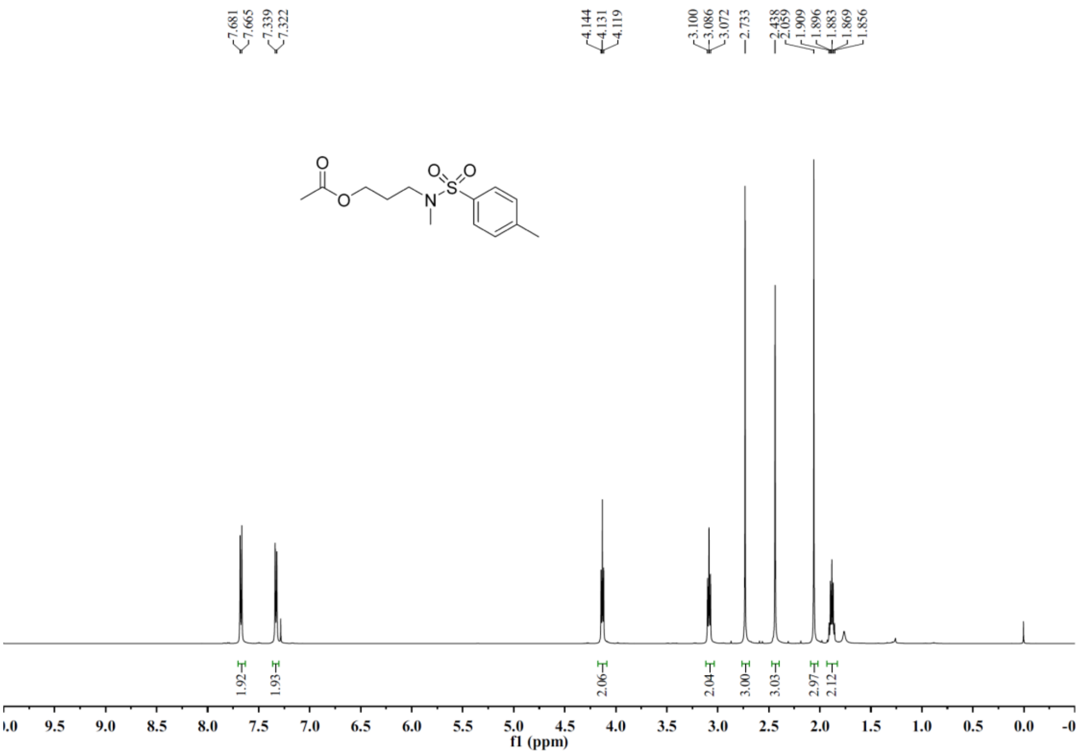

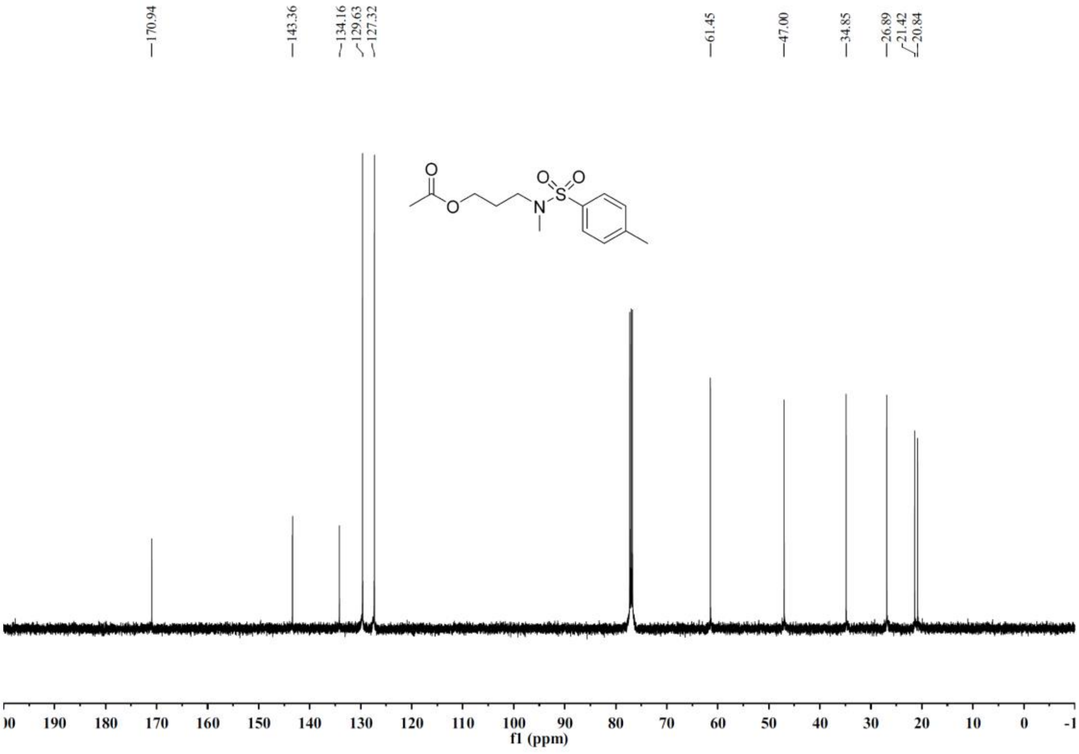

Supplementary Figure 23. NMR spectra of **3sa**

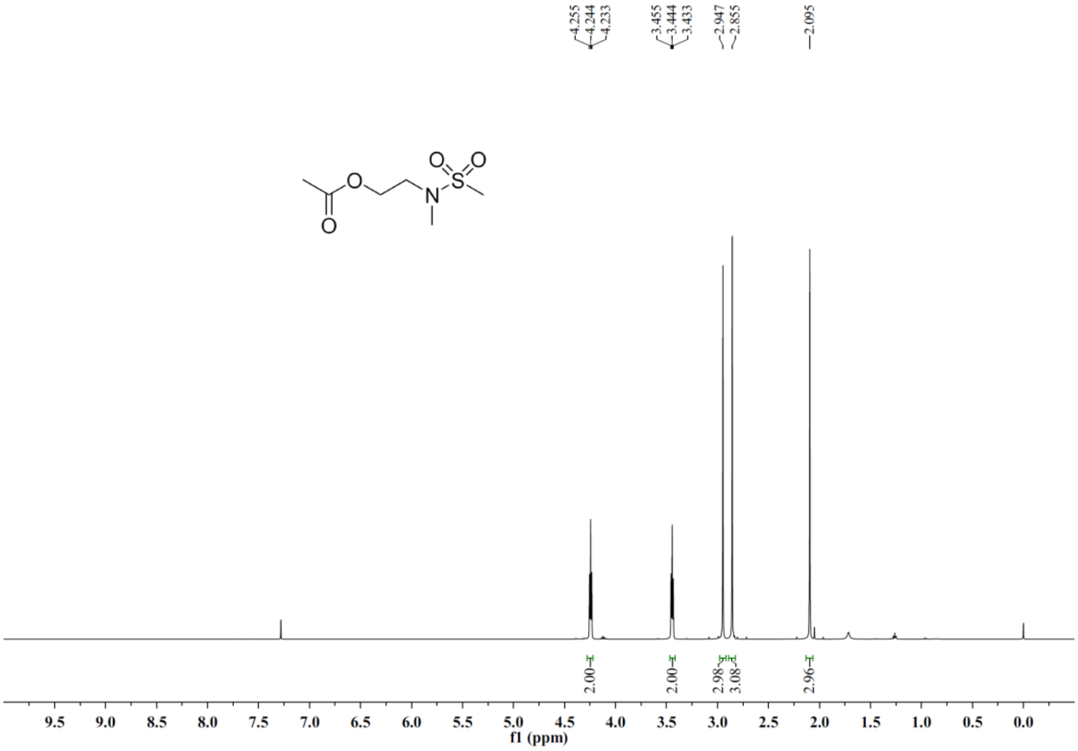

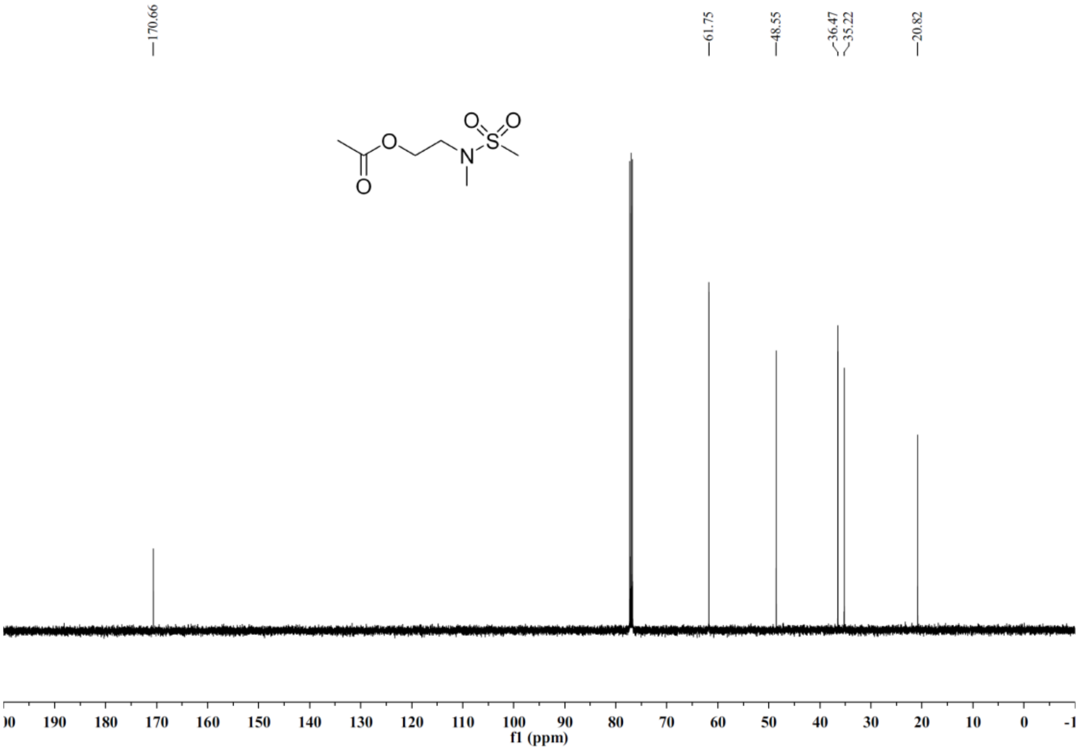

Supplementary Figure 24. NMR spectra of **3af**

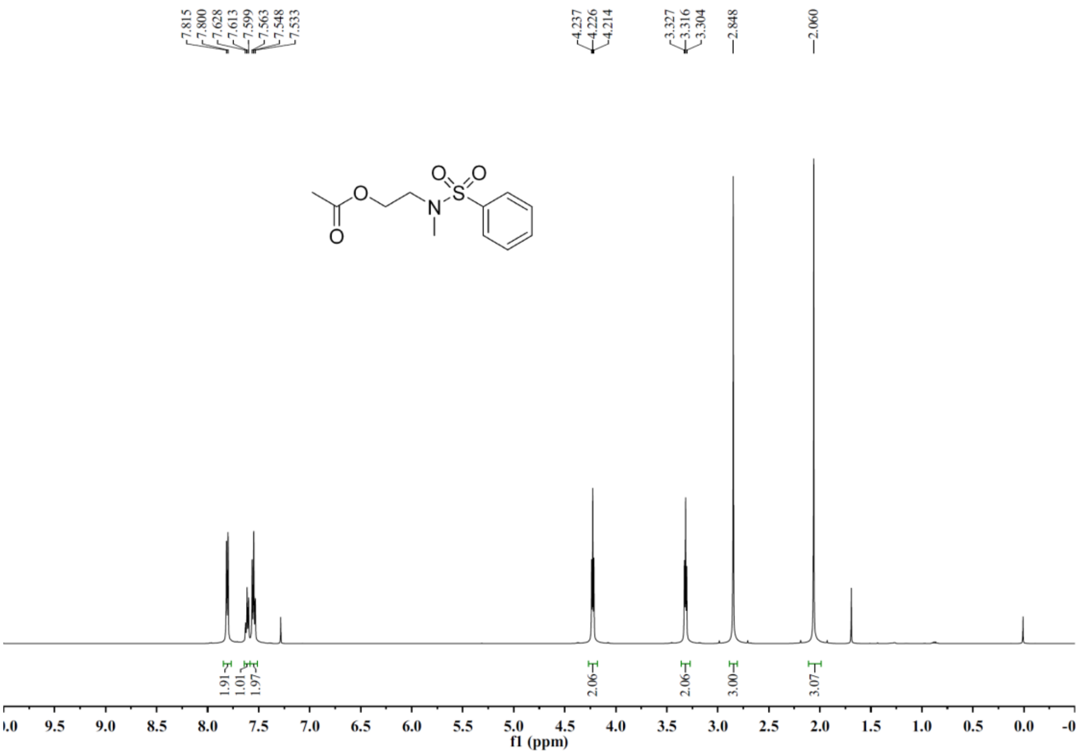

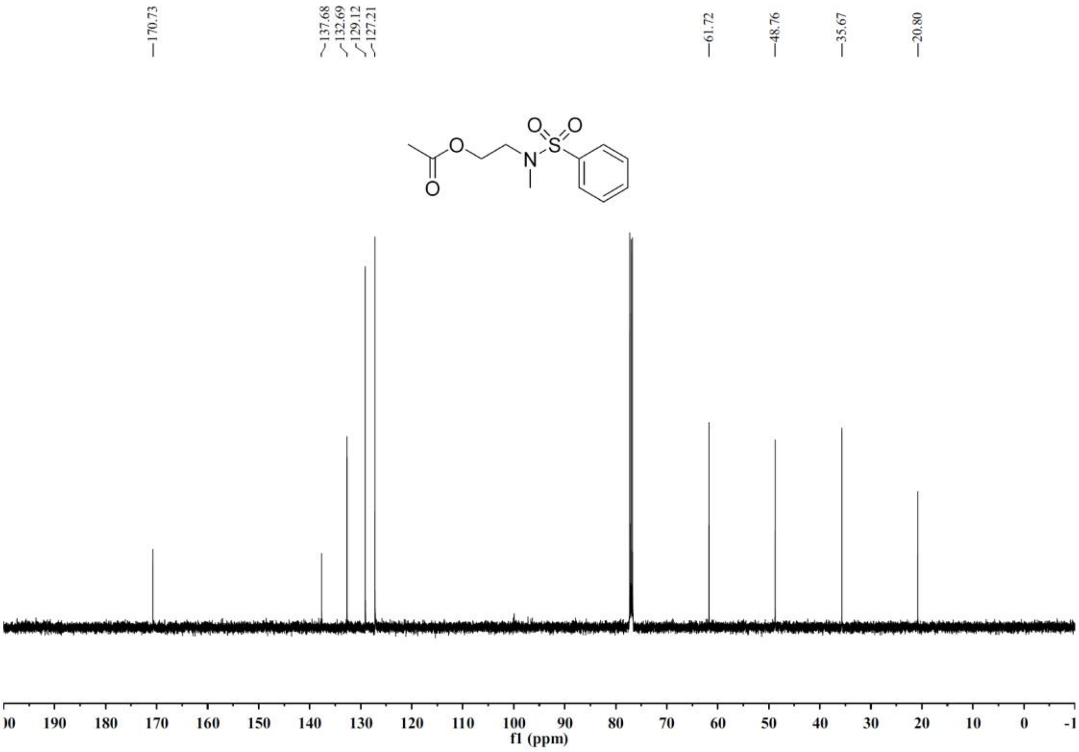

Supplementary Figure 25. NMR spectra of **3ag**

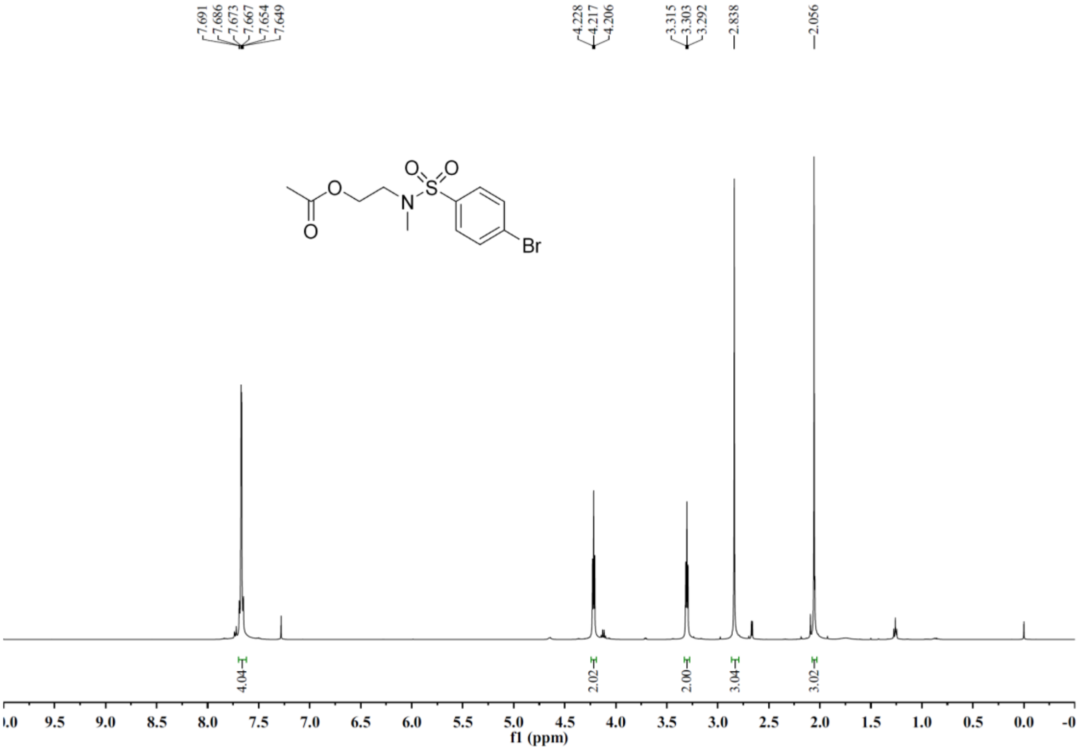

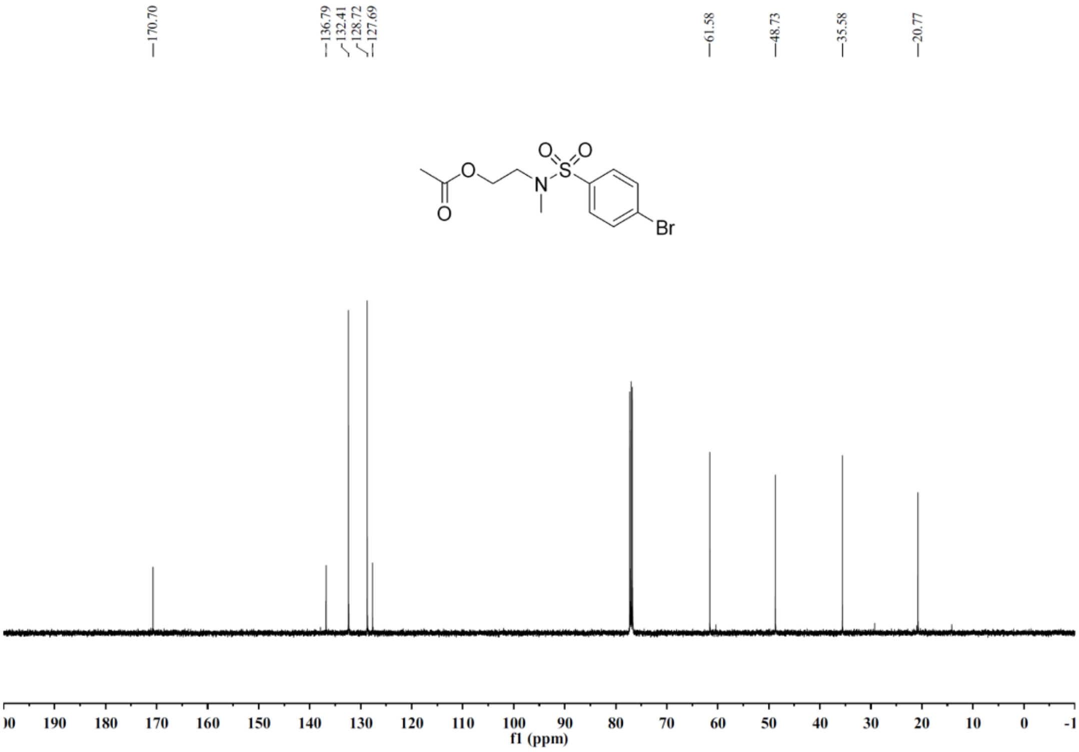

Supplementary Figure 26. NMR spectra of **3ah**

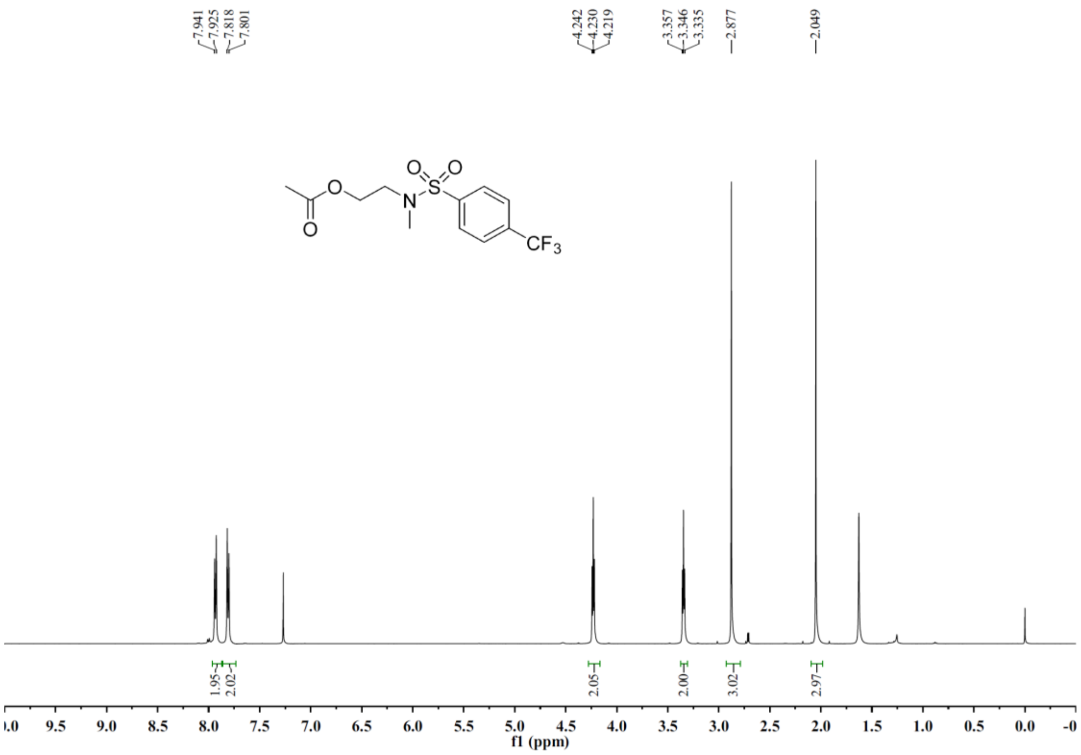

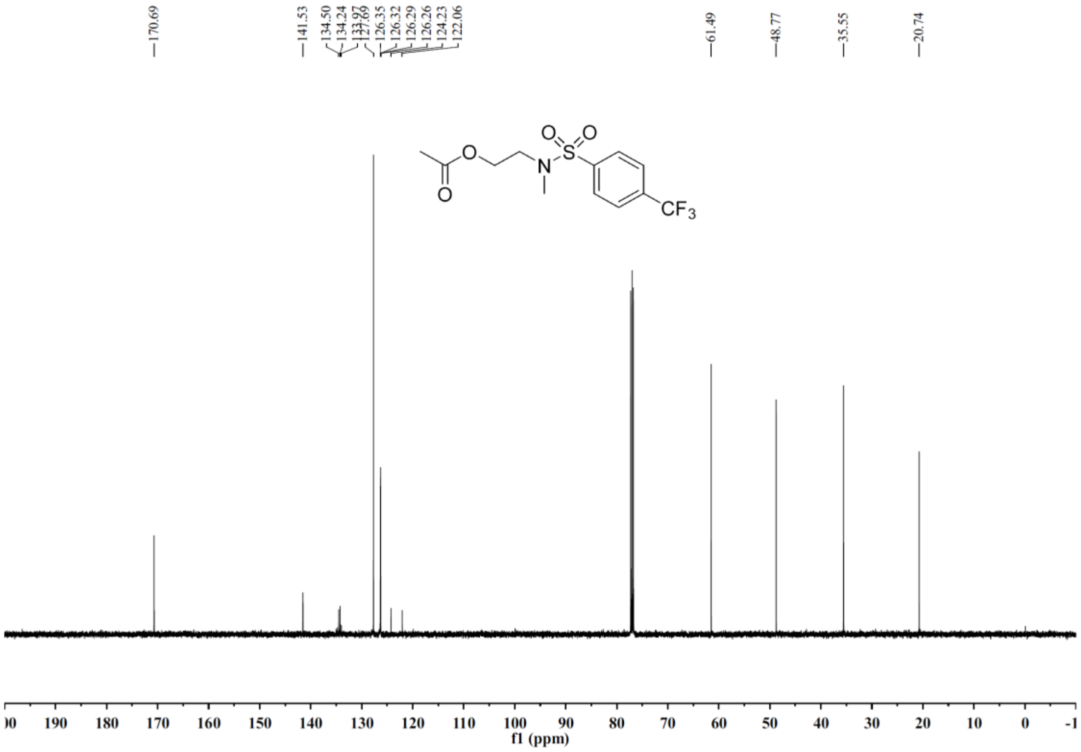

Supplementary Figure 27. NMR spectra of **3ai**

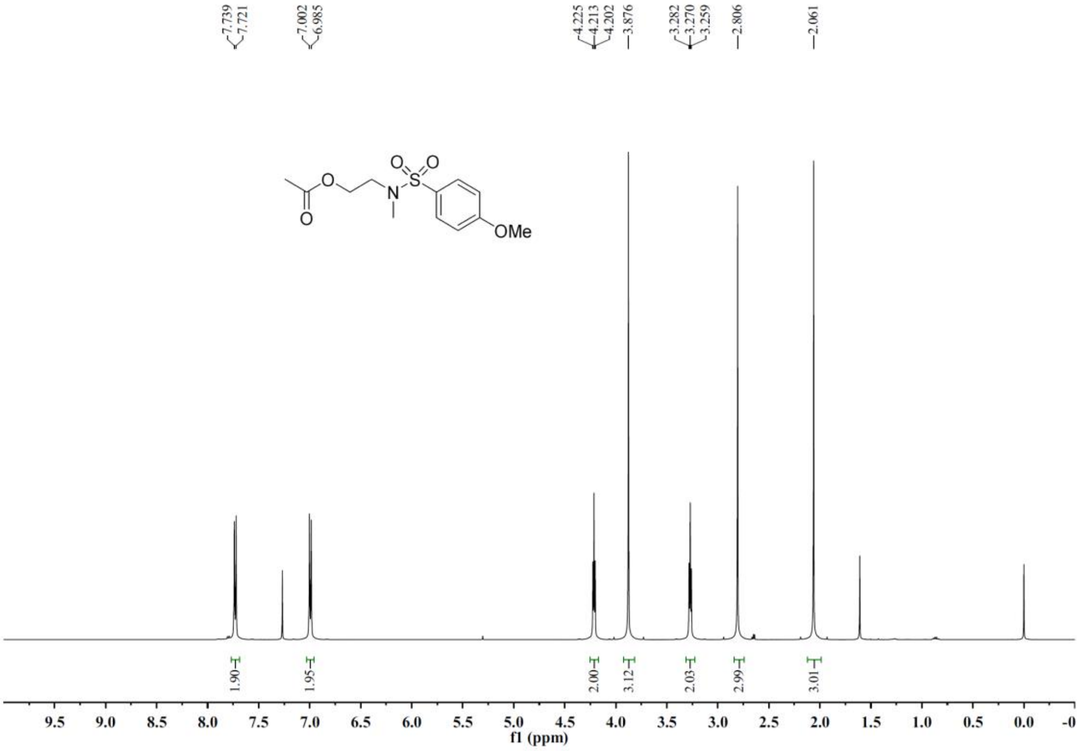

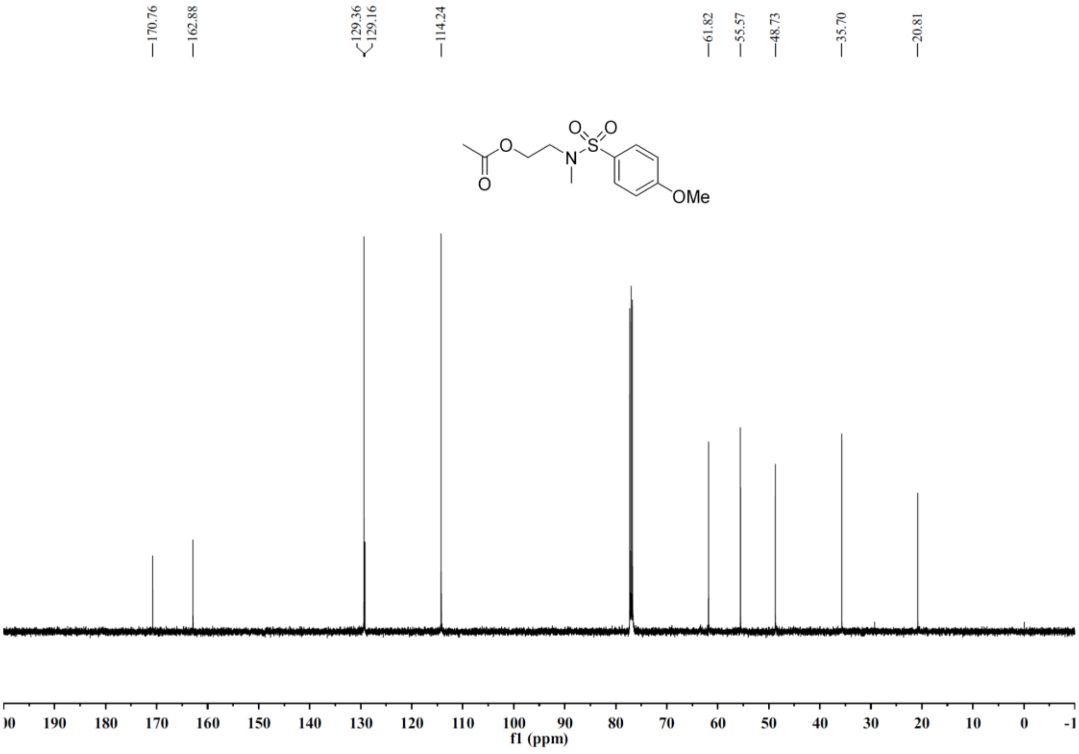

Supplementary Figure 28. NMR spectra of **3aj**

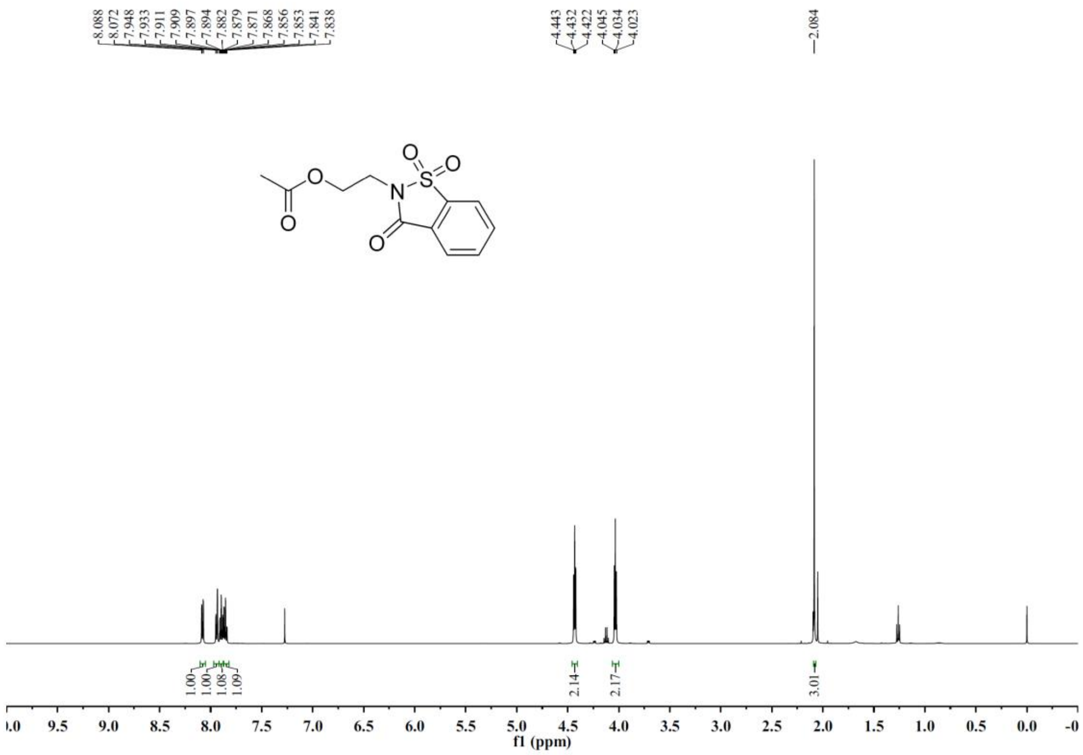

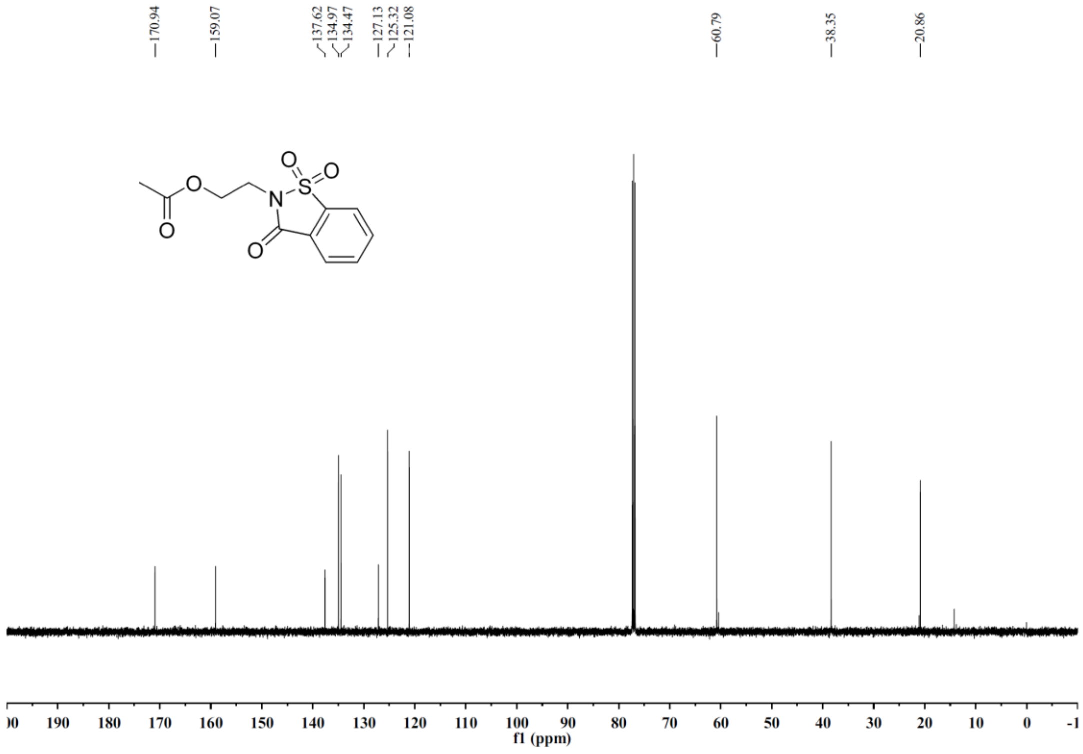

Supplementary Figure 29. NMR spectra of **3am**

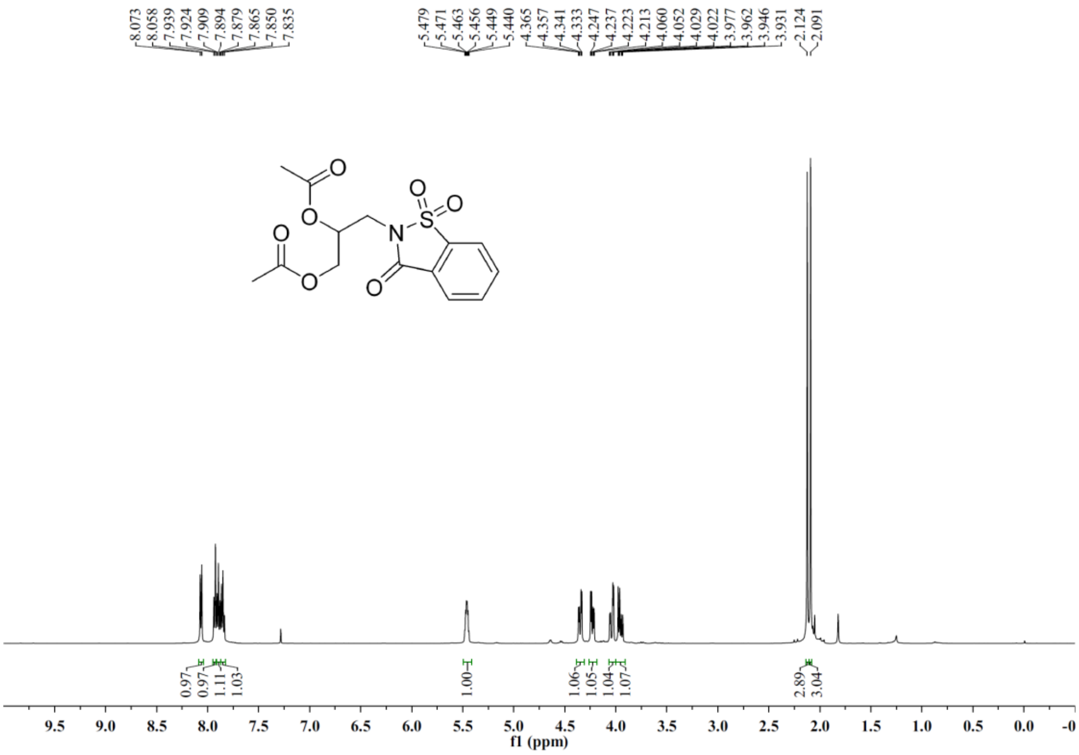

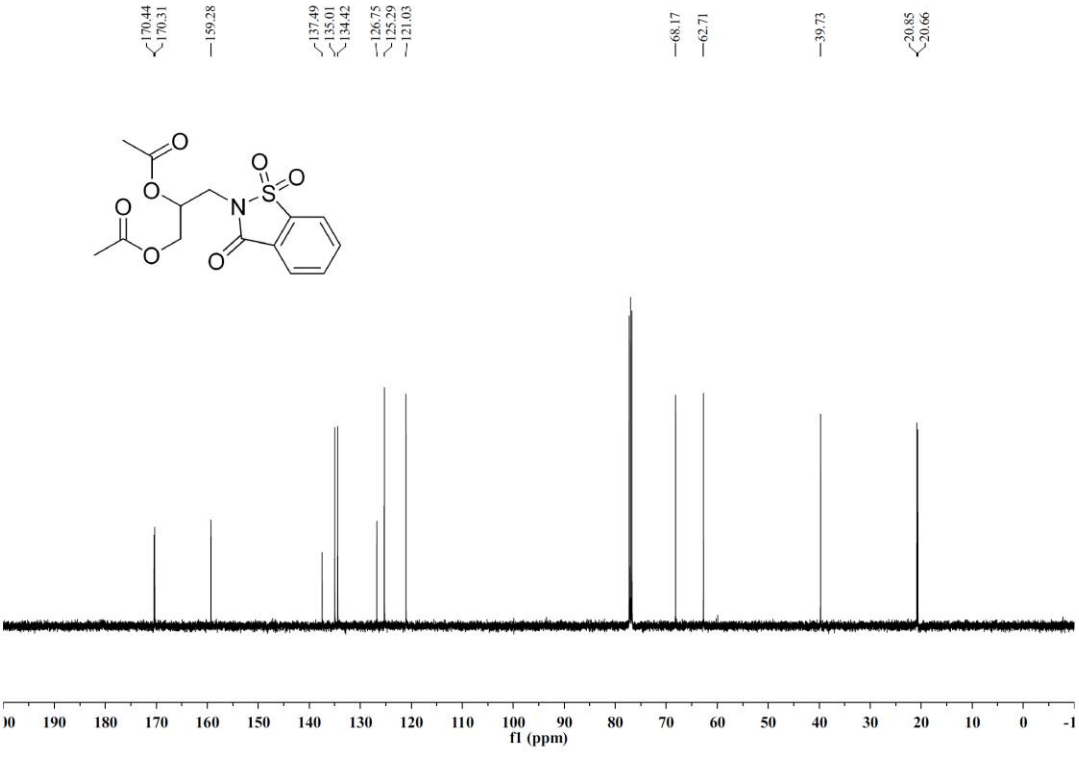

Supplementary Figure 30. NMR spectra of **3em**

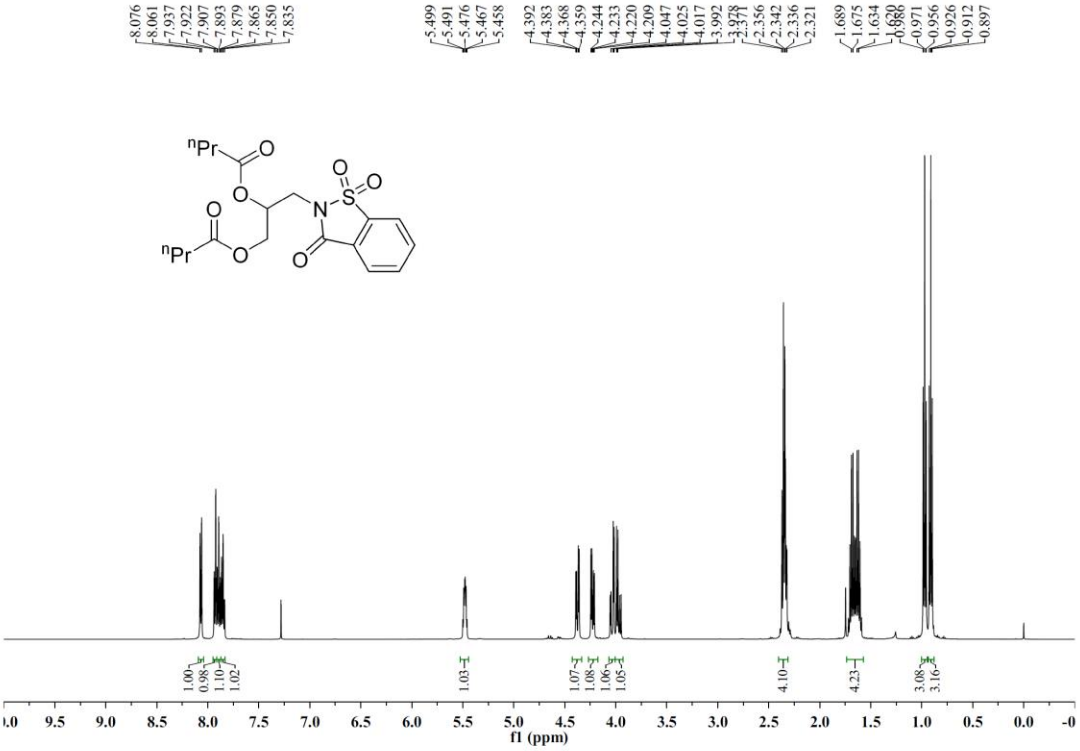

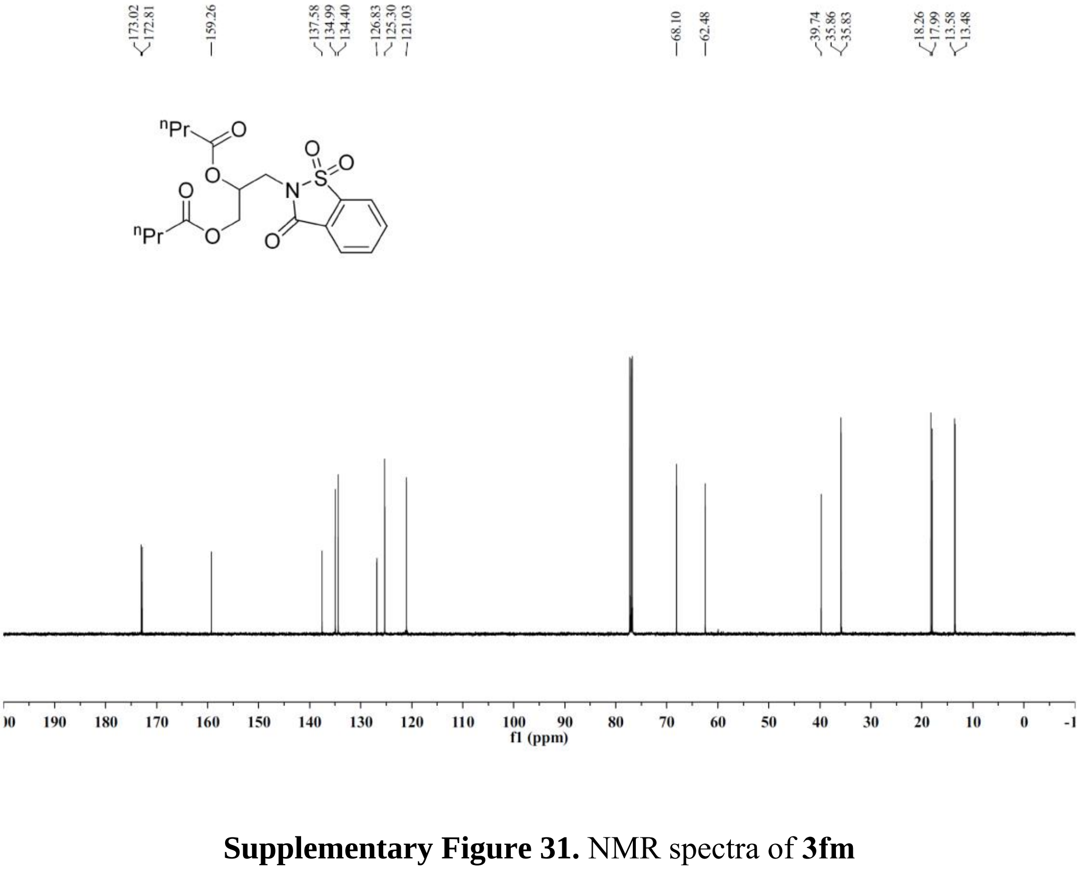

Supplementary Figure 31. NMR spectra of **3fm**

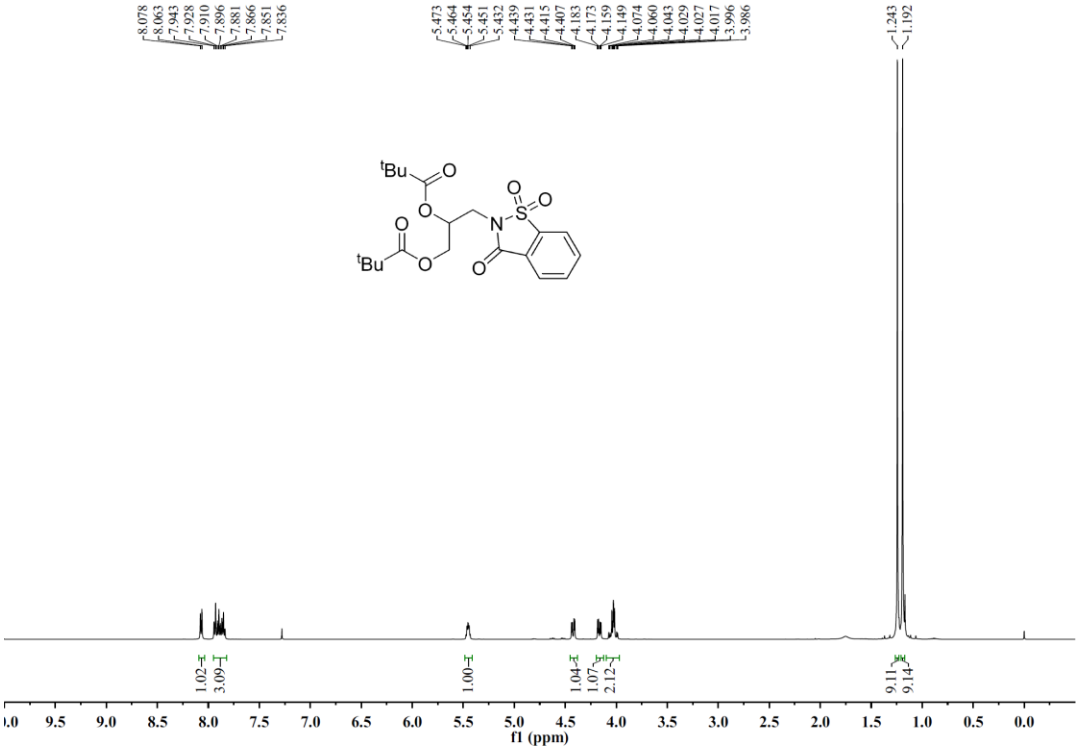

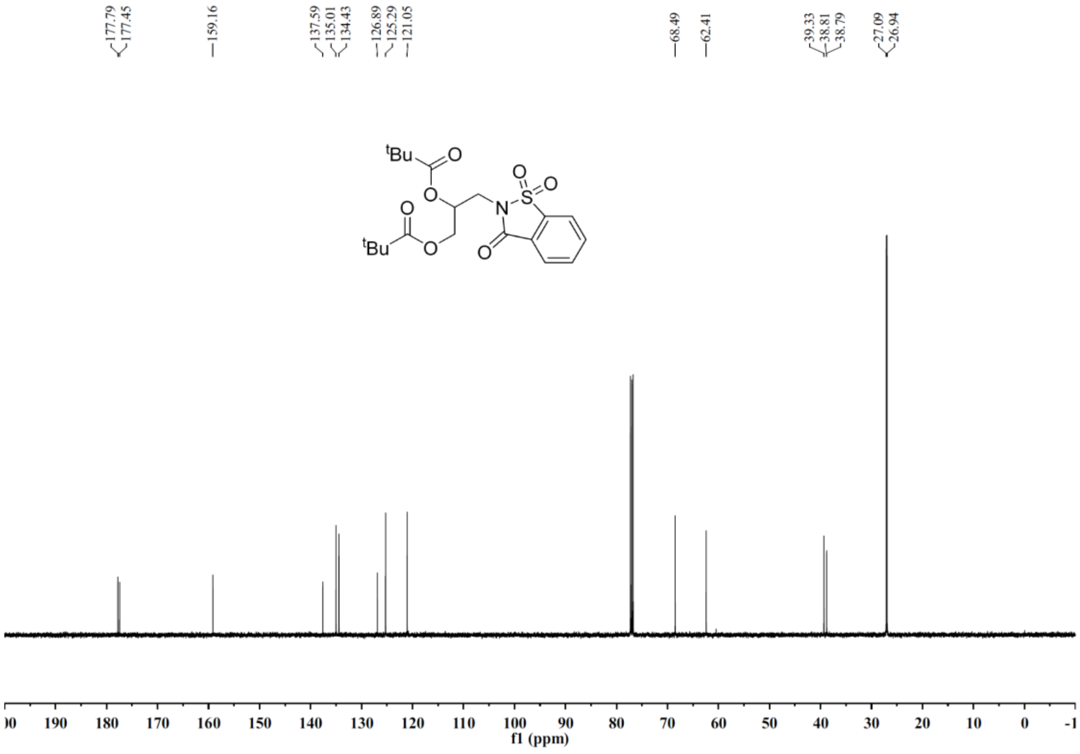

Supplementary Figure 32. NMR spectra of **3gm**

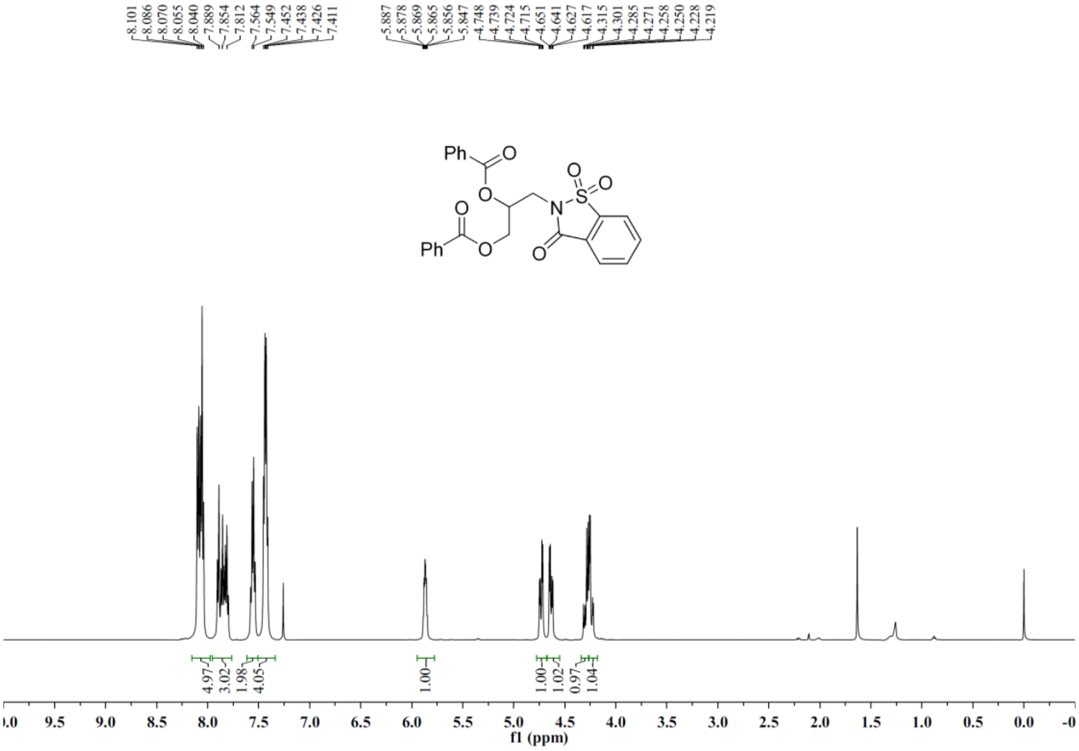

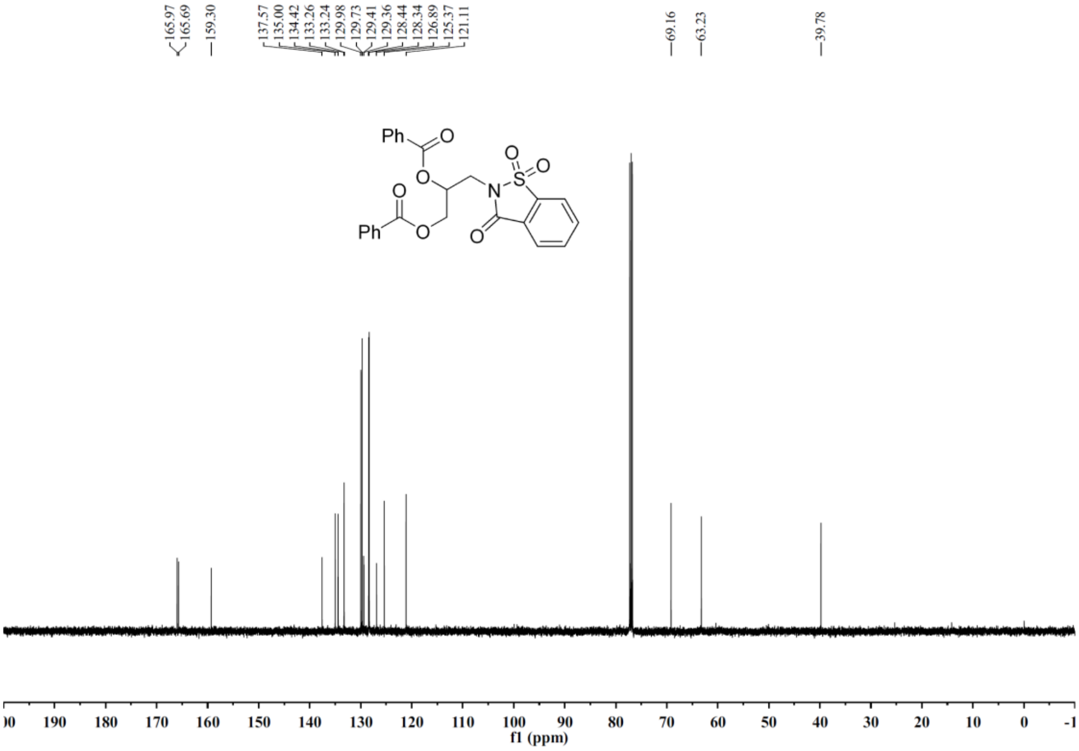

Supplementary Figure 33. NMR spectra of **3hm**

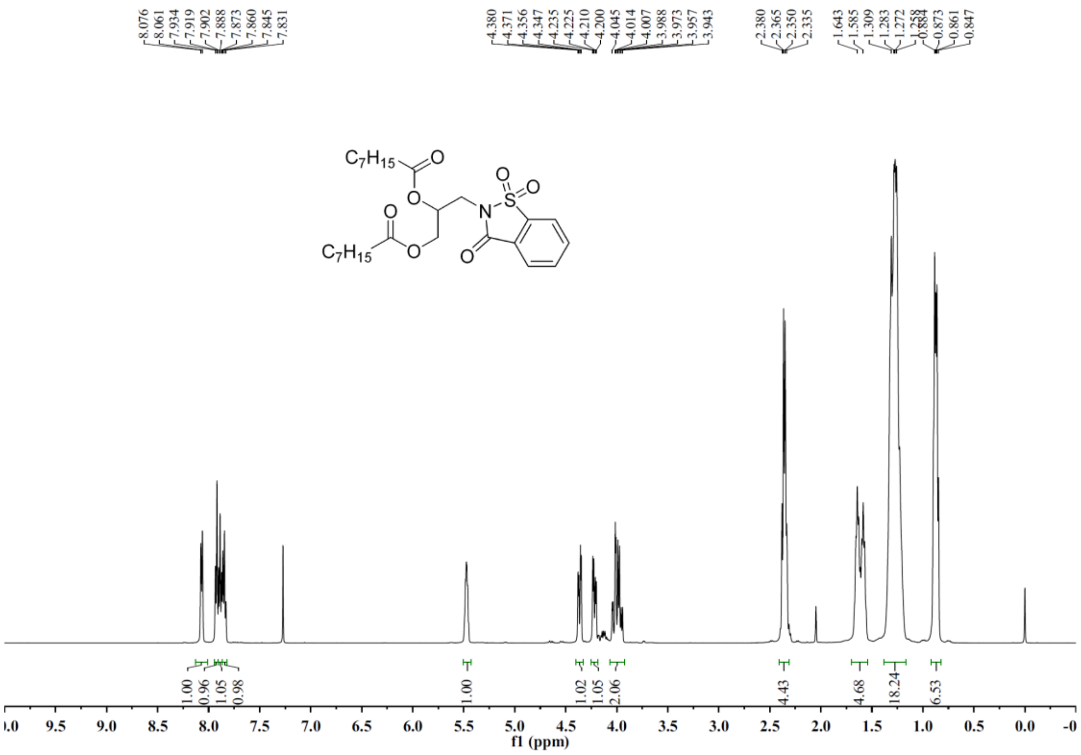

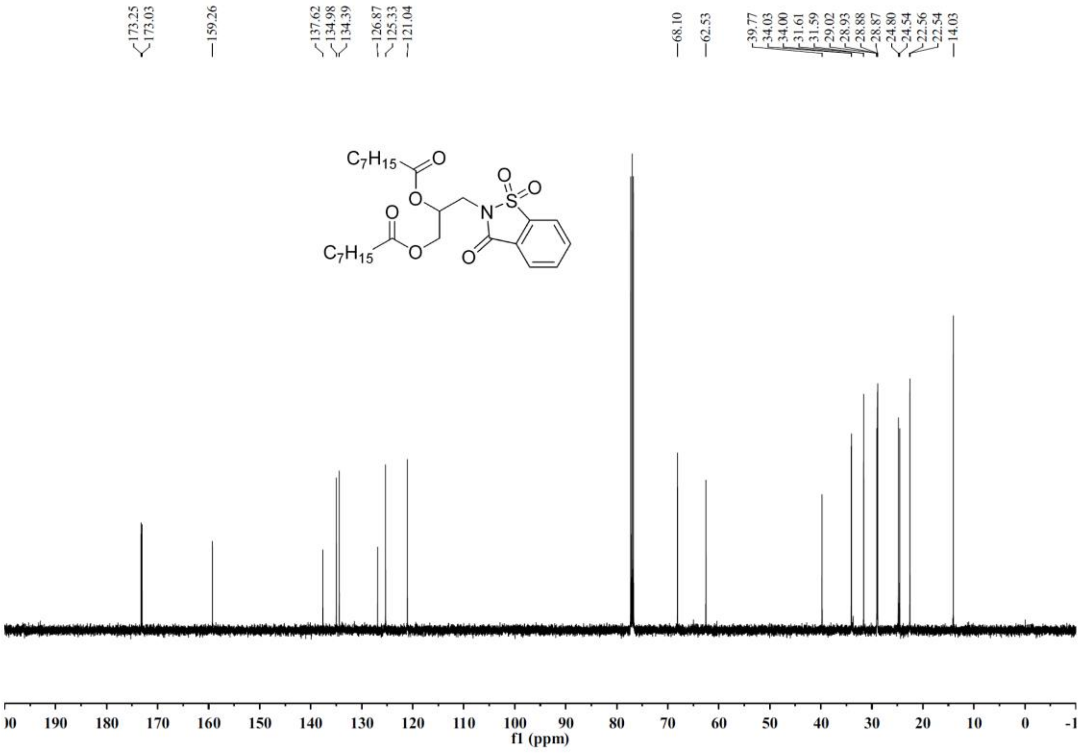

Supplementary Figure 34. NMR spectra of **3im**

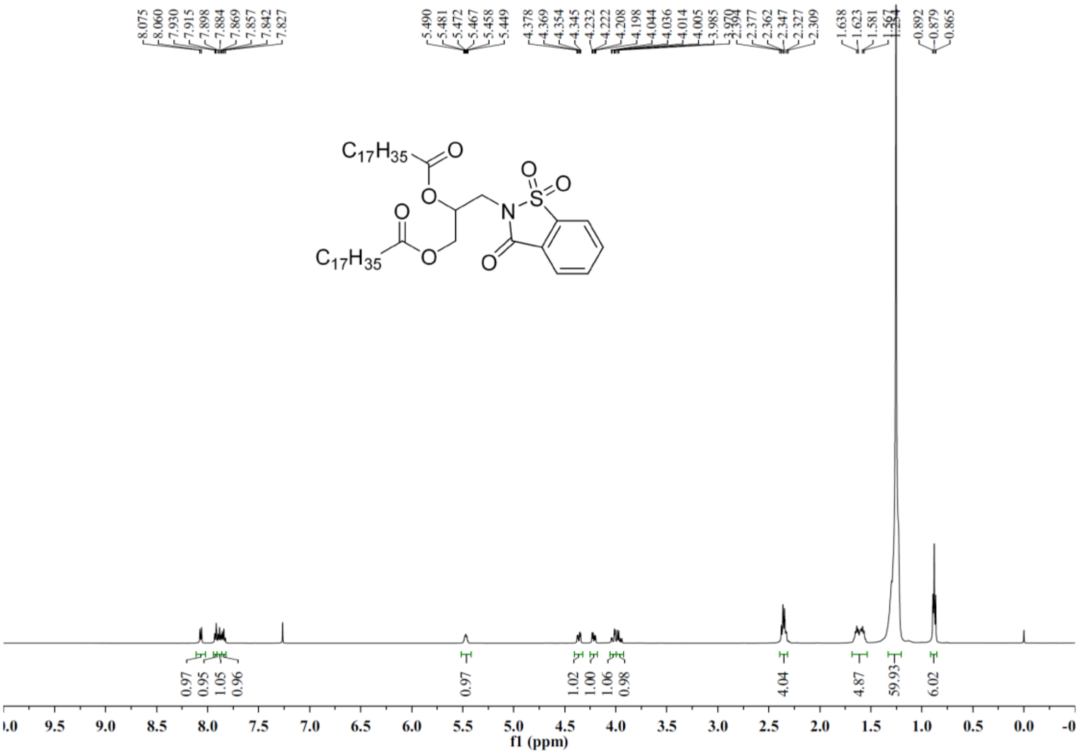

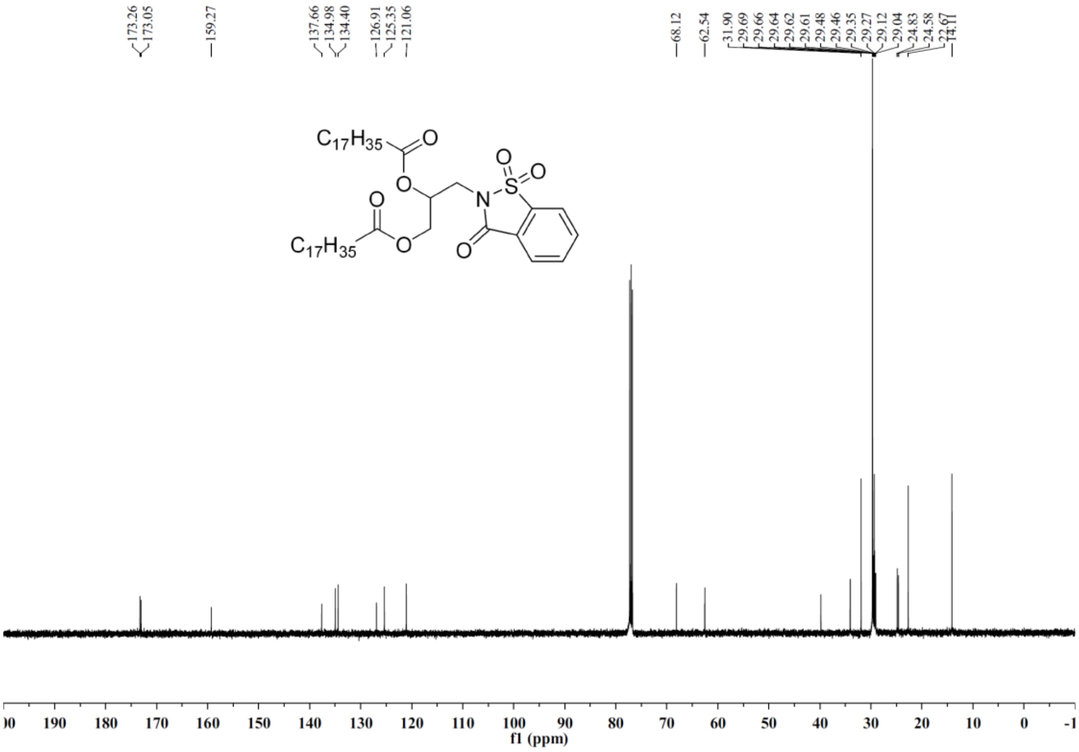

Supplementary Figure 35. NMR spectra of **3jm**

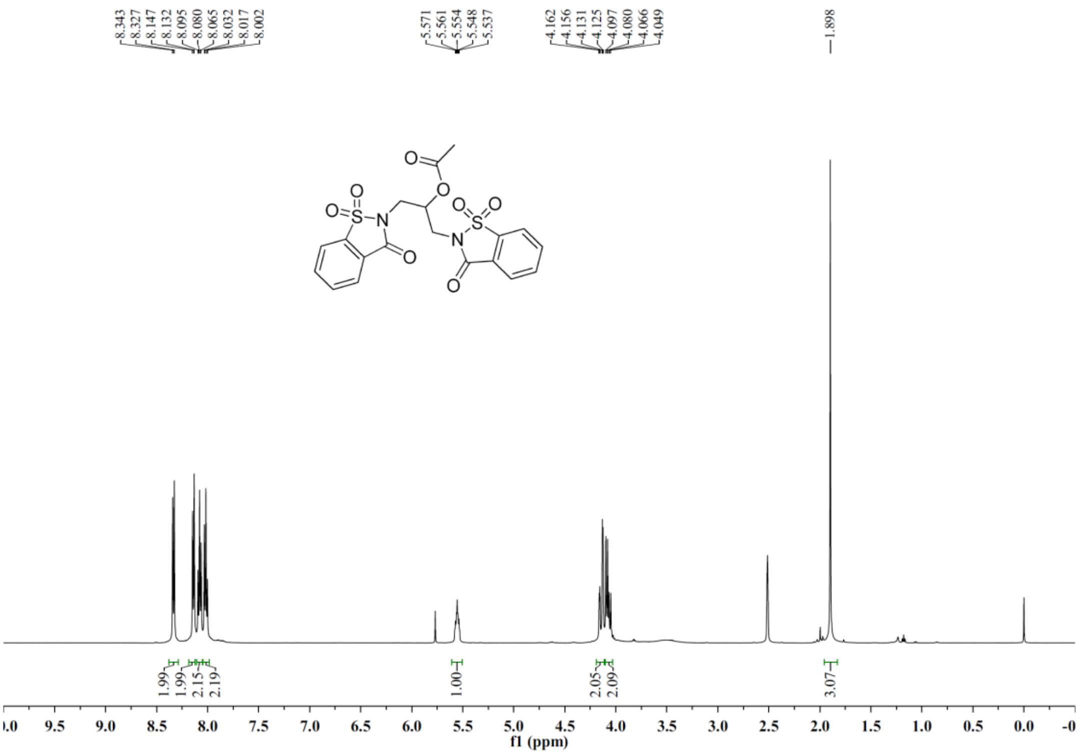

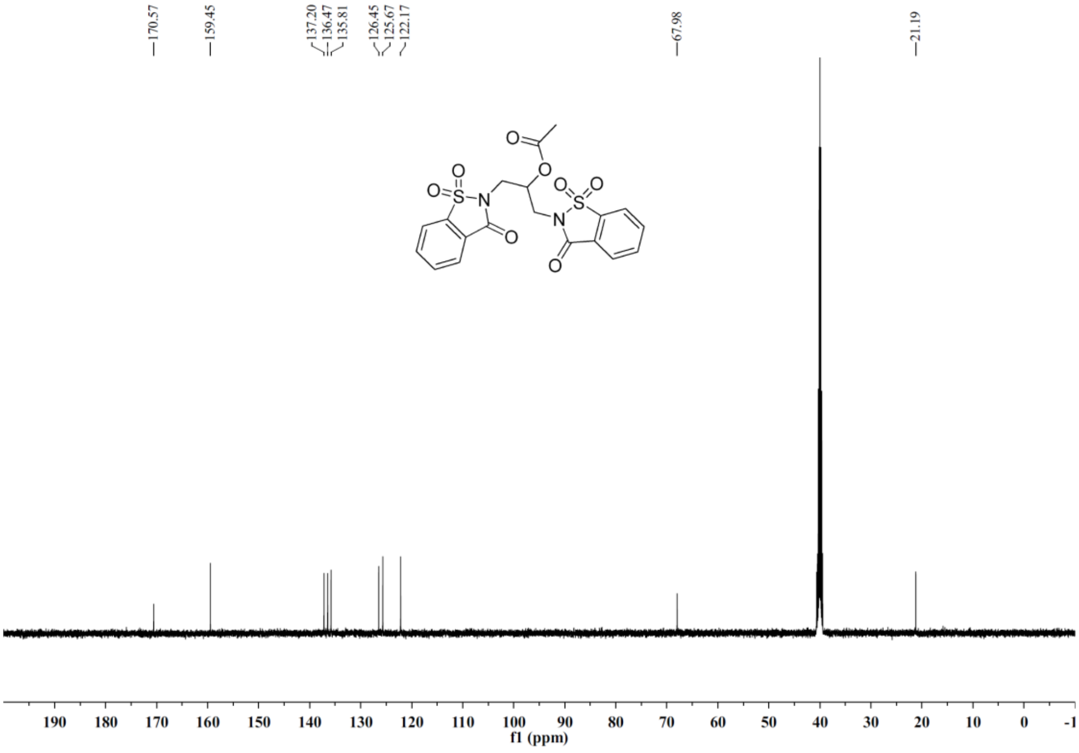

Supplementary Figure 36. NMR spectra of **3emm**

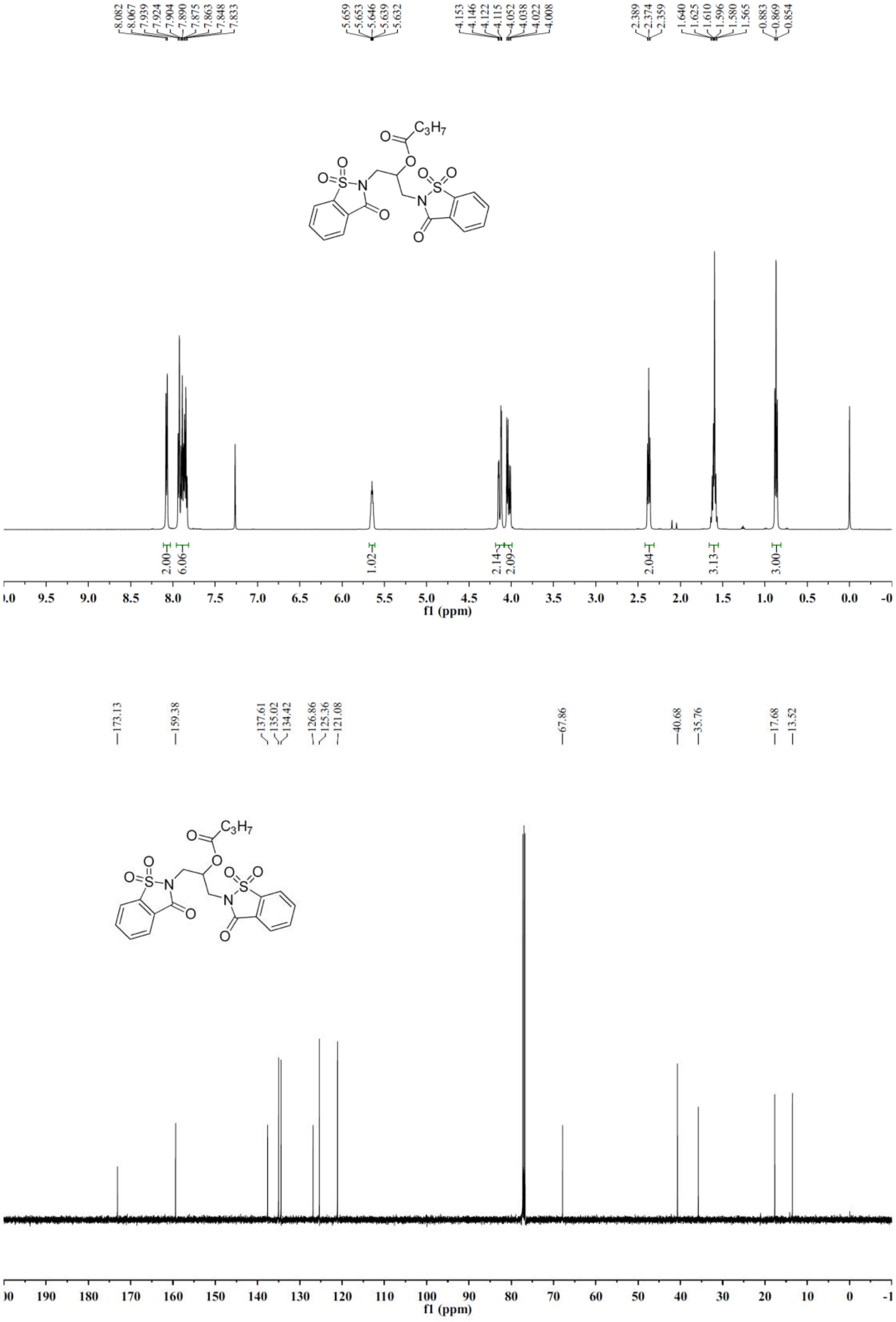

Supplementary Figure 37. NMR spectra of **3fmm**

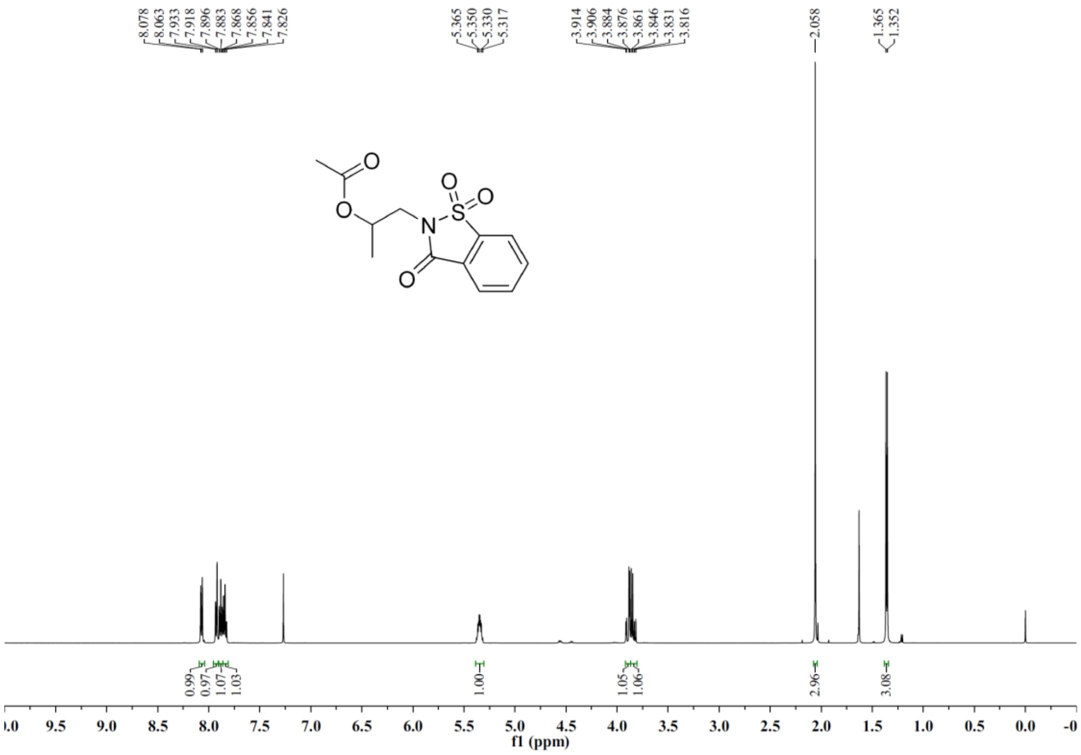

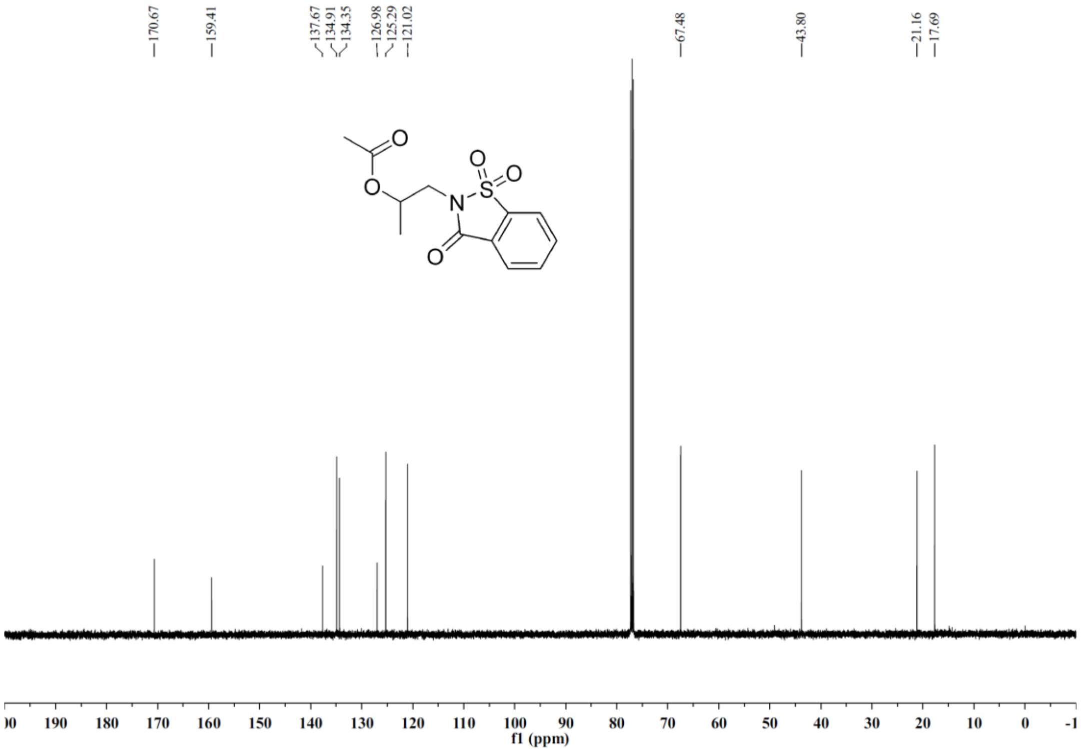

Supplementary Figure 38. NMR spectra of **3km**

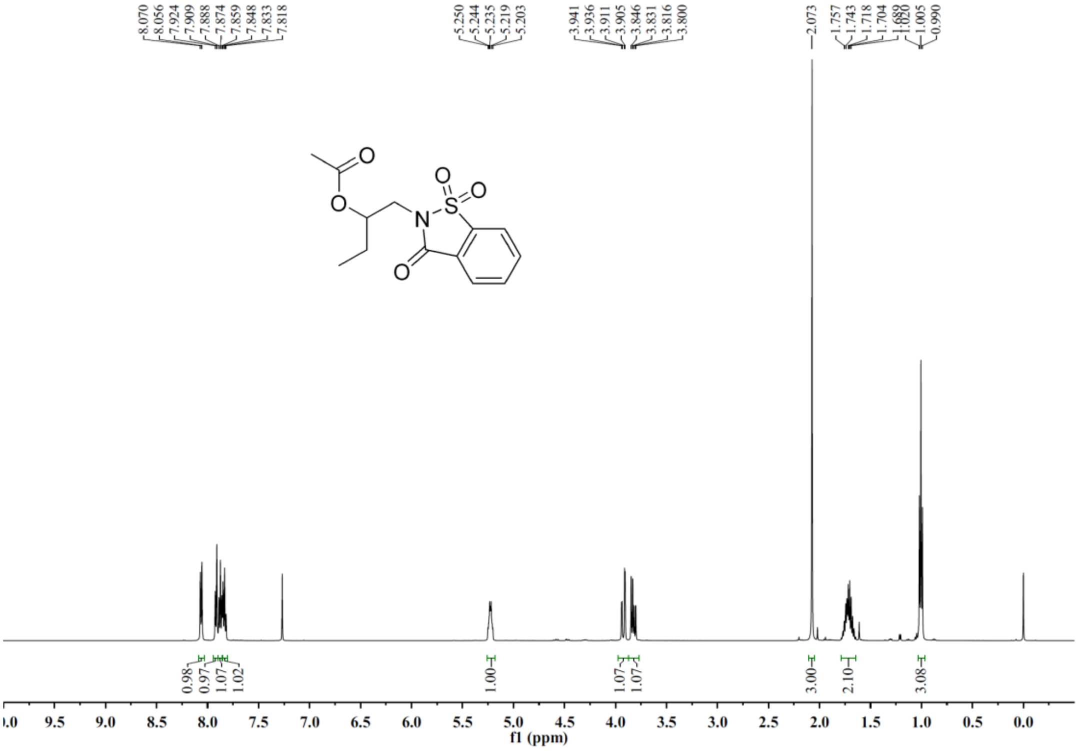

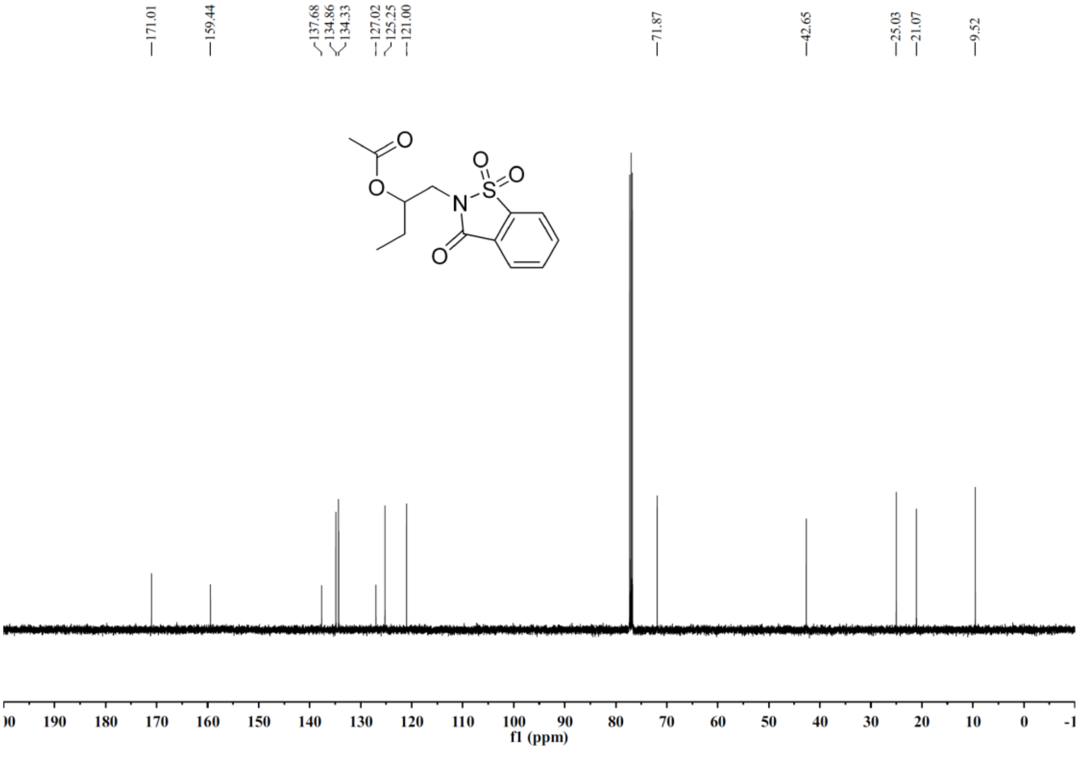

Supplementary Figure 39. NMR spectra of **3lm**

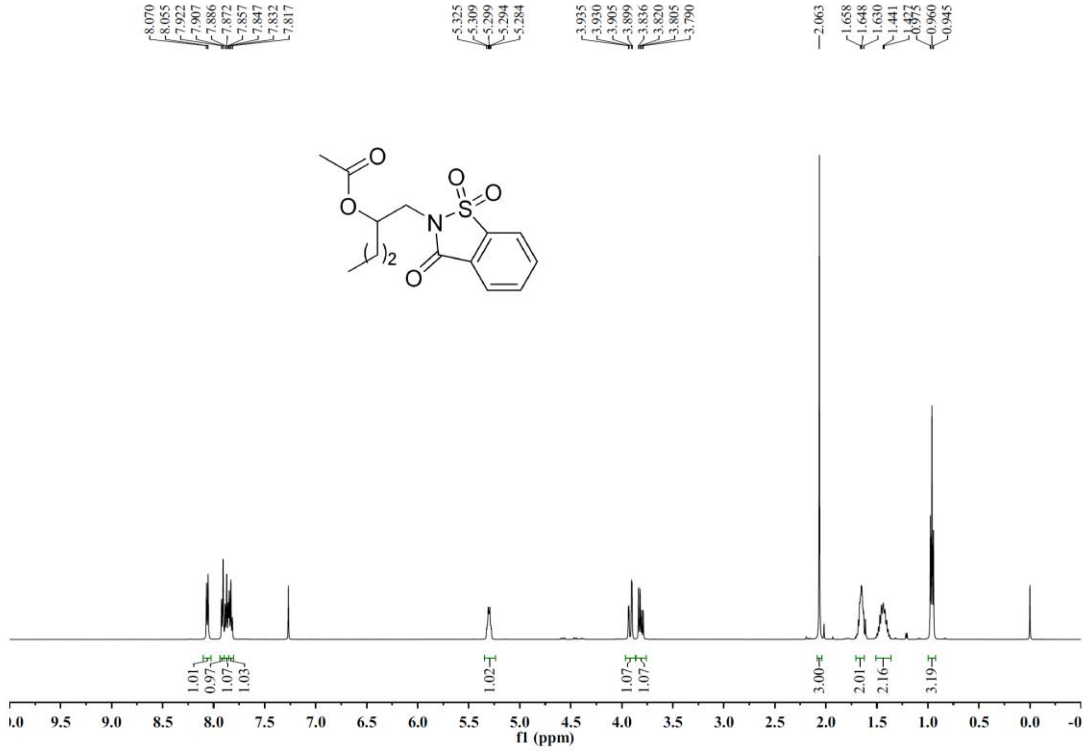

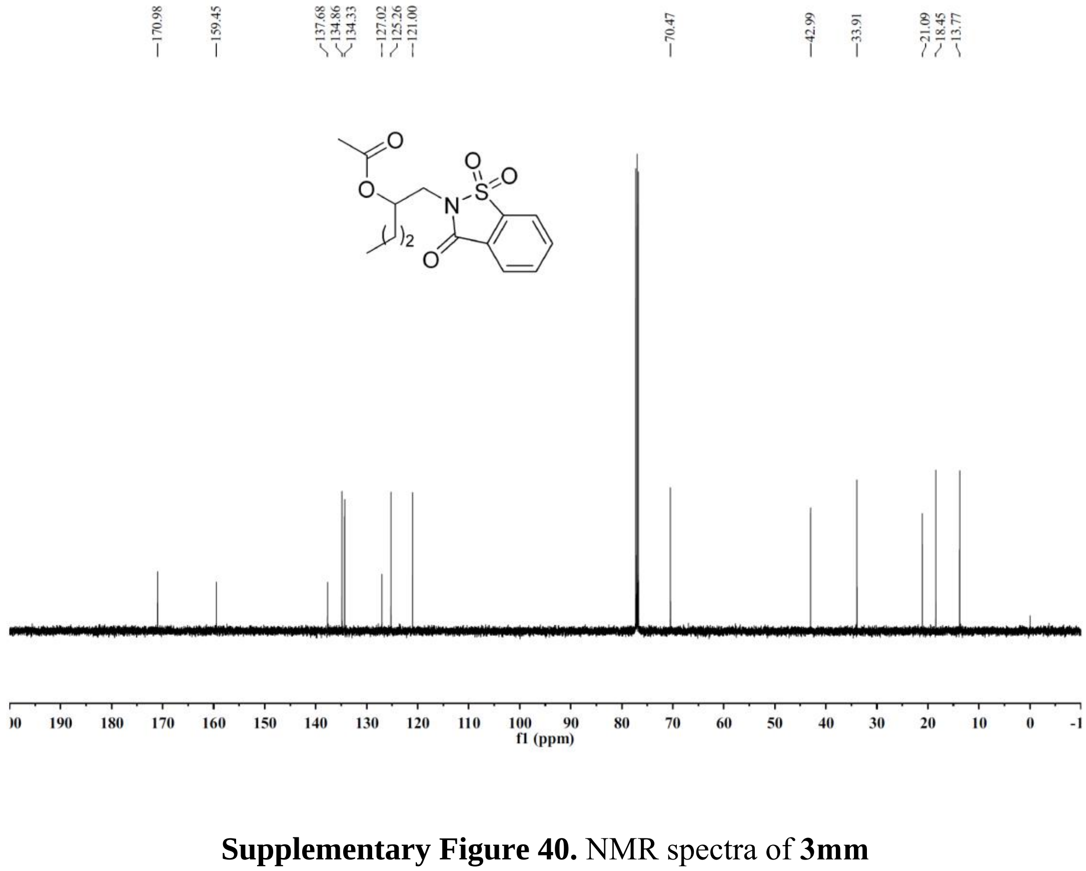

Supplementary Figure 40. NMR spectra of **3mm**

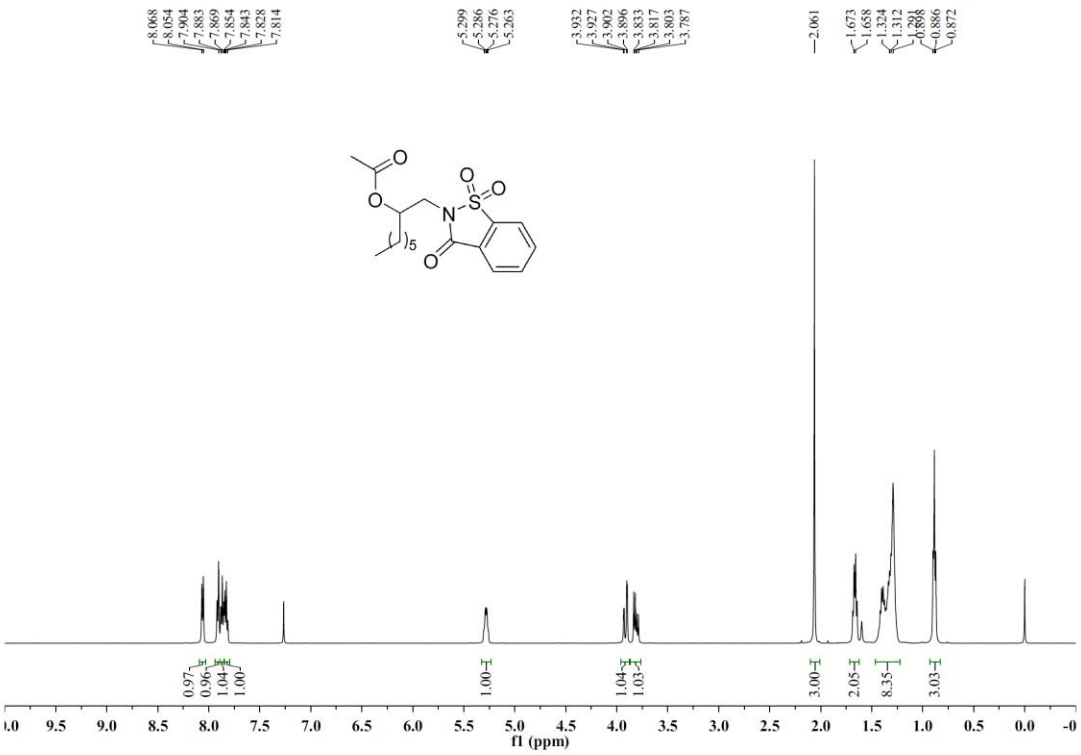

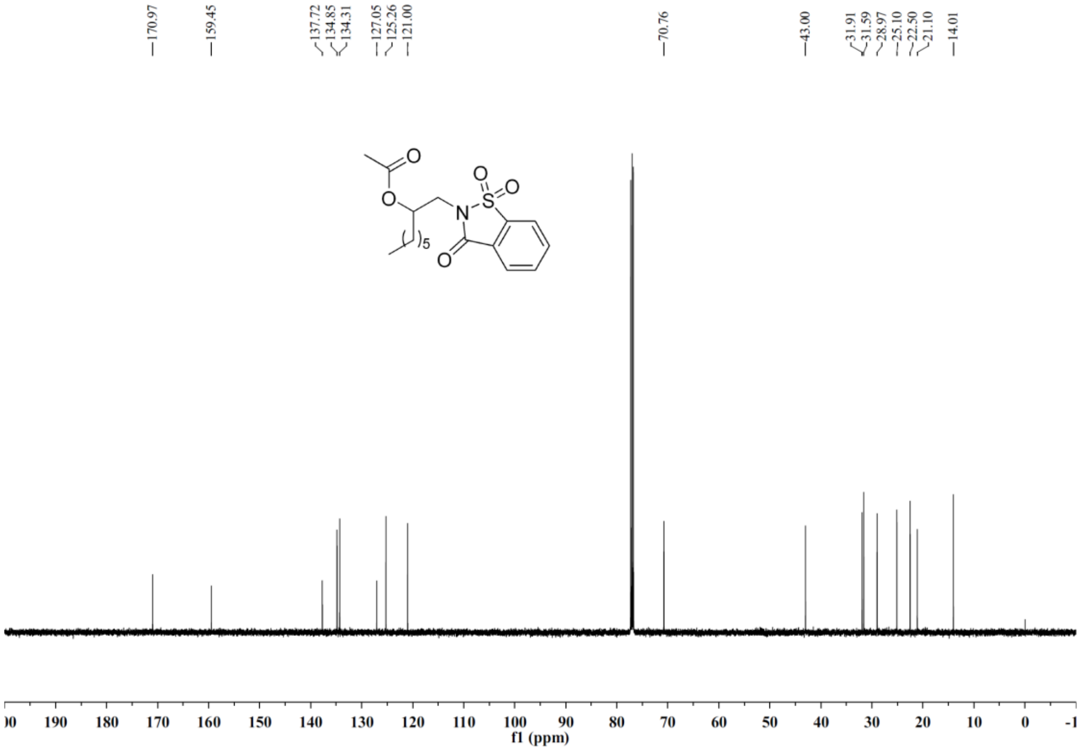

Supplementary Figure 41. NMR spectra of **3nm**

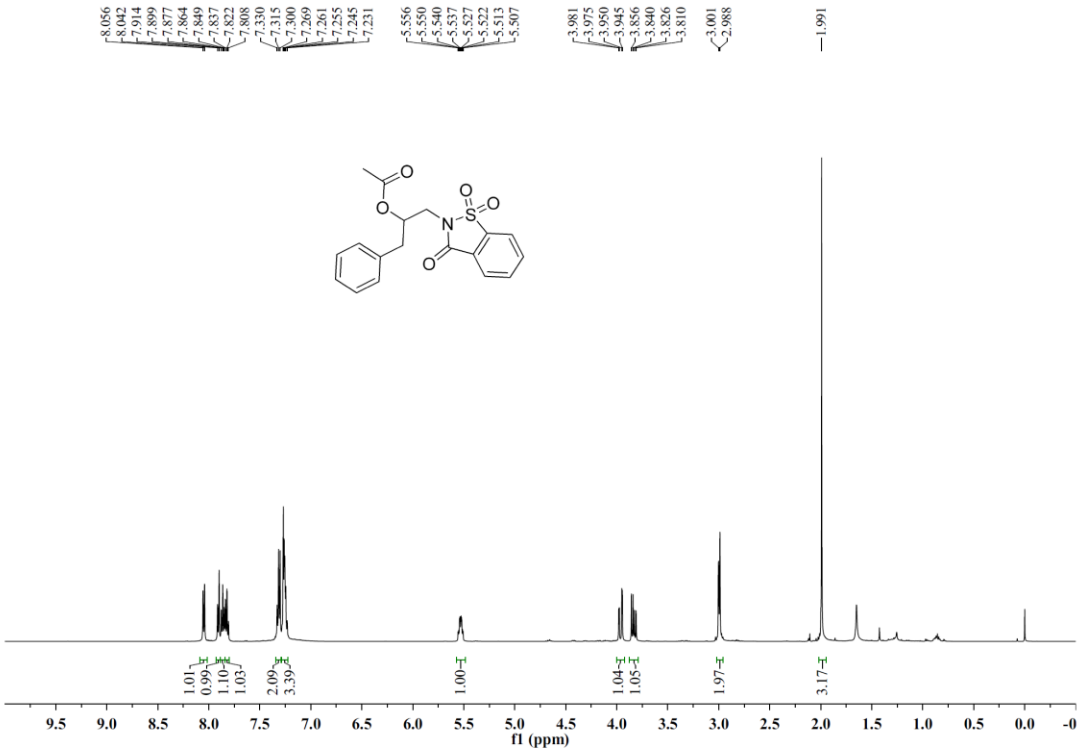

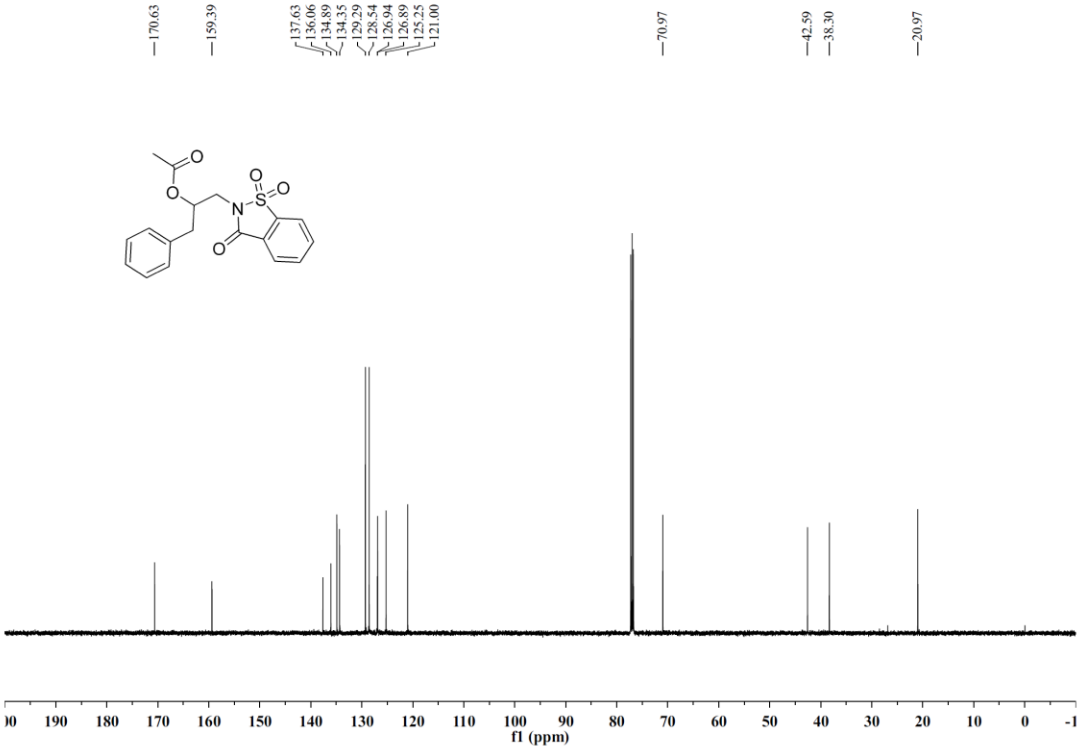

Supplementary Figure 42. NMR spectra of **3om**

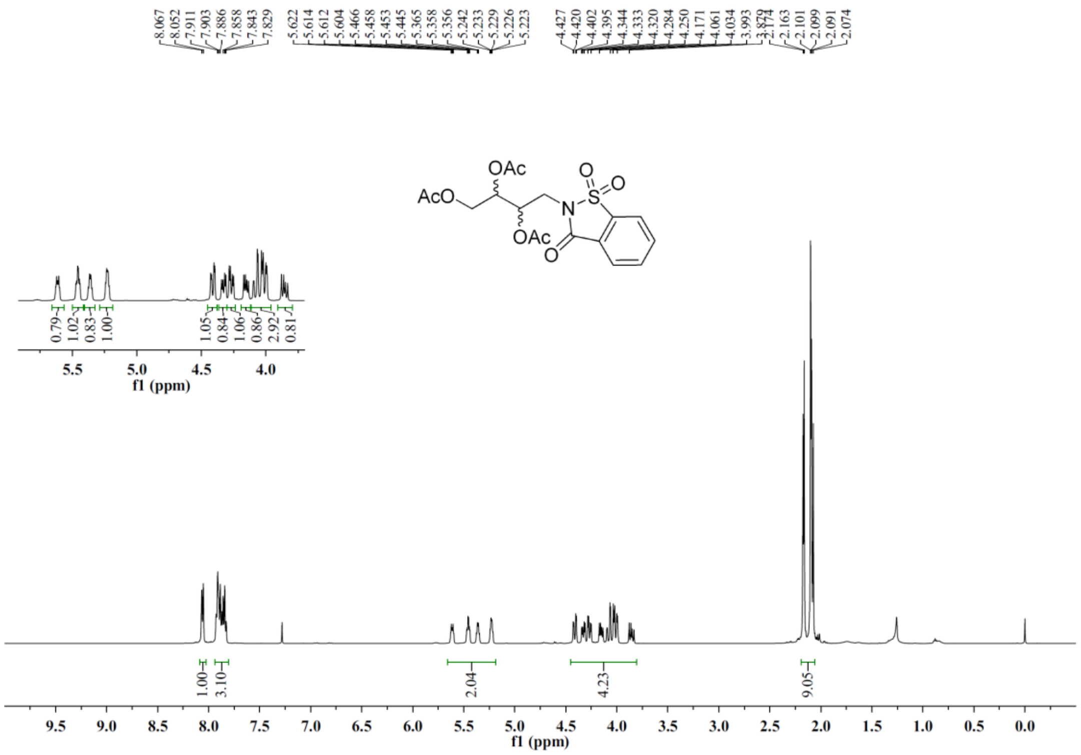

Supplementary Figure 43. NMR spectra of **3pm** (full)

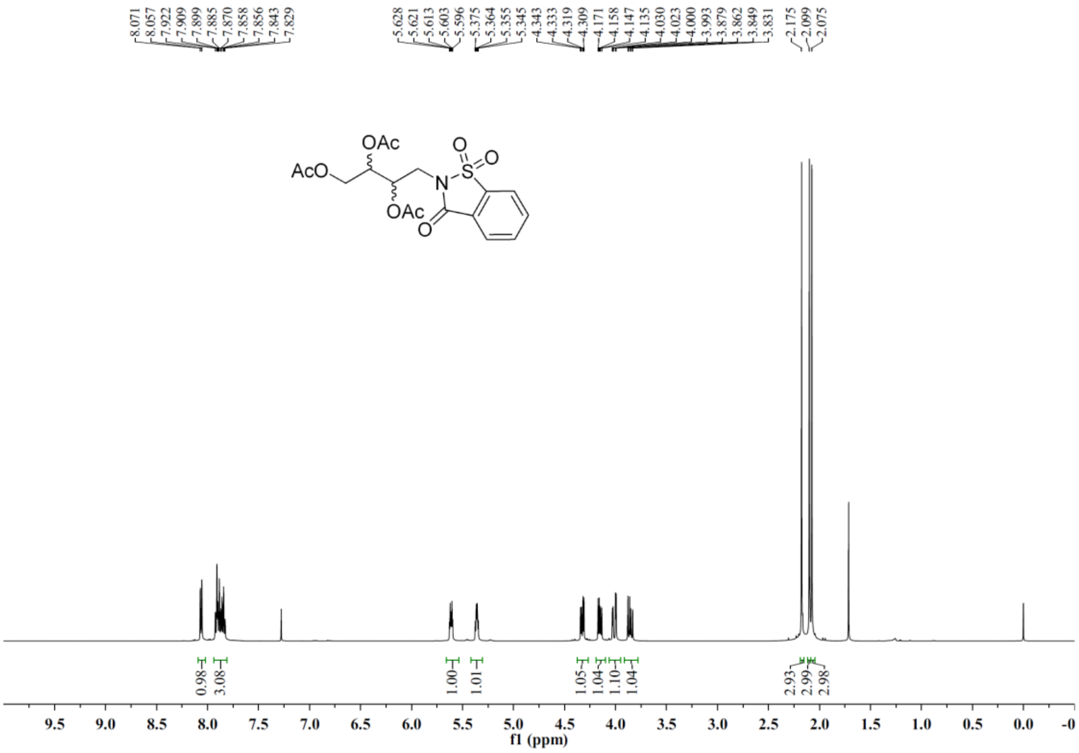

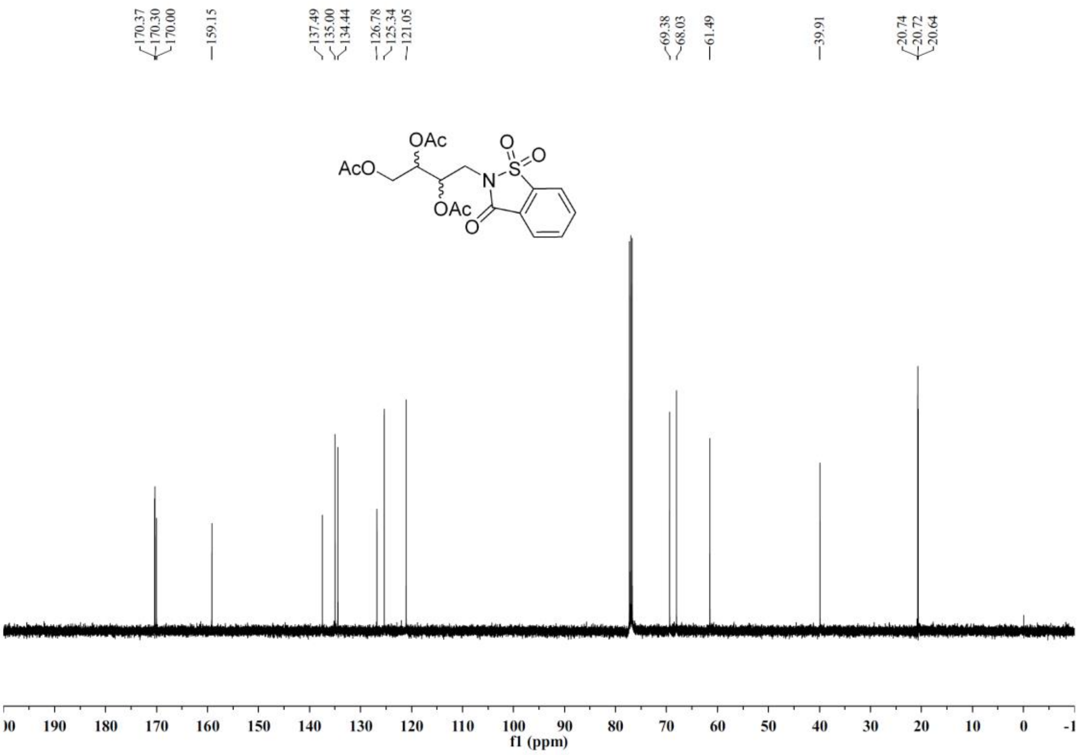

Supplementary Figure 44. NMR spectra of **3pm** (recrystallized from isopropanol)

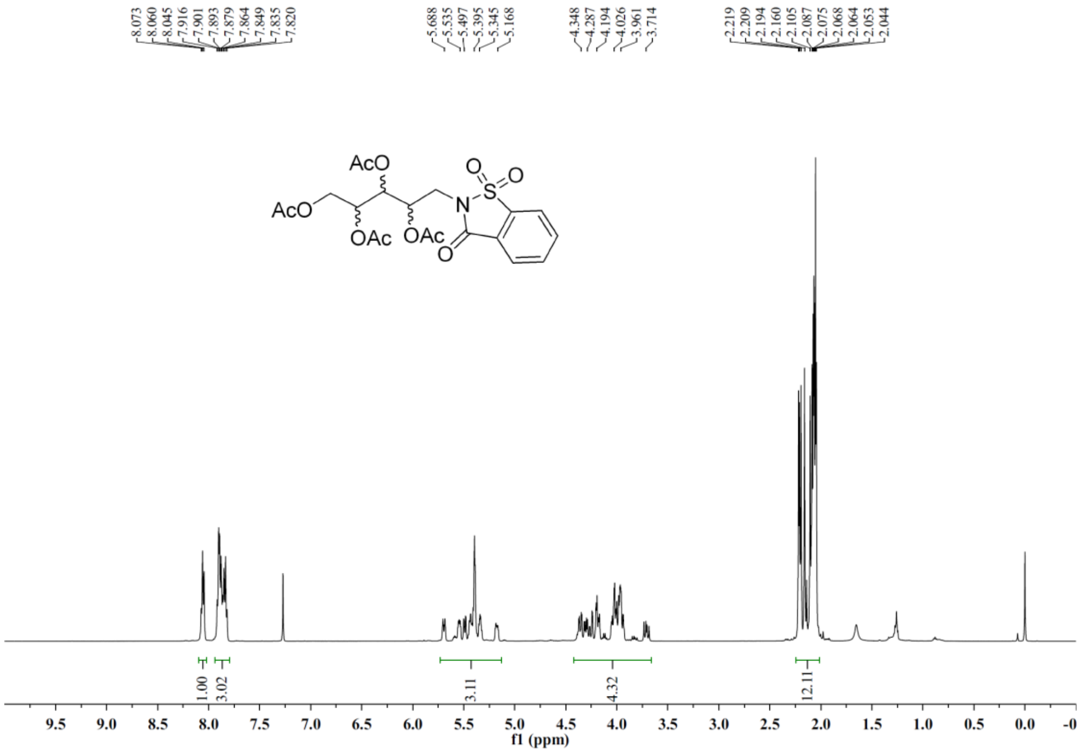

Supplementary Figure 45. NMR spectrum of **3qm** (full)

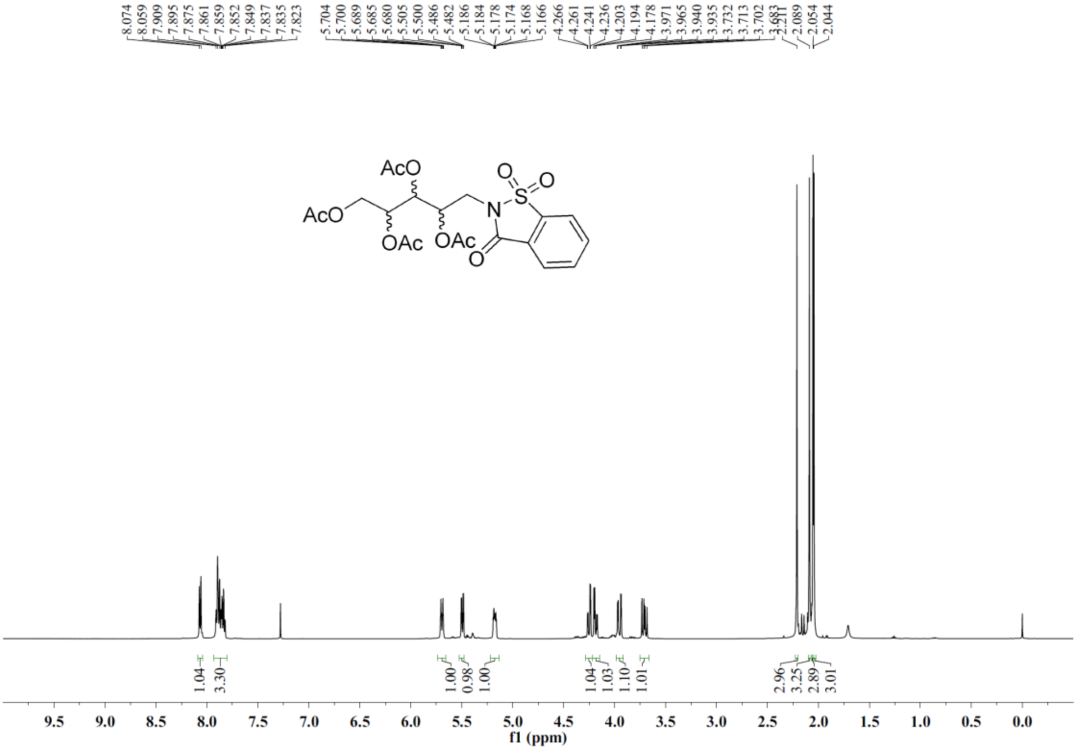

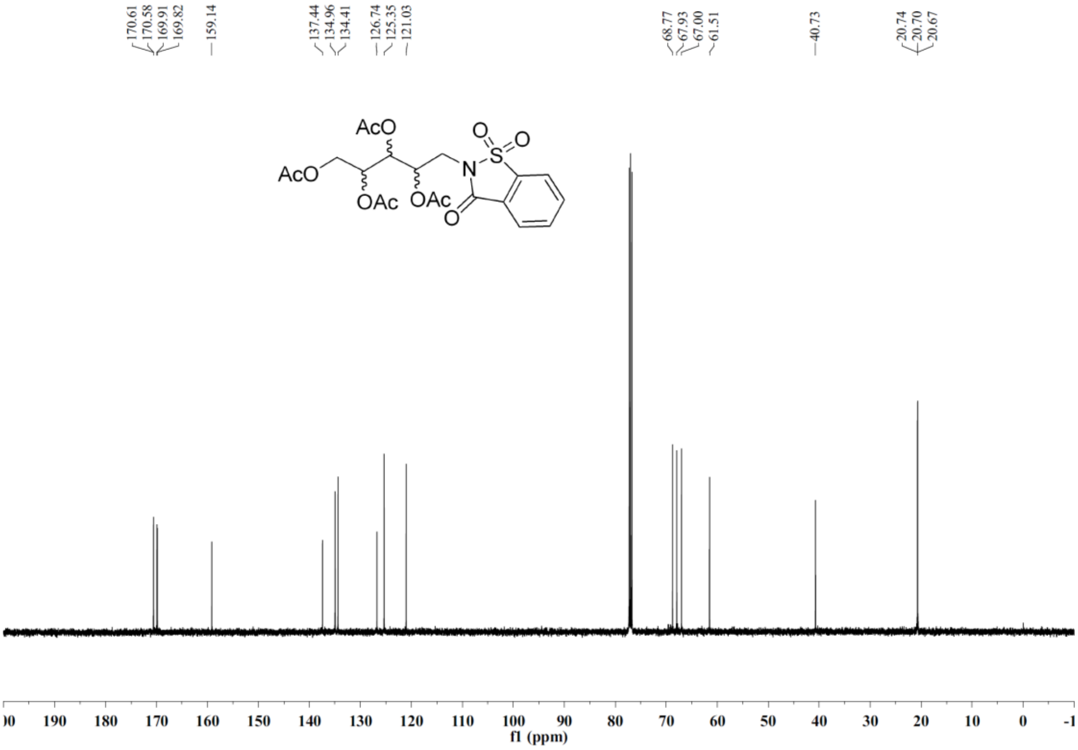

Supplementary Figure 46. NMR spectra of **3qm** (recrystallized from isopropanol)

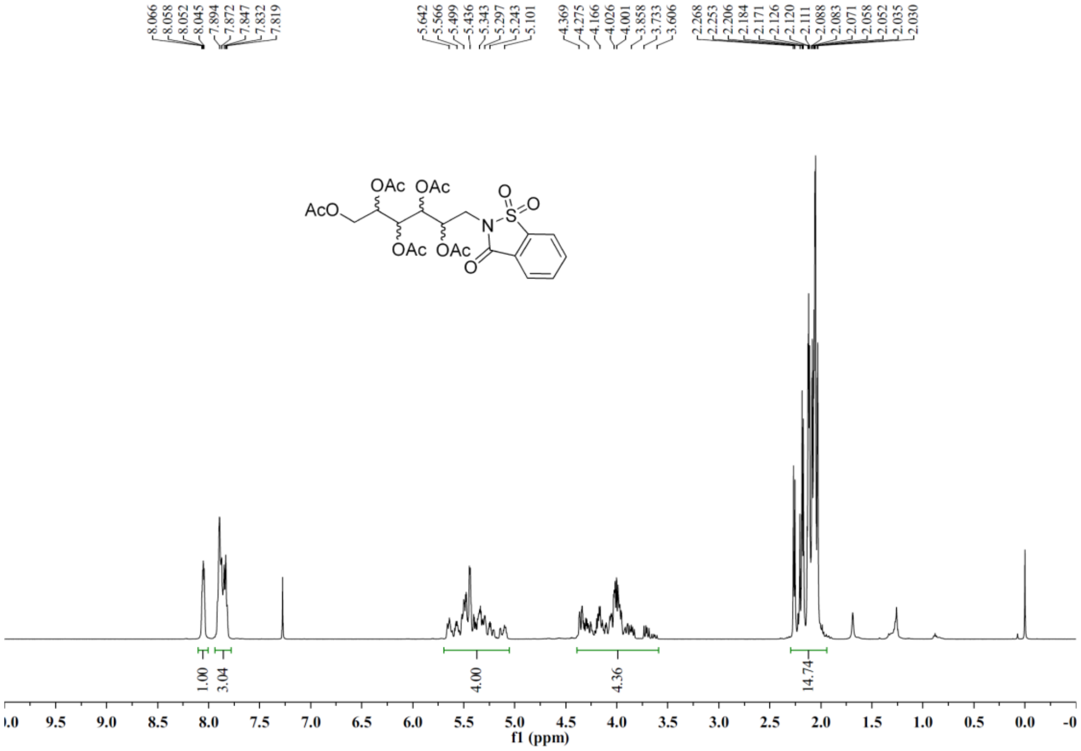

Supplementary Figure 47. NMR spectra of **3rm** (full)

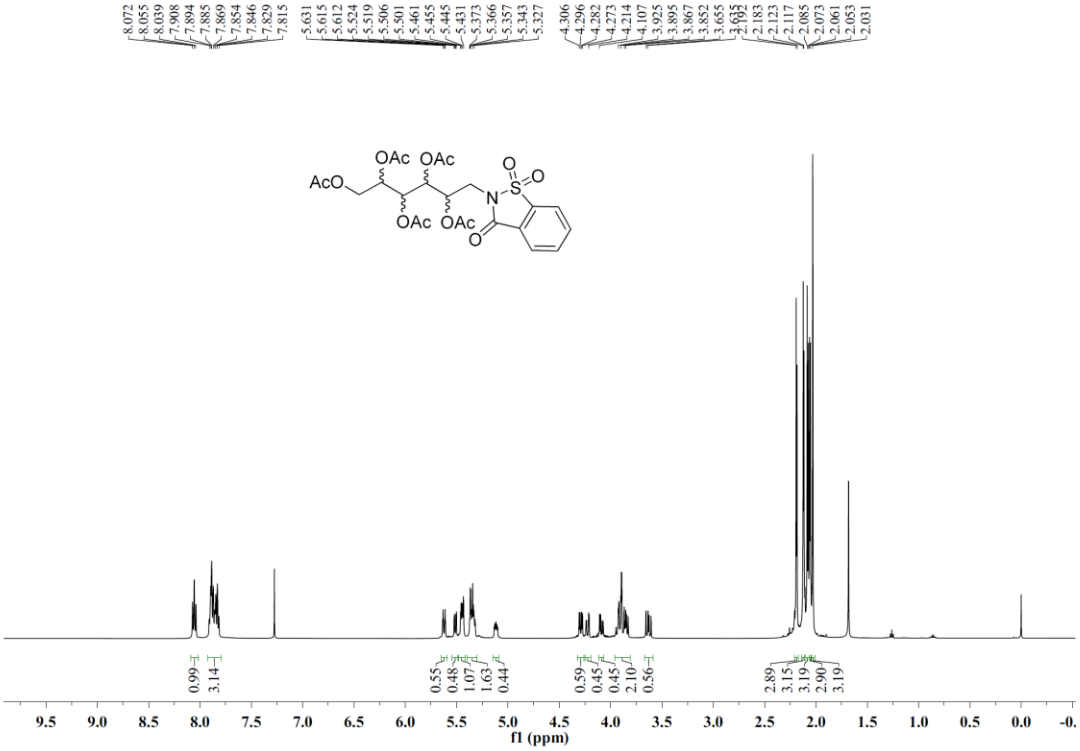

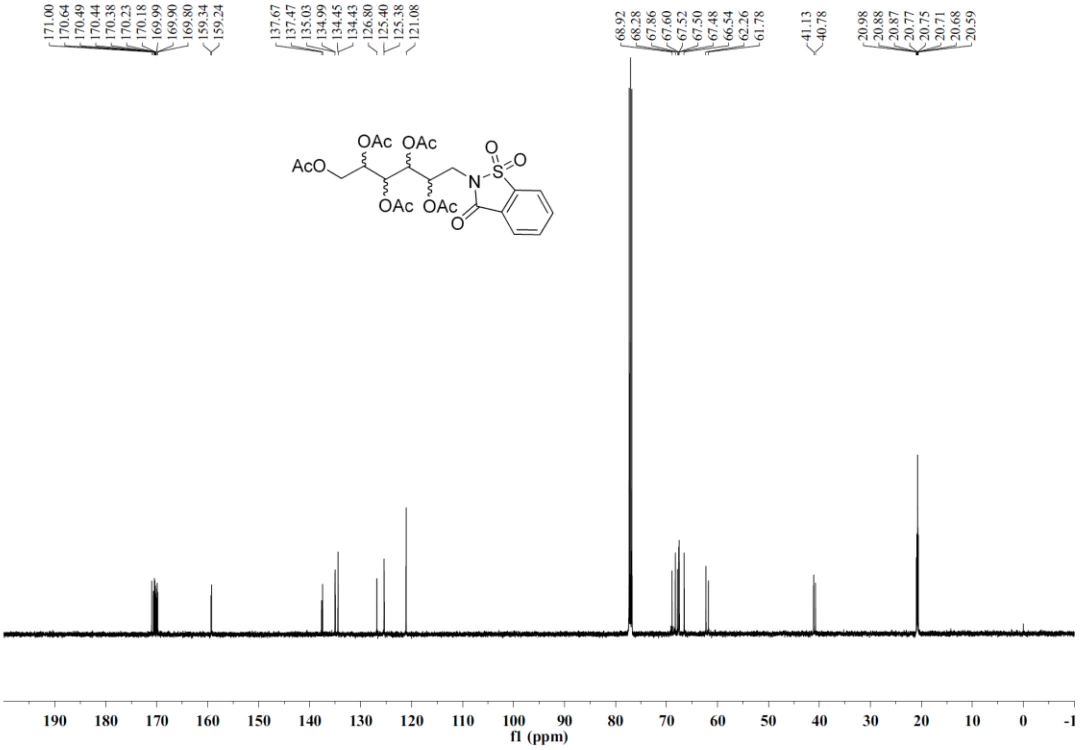

Supplementary Figure 48. NMR spectra of **3rm** (recrystallized from isopropanol)

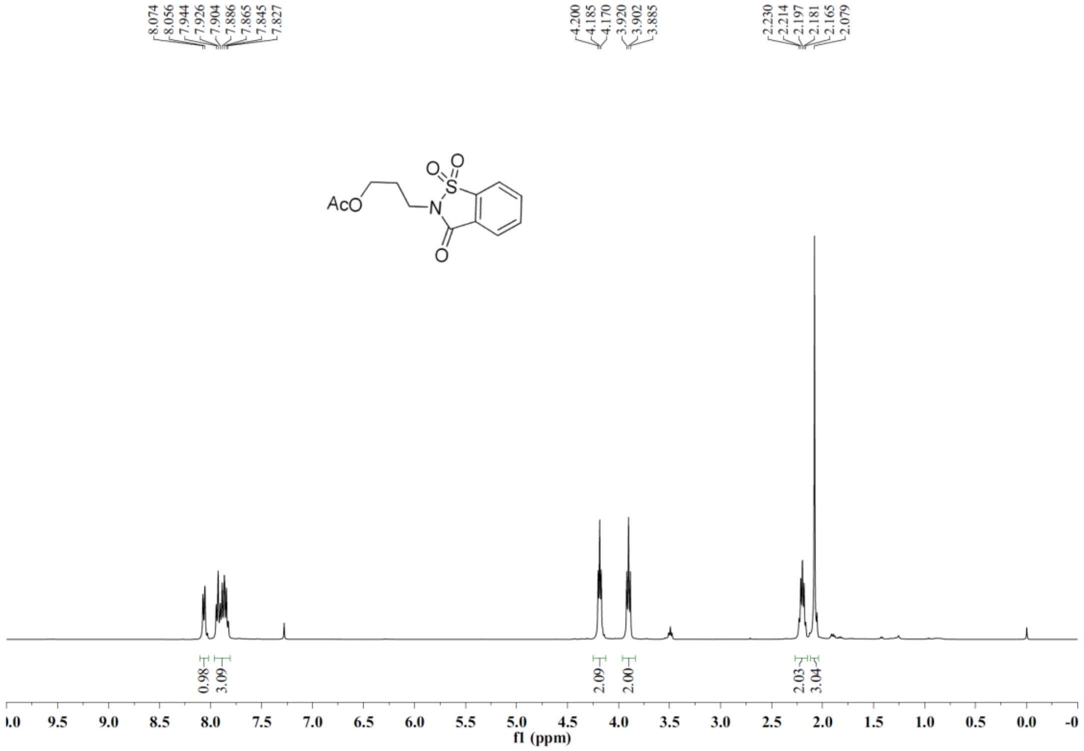

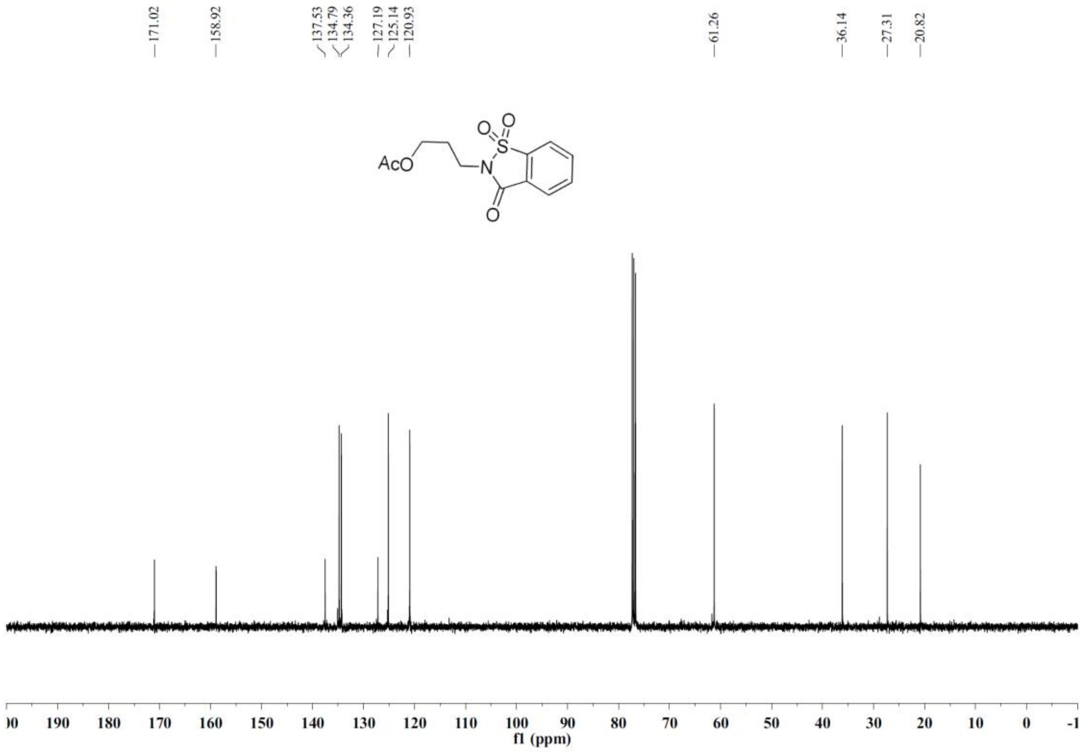

Supplementary Figure 49. NMR spectra of **3sm**

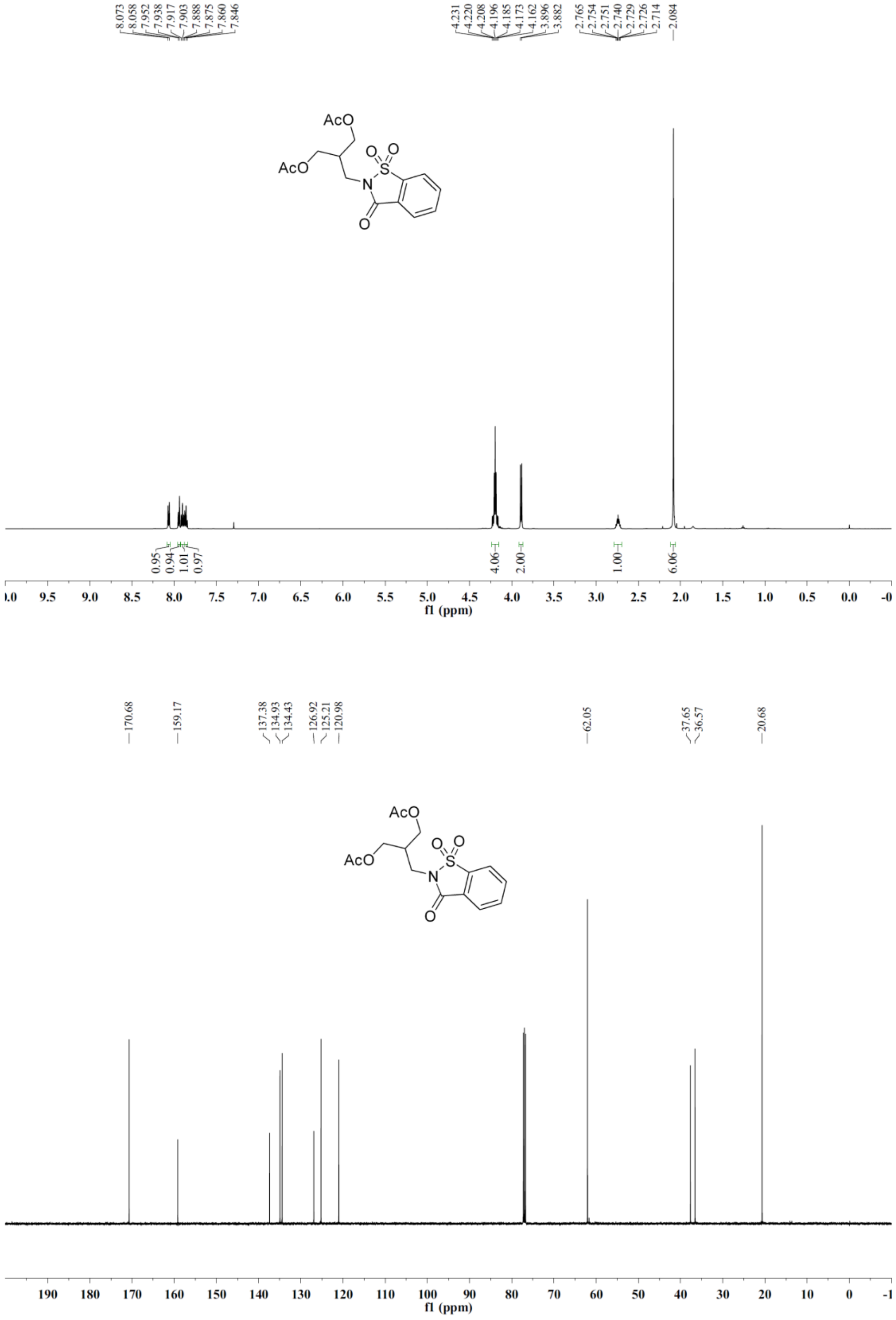

Supplementary Figure 50. NMR spectra of **3tm**

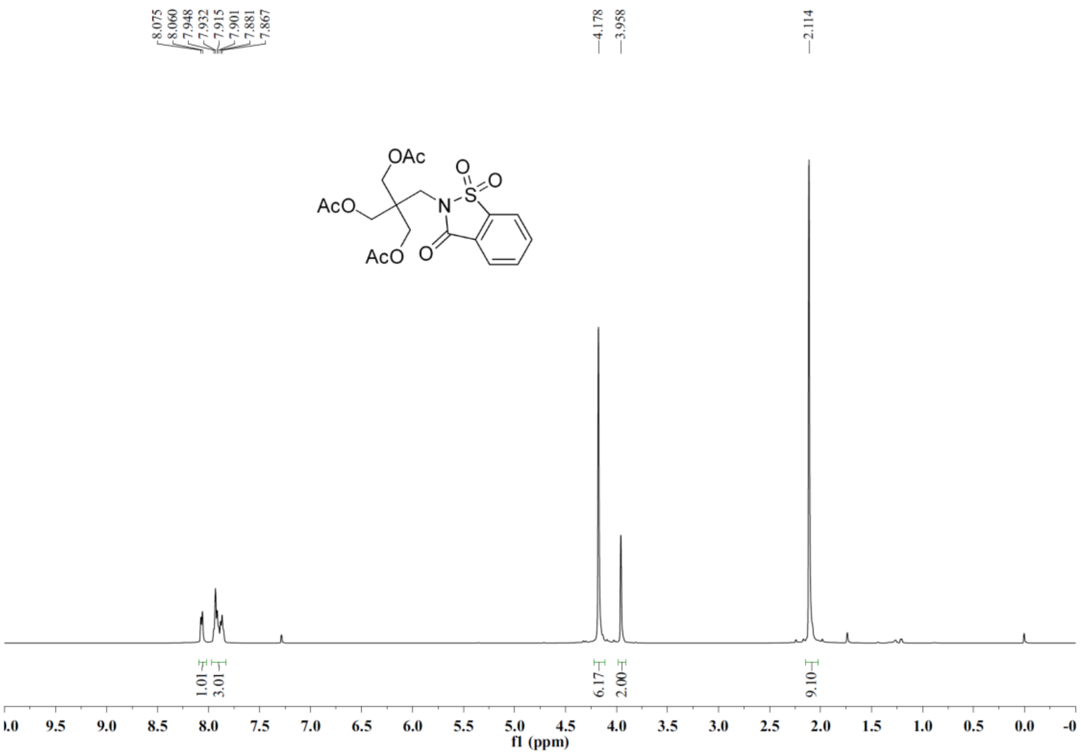

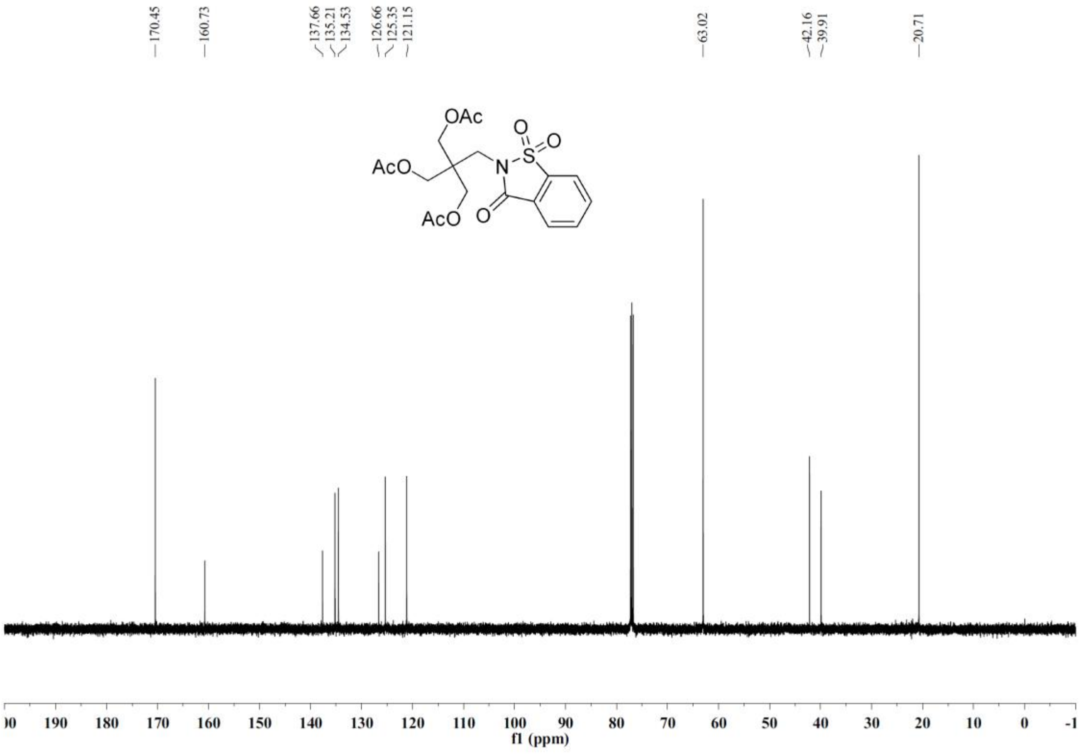

Supplementary Figure 51. NMR spectra of **3um**

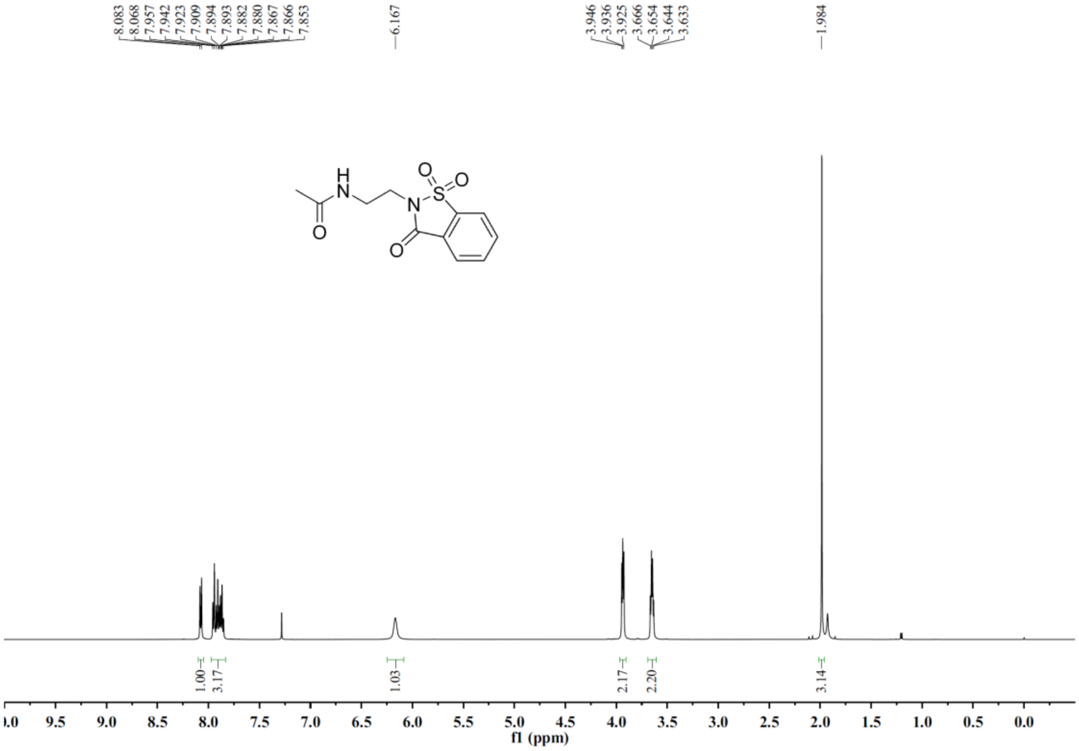

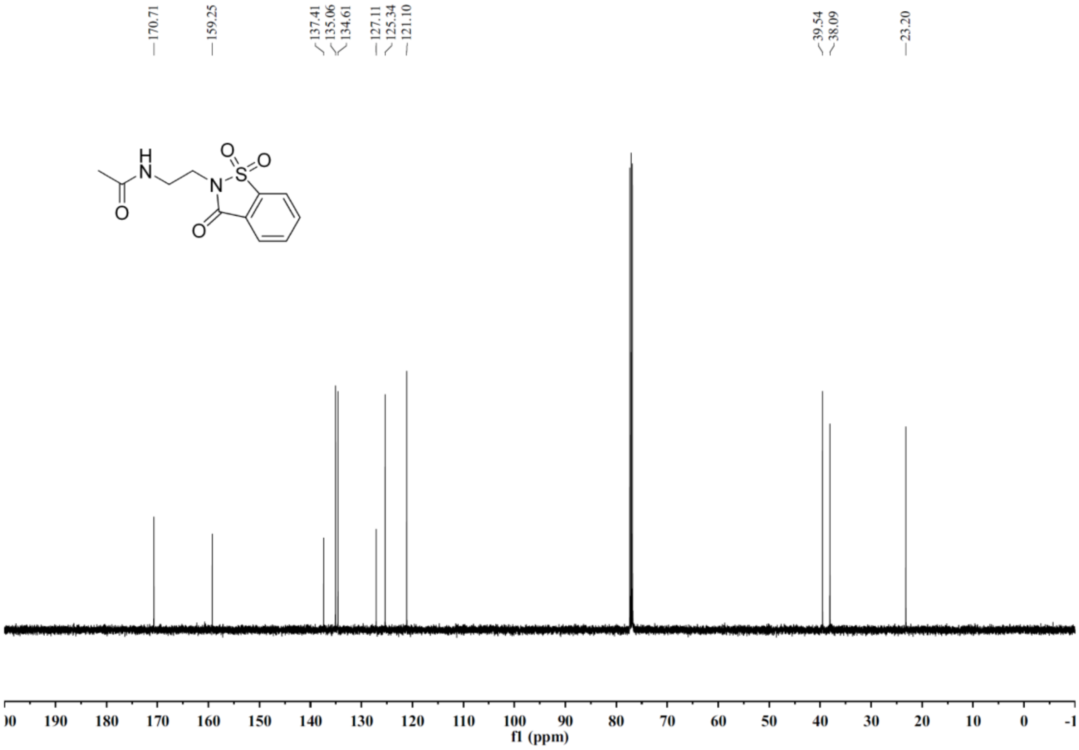

Supplementary Figure 52. NMR spectra of **5am**

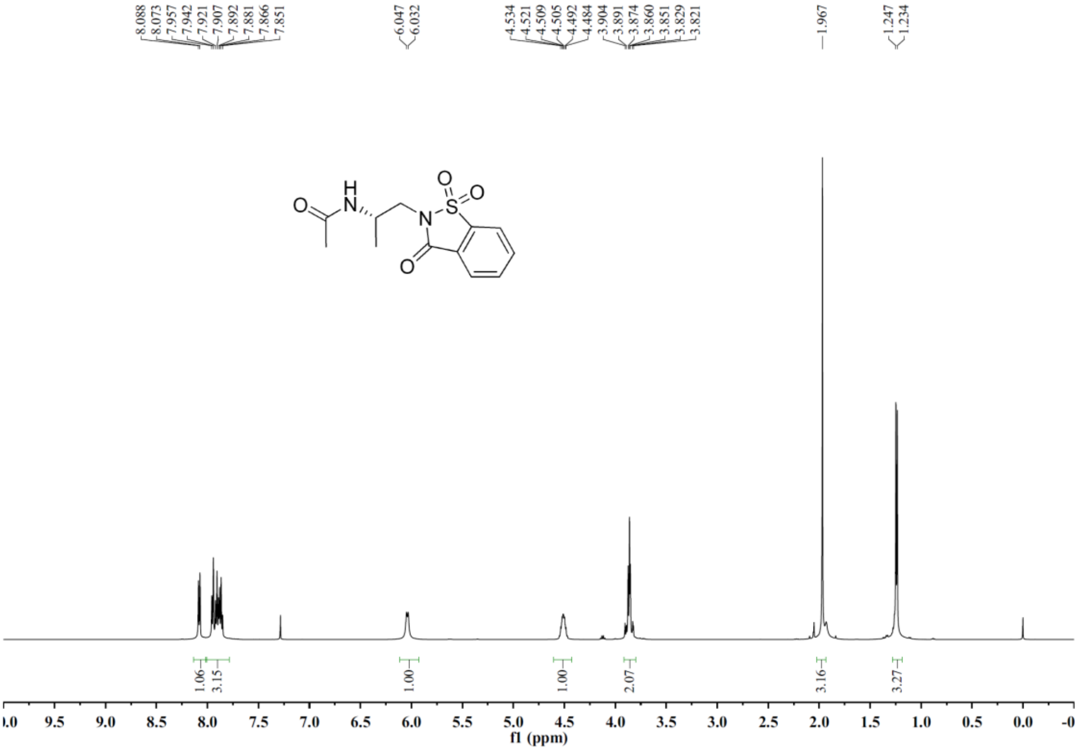

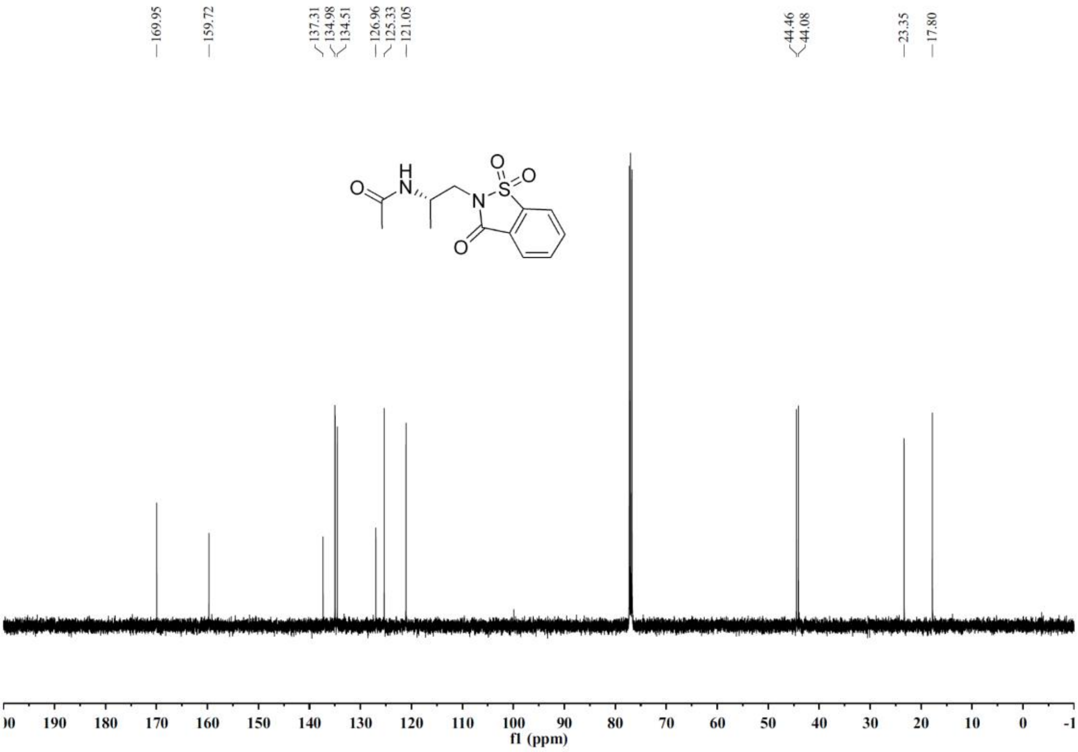

Supplementary Figure 53. NMR spectra of **5bm**

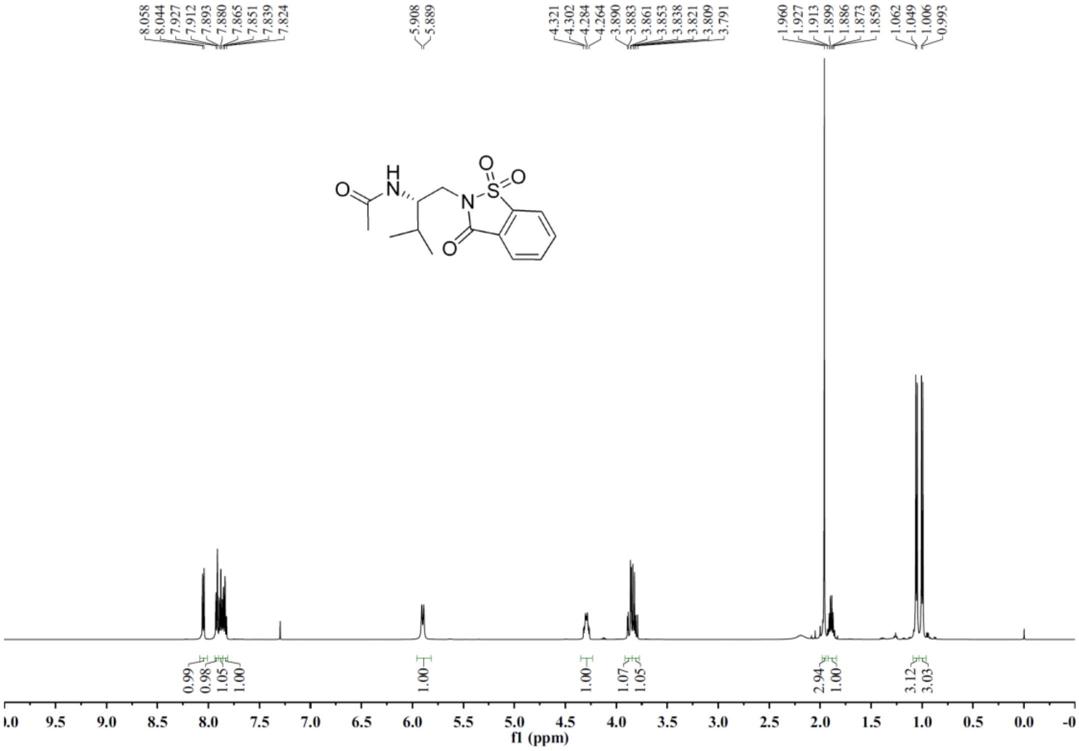

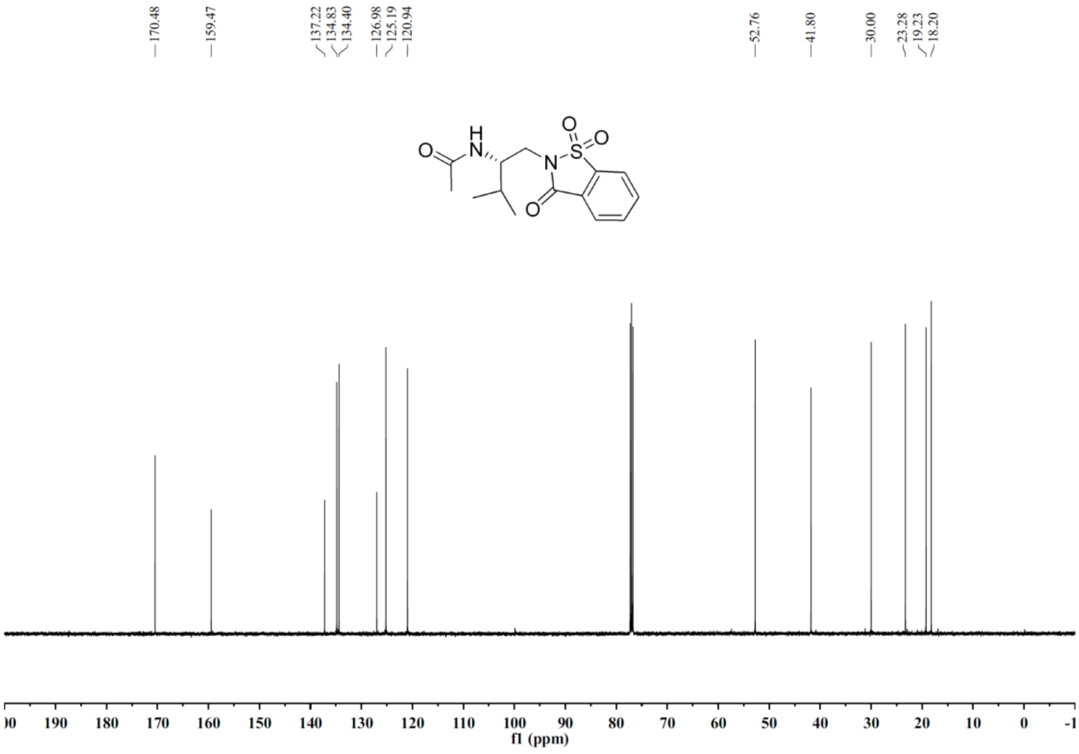

Supplementary Figure 54. NMR spectra of **5cm**

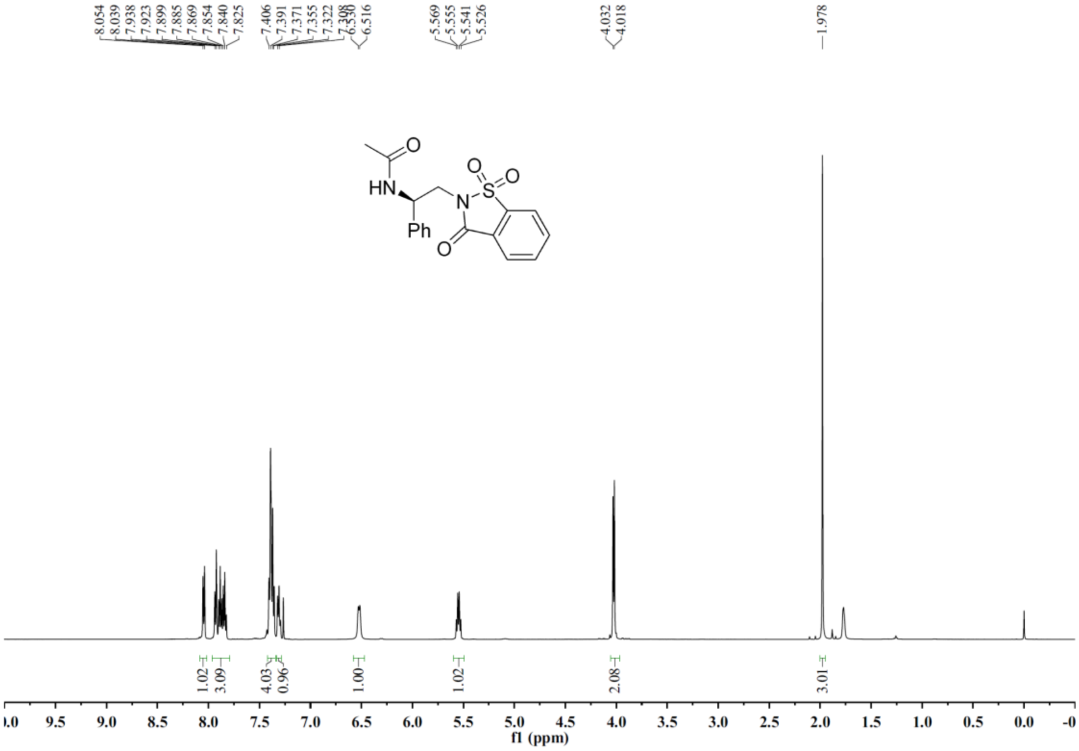

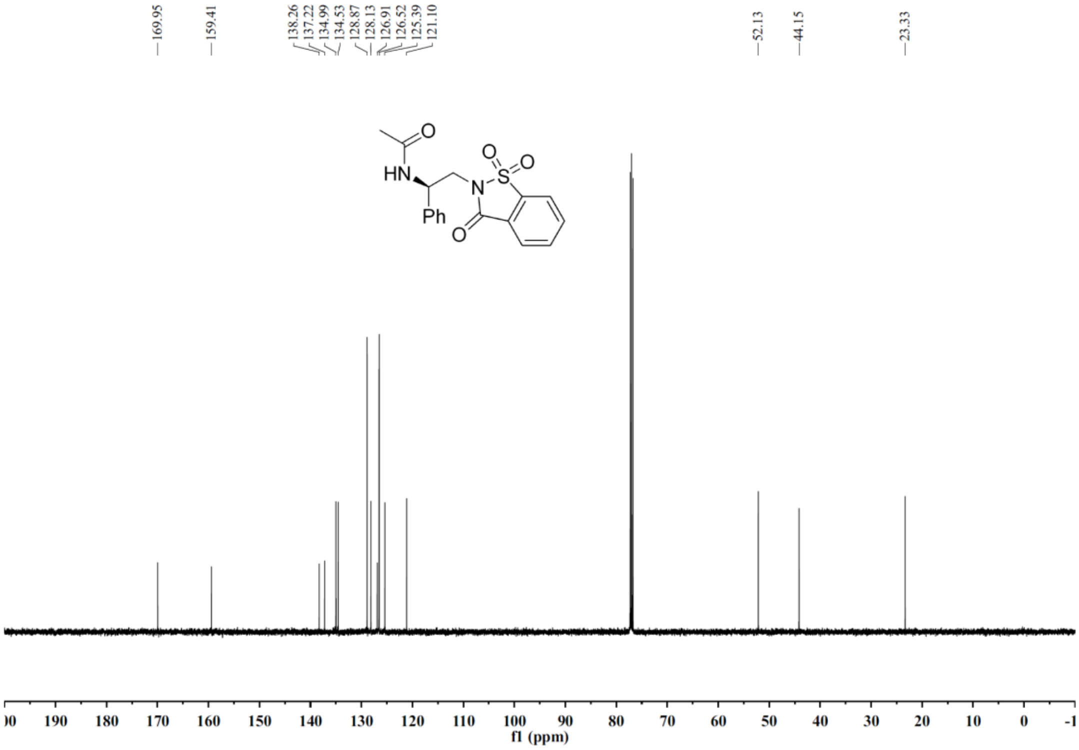

Supplementary Figure 55. NMR spectra of **5dm**

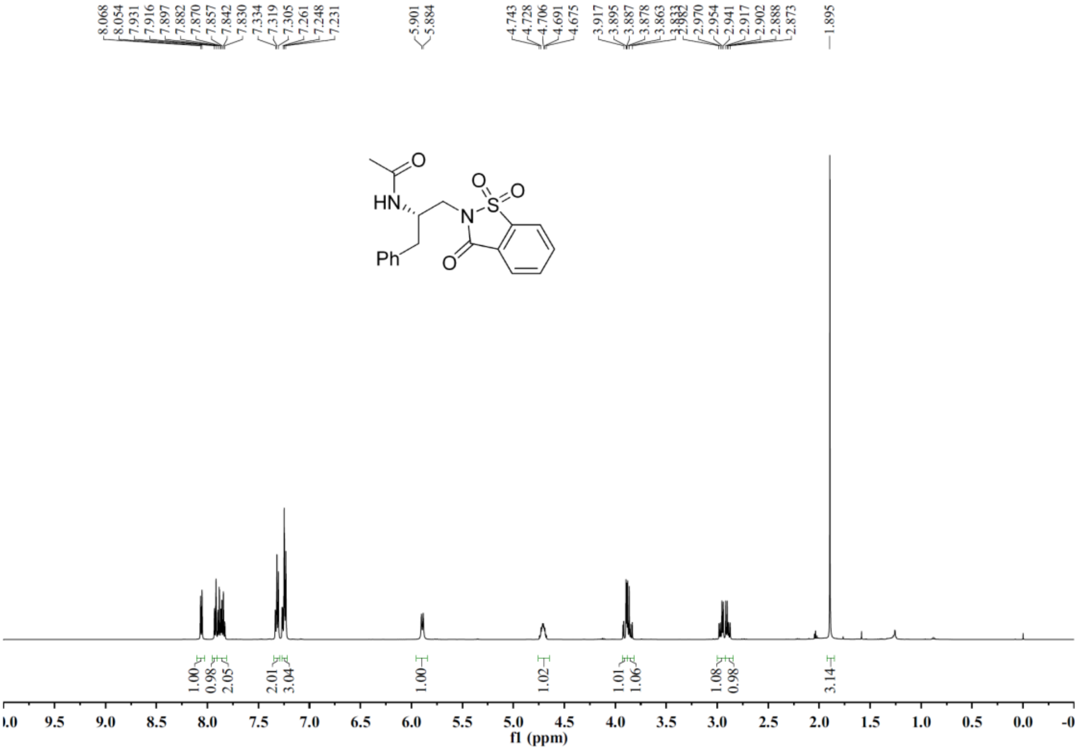

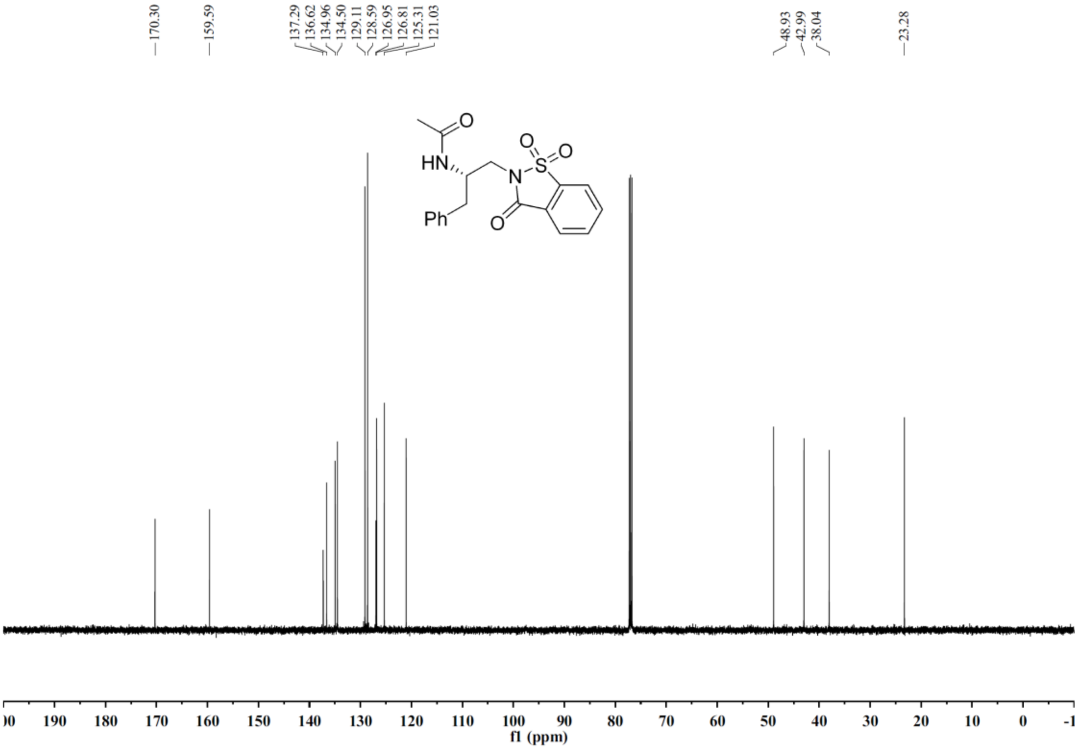

Supplementary Figure 56. NMR spectra of **5em**

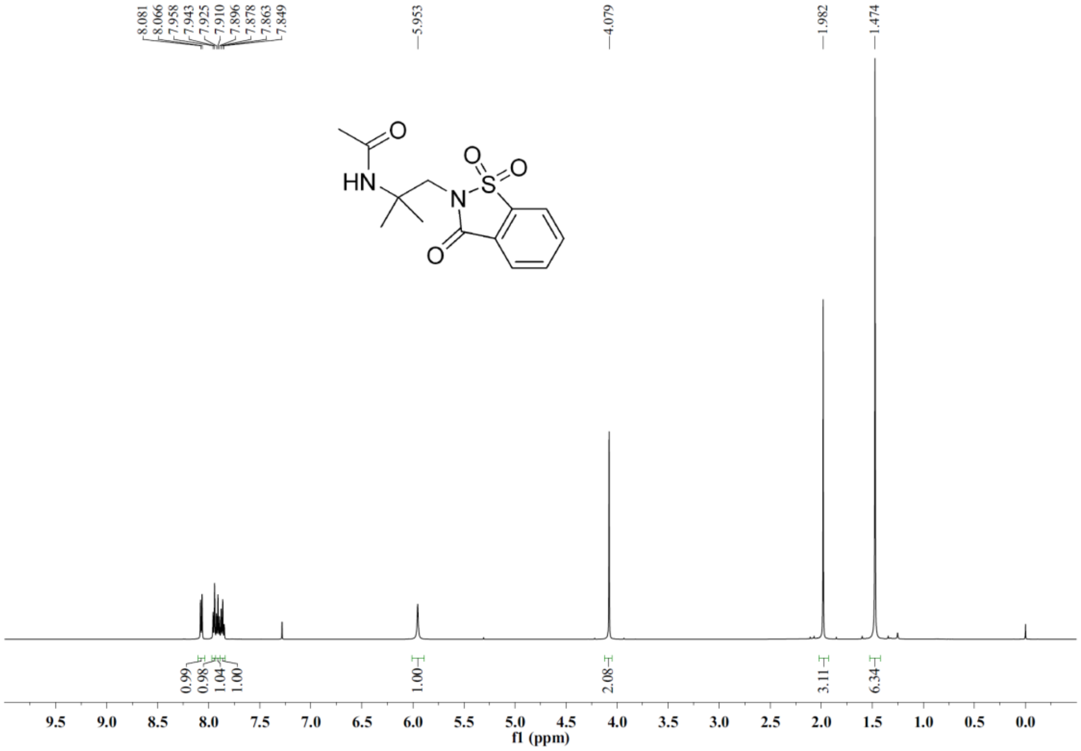

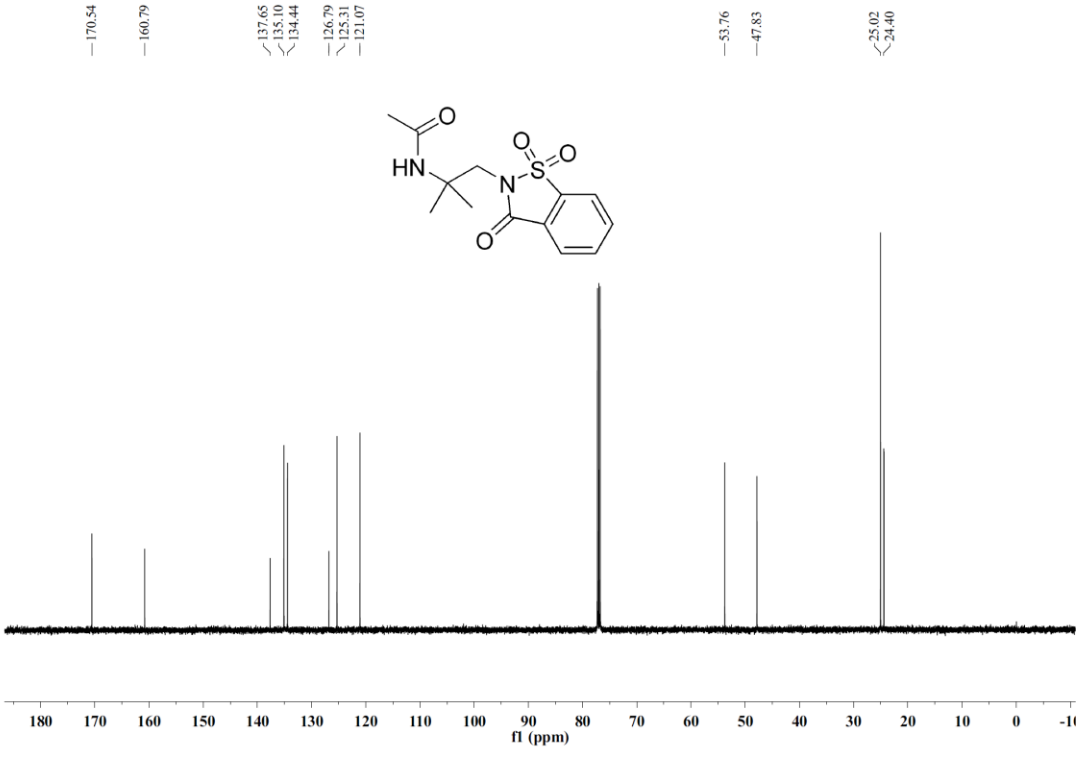

Supplementary Figure 57. NMR spectra of **5fm**

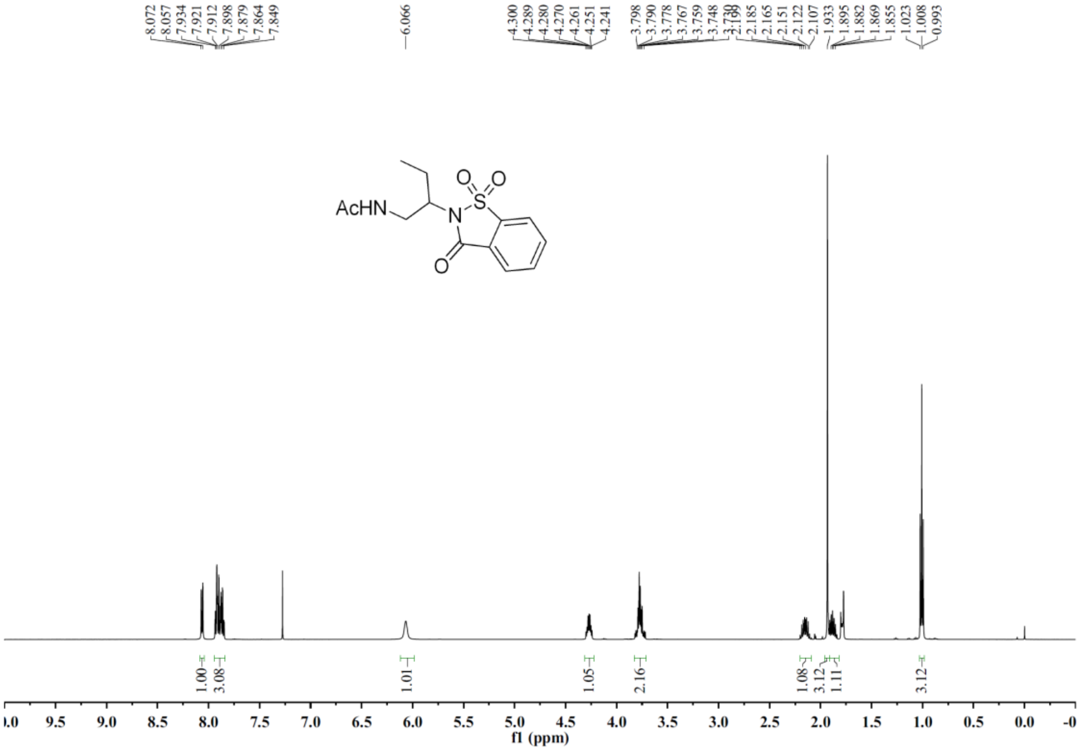

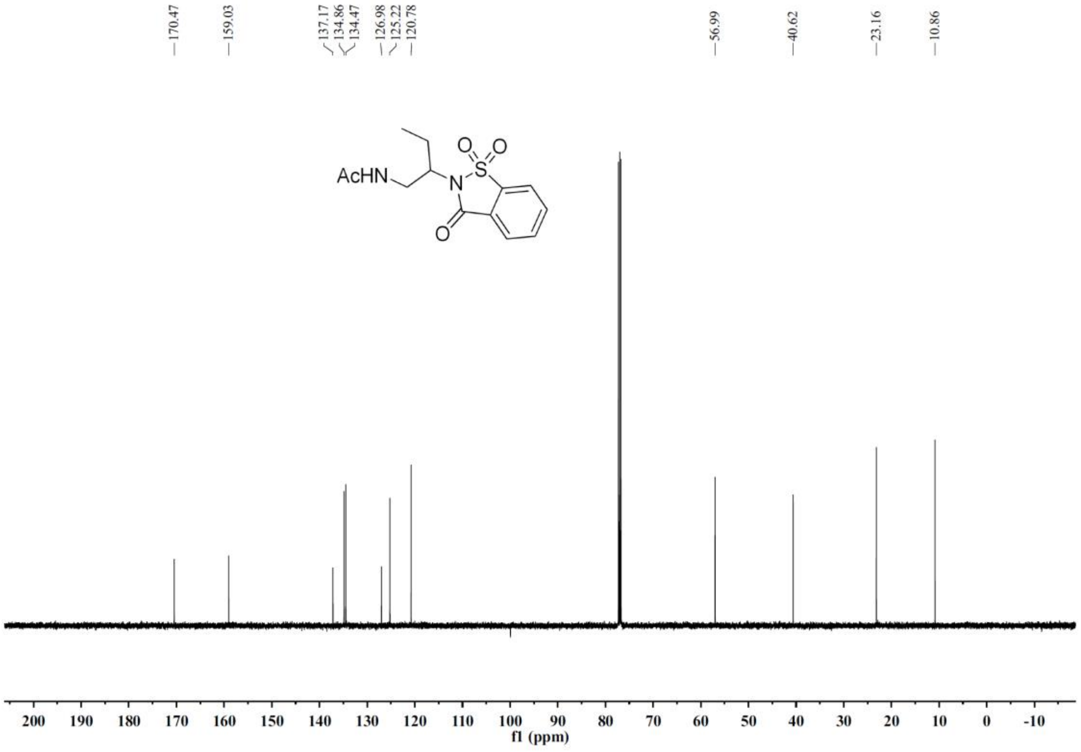

Supplementary Figure 58. NMR spectra of **5gm**

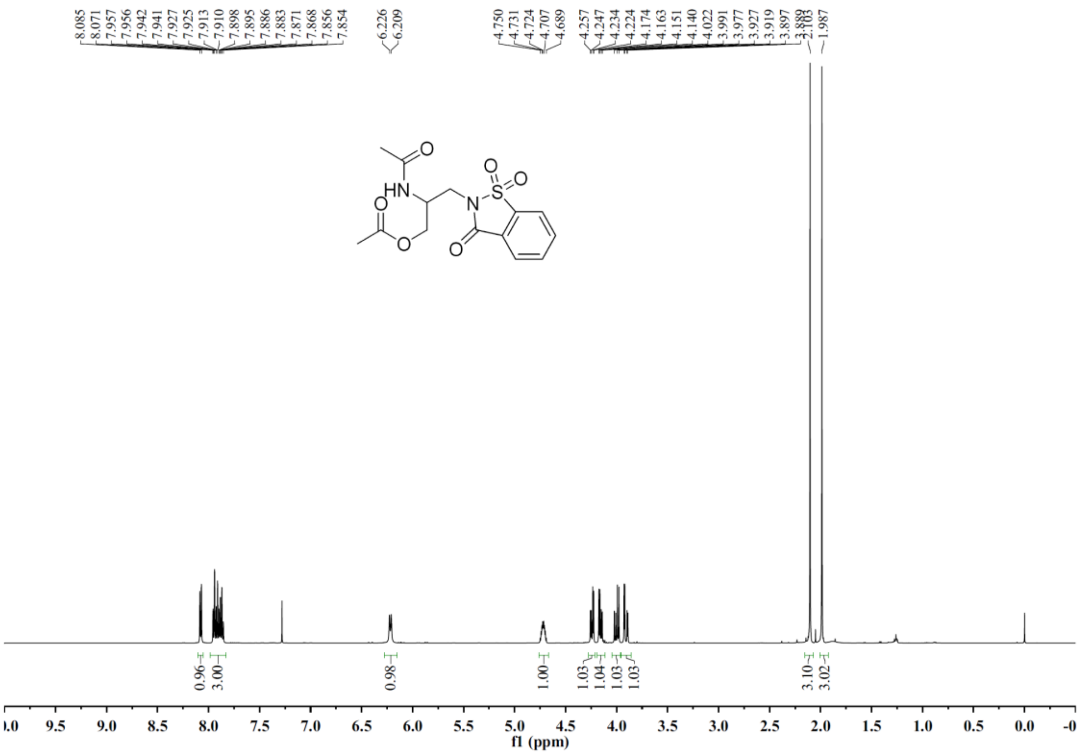

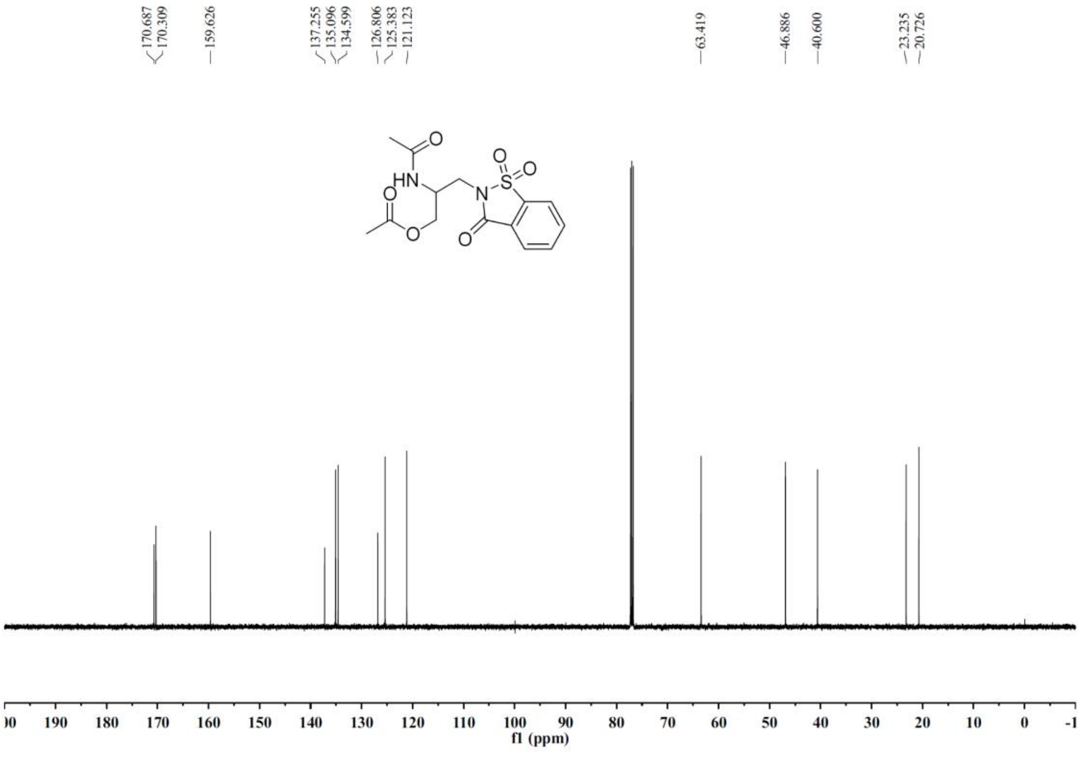

Supplementary Figure 59. NMR spectra of **5hm**

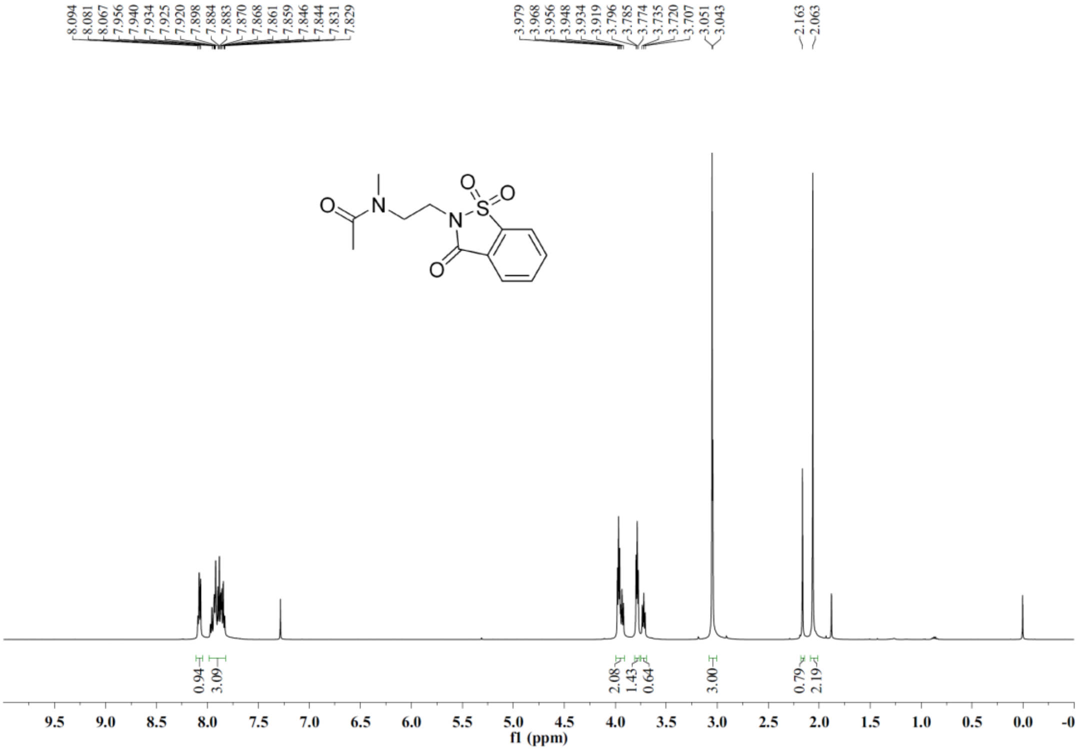

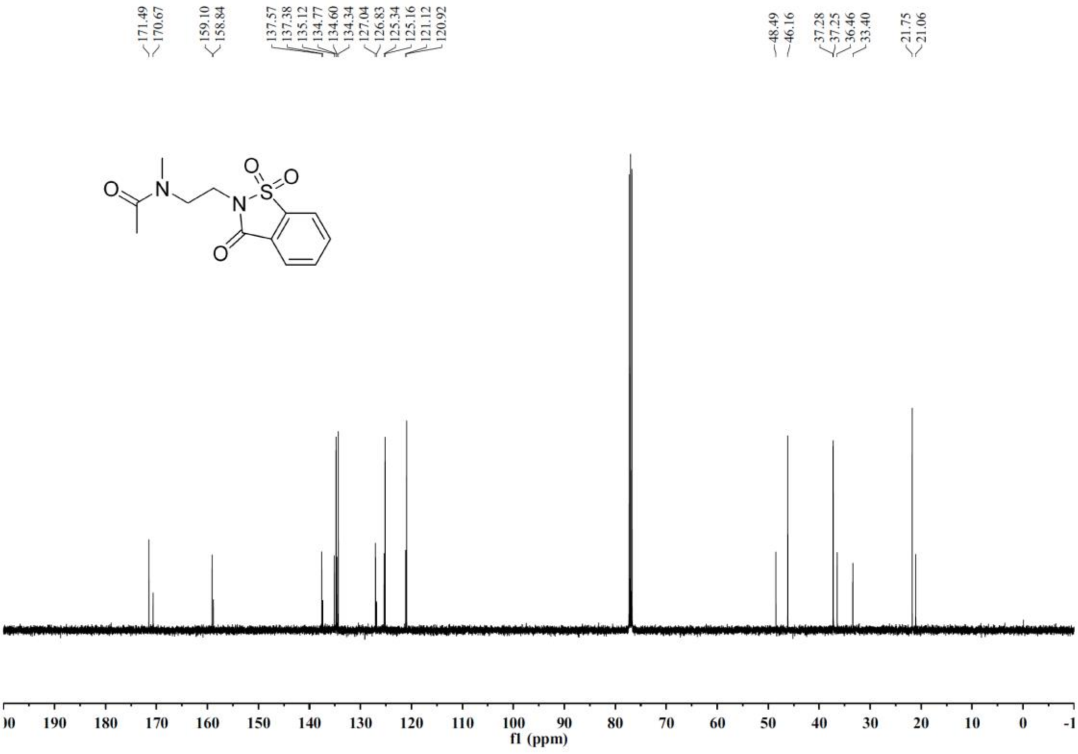

Supplementary Figure 60. NMR spectra of **5im**

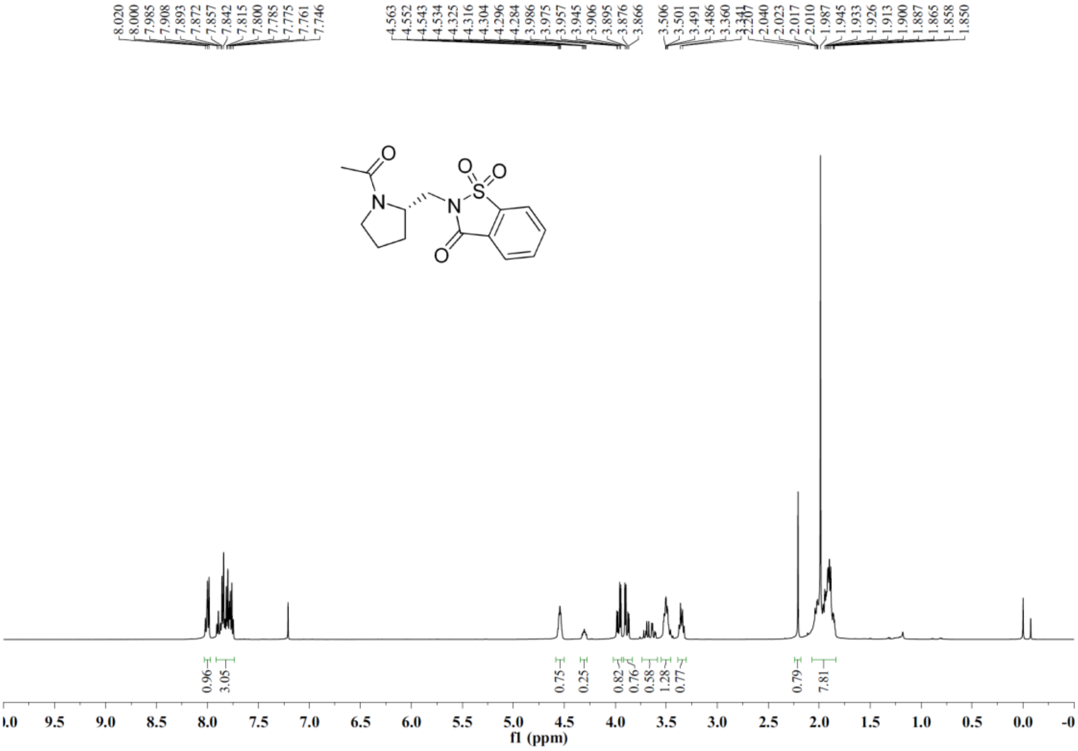

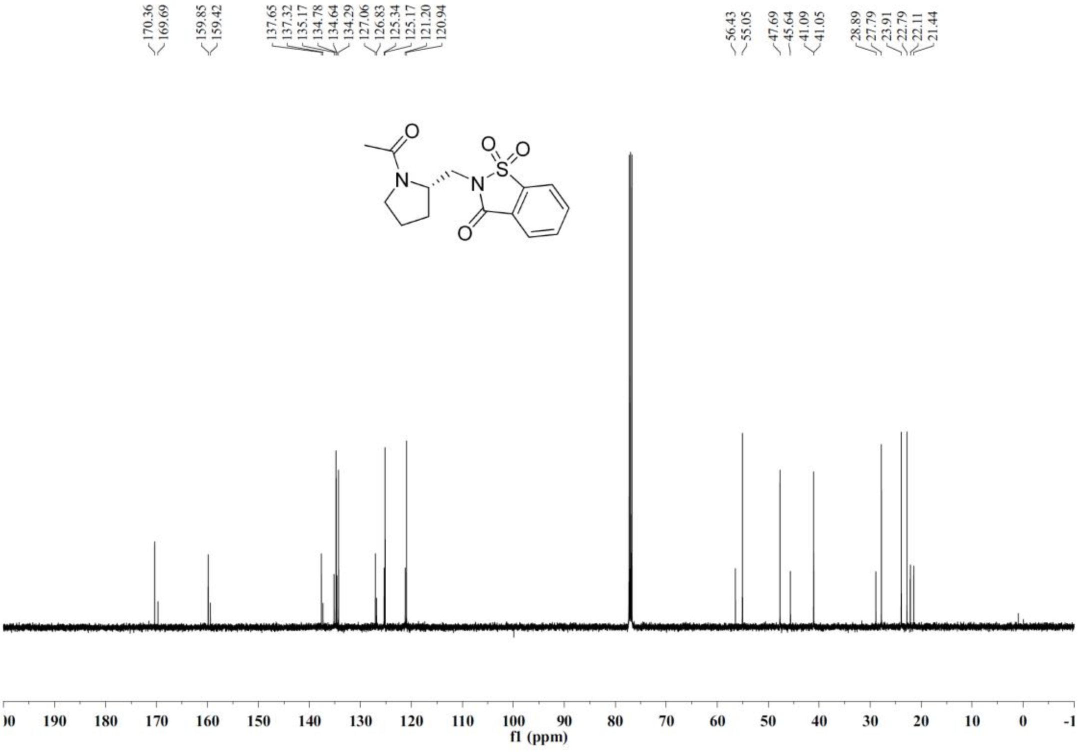

Supplementary Figure 61. NMR spectra of **5jm**

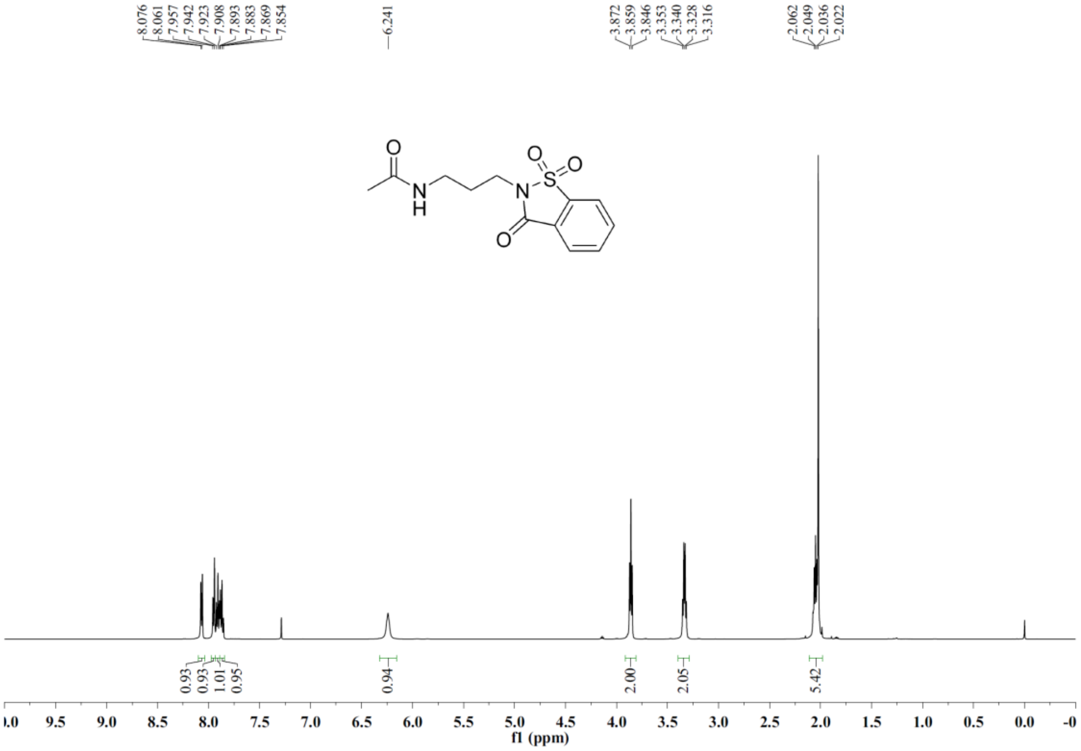

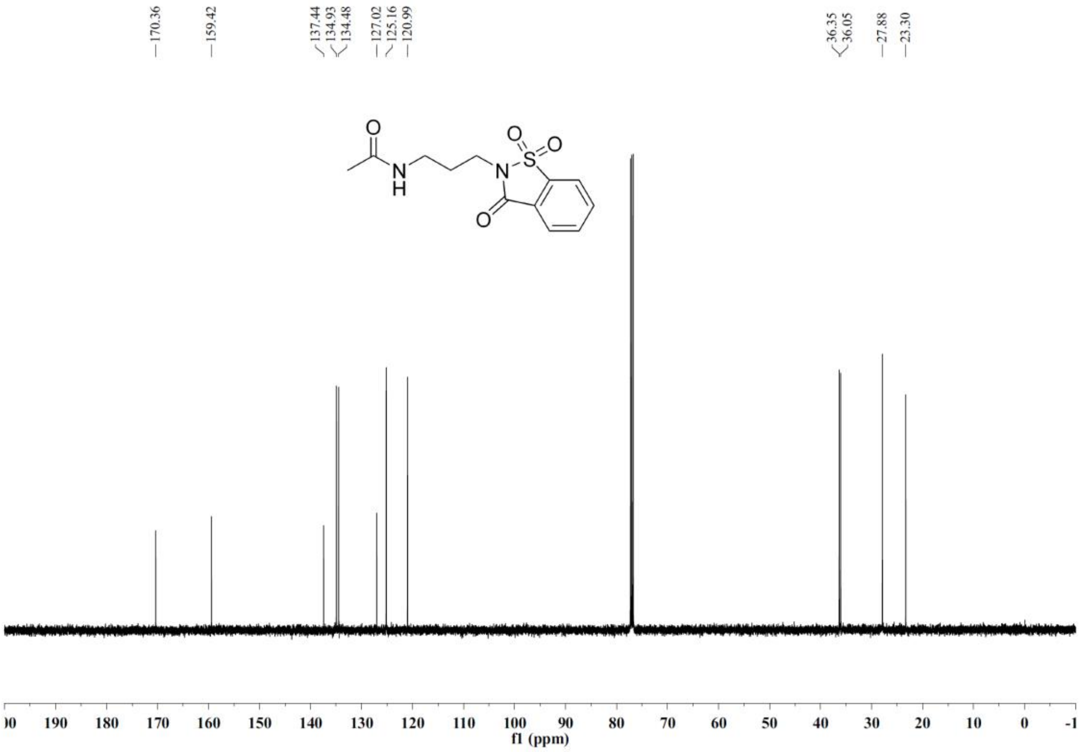

Supplementary Figure 62. NMR spectra of **5km**

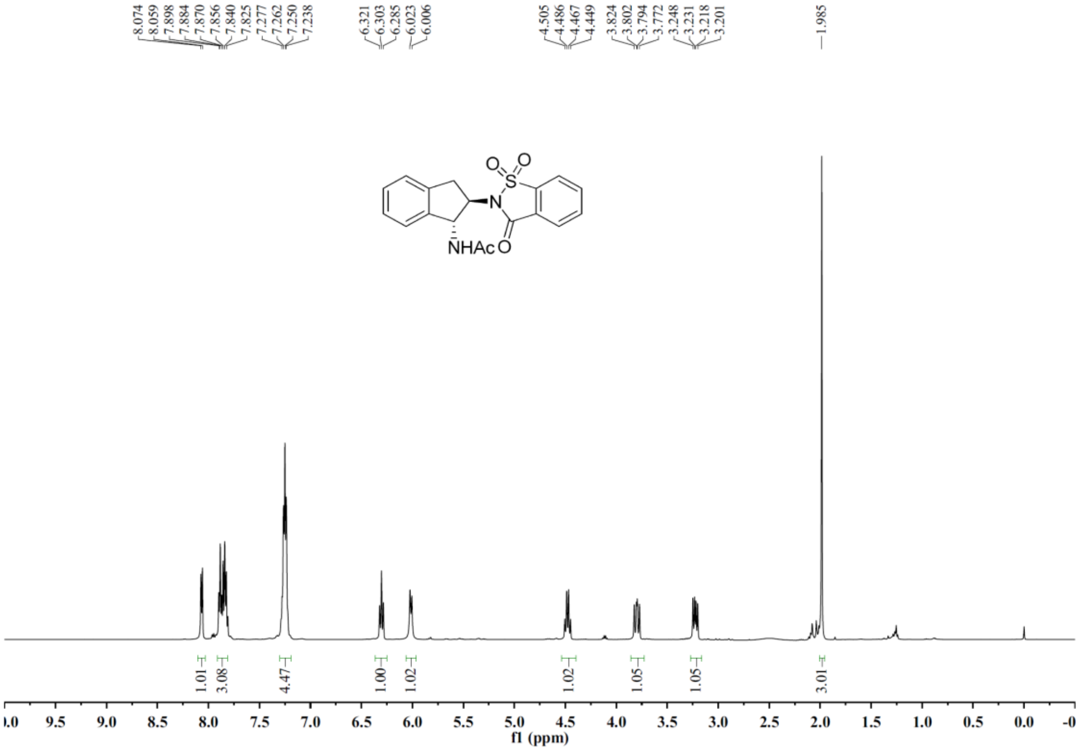

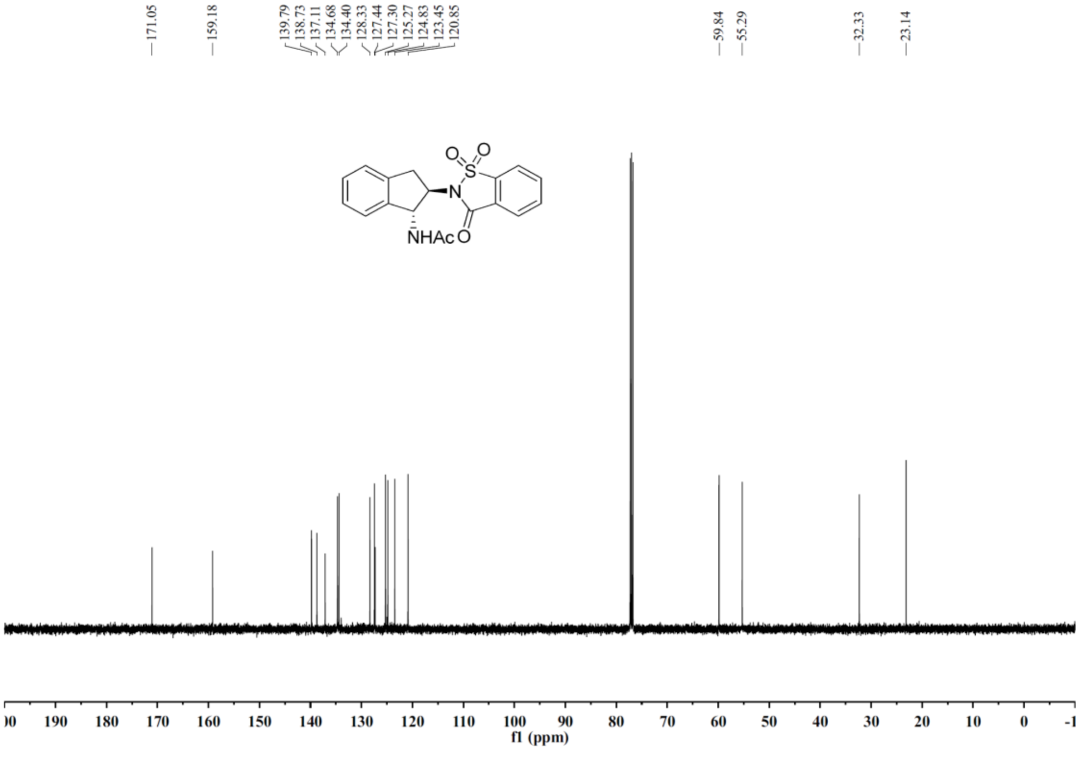

Supplementary Figure 63. NMR spectra of **5lm**

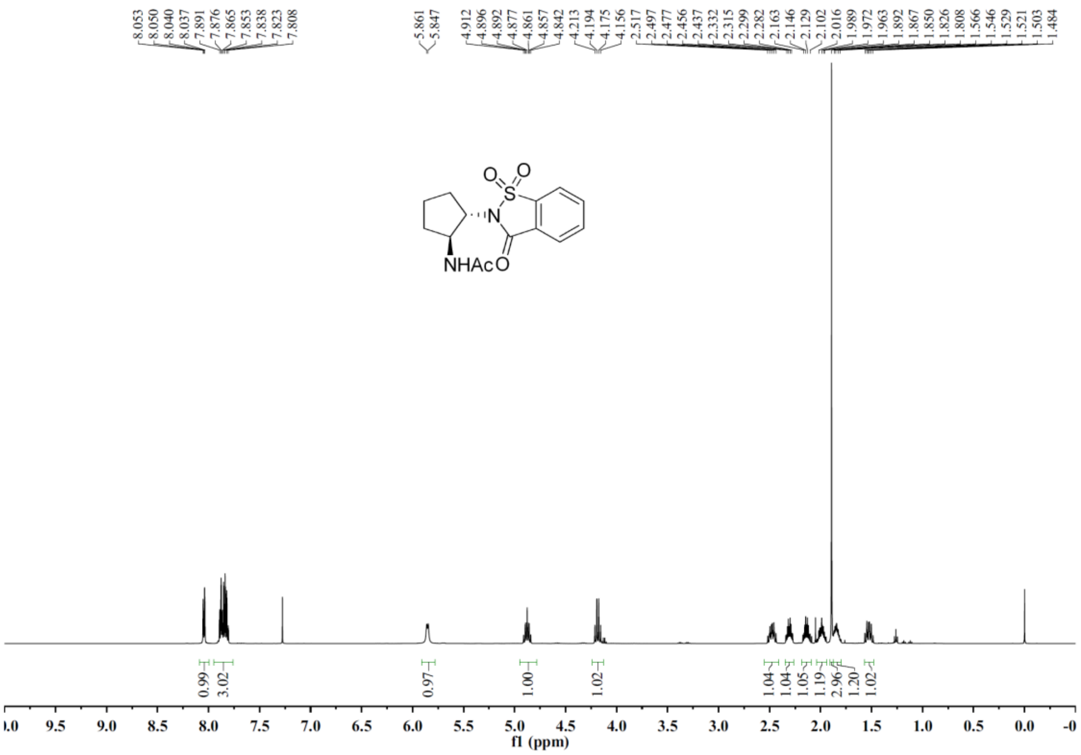

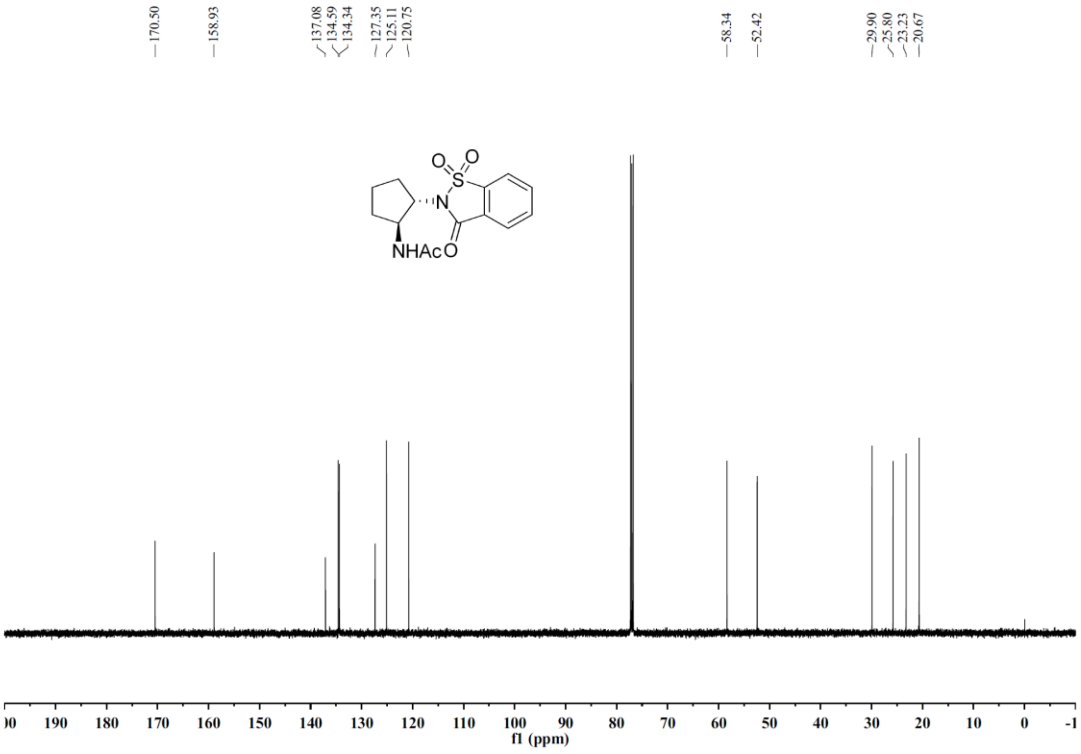

Supplementary Figure 64. NMR spectra of **5mm**

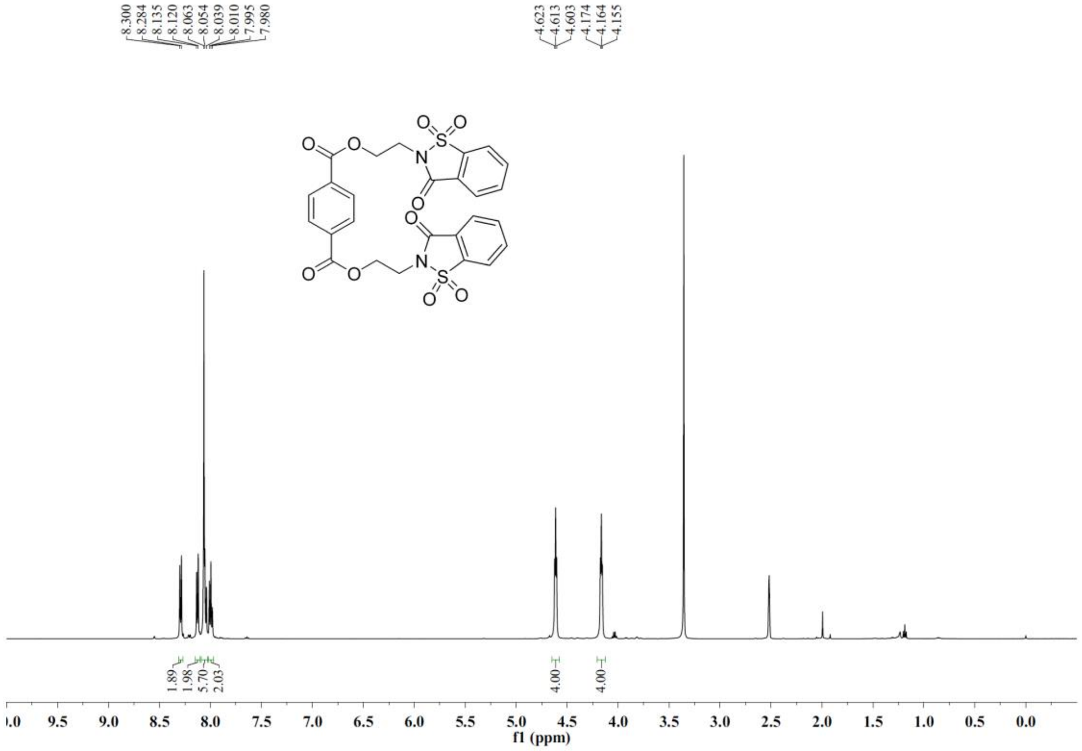

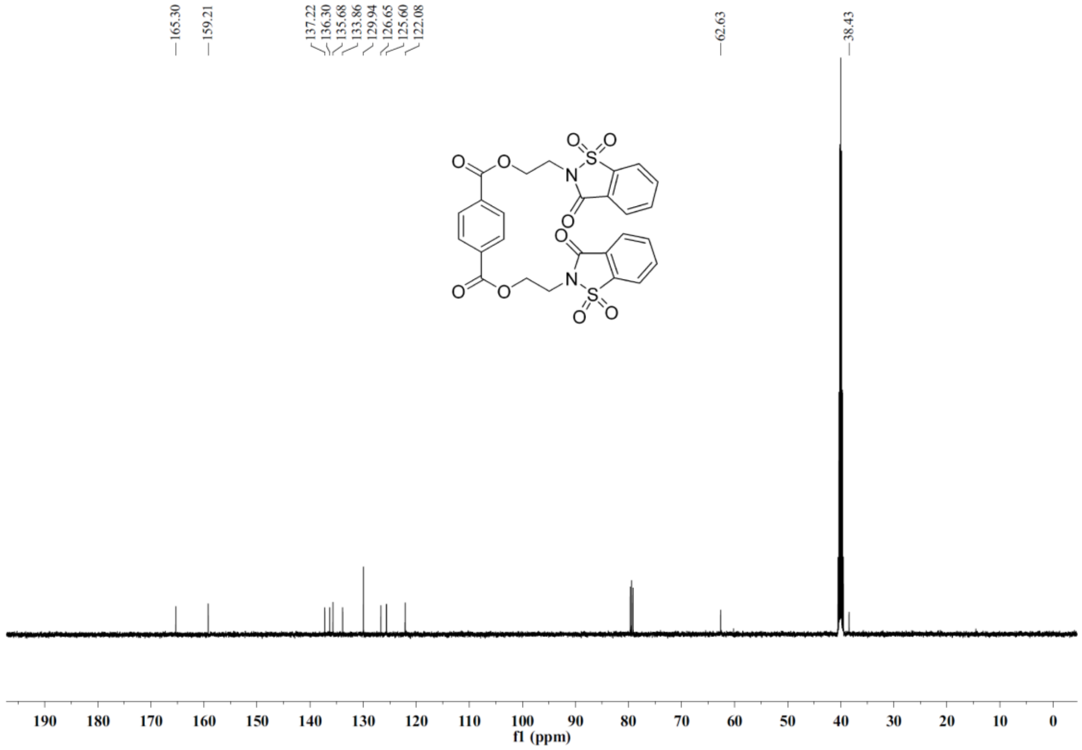

Supplementary Figure 65. NMR spectra of **8**

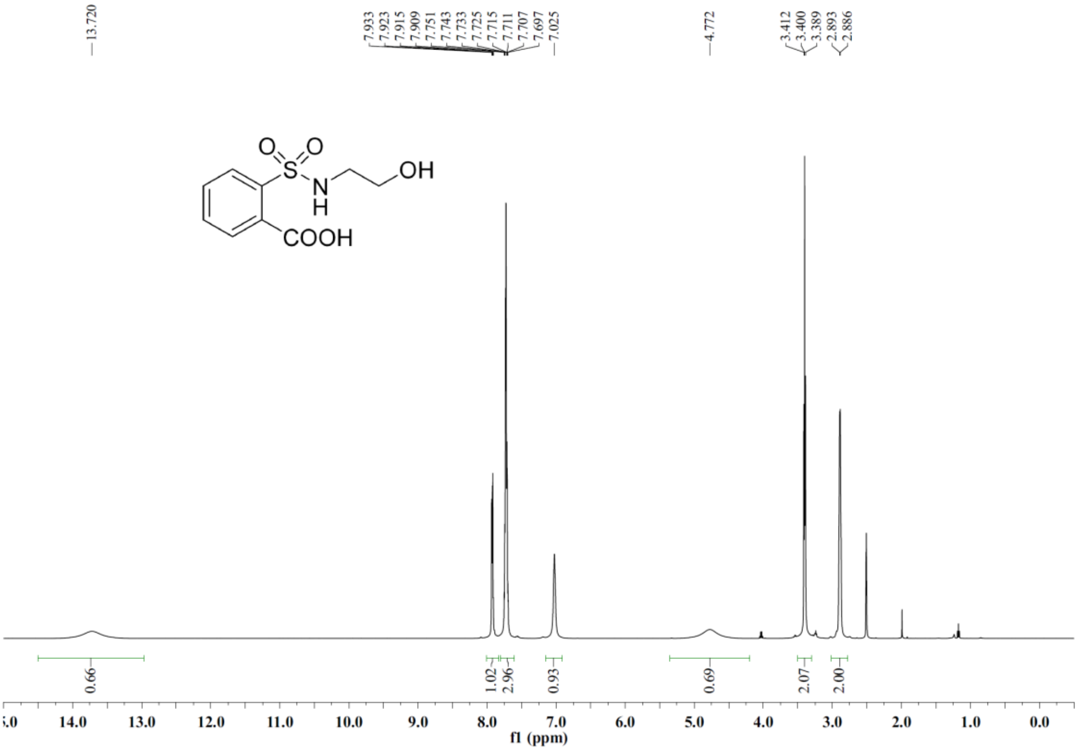

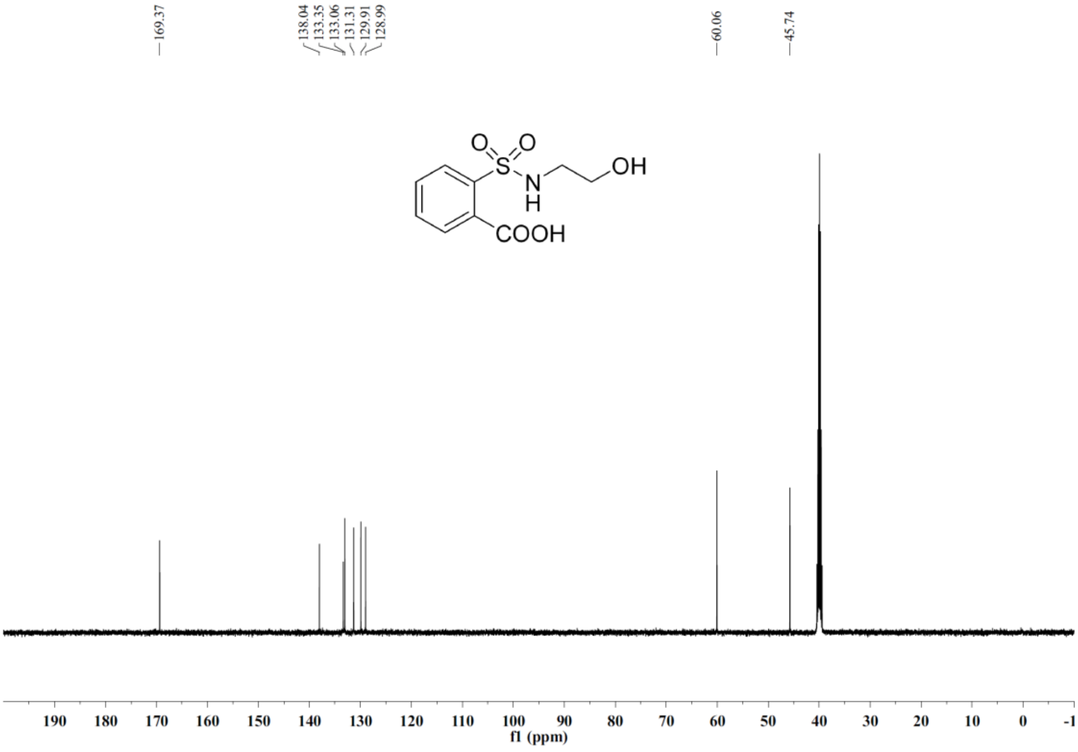

Supplementary Figure 66. NMR spectra of **9**

Supplementary References

[1] J. Kofoed, J.-L. Reymond, T. Darbre, *Org. Biomol. Chem.* **2005**, *3*, 1850-1855.

[2] J. H. Clark, J. Emsley, O. P. A. Hoyte, *J. Chem. Soc., Perkin Trans. 1* **1977**, 1091-1094.

[3] K. Yoshida, T. Furuta, T. Kawabata, *Angew. Chem. Int. Ed.* **2011**, *50*, 4888-4892.

[4] Y. Nishida, Y. Mengfei, K. Fujisawa, S. Kitagawa, H. Dohi, H. Uzawa, *Tetrahedron: Asymmetry* **2017**, *28*, 1435-1443.

[5] D. Wahler, O. Boujard, F. Lefèvre, J.-L. Reymond, *Tetrahedron* **2004**, *60*, 703-710.

[6] J. Seayad, A. M. Seayad, C. L. L. Chai, *Org. Lett.* **2010**, *12*, 1412-1415.

[7] L. Burroughs, M. E. Vale, J. A. R. Gilks, H. Forintos, C. J. Hayes, P. A. Clarke, *Chem. Commun.* **2010**, *46*, 4776-4778.

[8] S. Jarosz, S. Skóra, K. Szewczyk, Z. Ciunik, *Tetrahedron: Asymmetry* **2001**, *12*, 1895-1905.

[9] Y. Tanoue, M. Hamada, N. Kai, T. Nagai, K. Sakata, M. Hashimoto, S.-I. Morishita, *J. Heterocyclic Chem.* **2000**, *37*, 1351-1353.

[10] Y. Nishio, R. Mifune, T. Sato, S.-i. Ishikawa, H. Matsubara, *Tetrahedron Lett.* **2017**, *58*, 1190-1193.

[11] T. Yamaguchi, D. Hesek, M. Lee, A. G. Oliver, S. Mobashery, *J. Org. Chem.* **2010**, *75*, 3515-3517.

[12] V. Leiro, J. M. Seco, E. Quinoa, R. Riguera, *Chem. Asian. J.* **2010**, *5*, 2106-2112.

[13] S. Santoso, T. Kemmer, W. Trowitzsch, *Liebigs Ann. Chem.* **1981**, *1981*, 642-657.

[14] Y. Fu, H. Fu, F. Ye, J. Mao, X. Wen, *Synth. Commun.* **2009**, *39*, 2454-2463.

[15] J. Li, R. H. Grubbs, B. M. Stoltz, *Org. Lett.* **2016**, *18*, 5449-5451.

[16] P. Chen, J. Qu, *J. Org. Chem.* **2011**, *76*, 2994-3004.

[17] H. Paulsen, H. Behre, *Chem. Ber.* **1971**, *104*, 1264-1280.

[18] A. Pelter, M. E. Colclough, *Tetrahedron* **1995**, *51*, 811-828.
